# Structural Influence of the Chemical Fueling System on a Catalysis-Driven Rotary Molecular Motor

**DOI:** 10.1021/jacs.5c00028

**Published:** 2025-02-27

**Authors:** Hua-Kui Liu, Toufic W. Mrad, Axel Troncossi, Stefan Borsley, Benjamin M. W. Roberts, Alexander Betts, David A. Leigh

**Affiliations:** 1 Department of Chemistry, University of Manchester, Manchester M13 9PL, United Kingdom; 2 School of Chemistry and Molecular Engineering, East China Normal University, Shanghai 200062, China

## Abstract

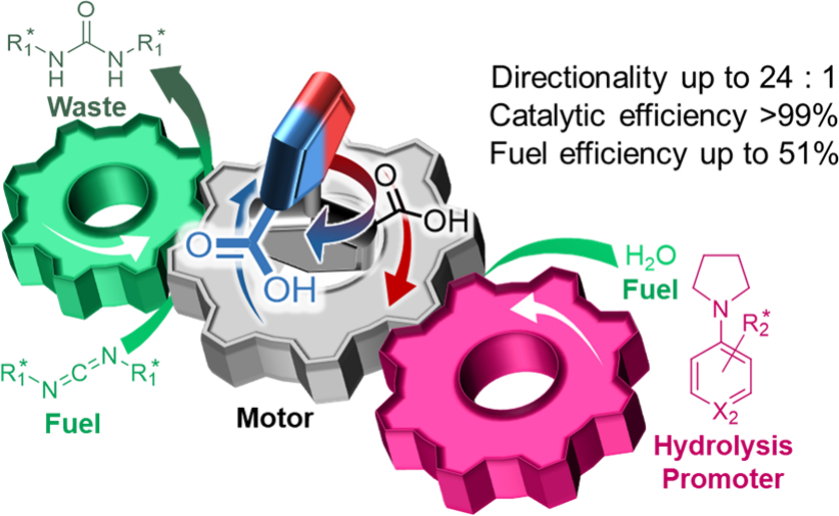

Continuous directionally biased 360° rotation about a covalent single bond was recently realized in the form of a chemically fueled 1-phenylpyrrole 2,2′-dicarboxylic acid rotary molecular motor. However, the original fueling system and reaction conditions resulted in a motor directionality of only ∼3:1 (i.e., on average a backward rotation for every three forward rotations), along with a catalytic efficiency for the motor operation of 97% and a fuel efficiency of 14%. Here, we report on the efficacy of a series of chiral carbodiimide fuels and chiral hydrolysis promoters (pyridine and pyridine *N*-oxide derivatives) in driving improved directional rotation of this motor-molecule. We outline the complete reaction network for motor operation, composed of directional, futile, and slip cycles. Using derivatives of the motor where the final conformational step in the 360° rotation is either very slow or completely blocked, the phenylpyrrole diacid becomes enantiomerically enriched, allowing the kinetic gating of the individual steps in the catalytic cycle to be measured. The chiral carbodiimide fuel that produces the highest directionality gives 13% enantiomeric excess (*e.e.*) for the anhydride-forming kinetically gated step, while the most effective chiral hydrolysis promoter generates 90% *e.e*. for the kinetically gated hydrolysis step. Combining the best-performing fuel and hydrolysis promoter into a single fueling system results in a 92% *e.e.*. Under a dilute chemostated fueling regime (to avoid *N*-acyl urea formation at high carbodiimide concentrations with pyridine *N*-oxide hydrolysis promoters), the motor continuously rotates with a directionality of ∼24:1 (i.e., a backward rotation for every 24 forward rotations) with a catalytic efficiency of >99% and a fuel efficiency of 51%.

## Introduction

The development of artificial catalysis-driven molecular motors^1−6^ and pumps^7−10^ is validating mathematical descriptors^11−20^ of their mechanisms and helping establish broadly applicable principles for their design.^20^ Most of the artificial small-molecule motors developed to date are either driven by light,^21−28^ or require multistep chemical synthesis to achieve a single motor cycle,^29−39^ or operate through repetitive oscillation of the environmental conditions (e.g., pH, electric potential, etc.).^40−43^ Such mechanisms are fundamentally different^44−47^ to the catalysis-driven information ratchet mechanisms^12,20,48^ that power biological machinery.^49,50−56^ Motor proteins transduce energy from the motor-molecule’s catalysis of a fuel-to-waste reaction^44^ (typically ATP to ADP). The motor-molecules catalysis proceeds in a mechano-dependent manner, such that the catalytic cycle is kinetically asymmetric.^13,14^ This results in directionally biased dynamics (i.e., directional rotation or translation) of the motor components during the action of catalysis.^13^

Artificial catalysis-driven molecular machines operate through the same type of information ratchet mechanisms^20^ as their biological counterparts.^48^ However, since they are unencumbered by the complexities introduced through evolution, they can have much simpler working designs. This has allowed the fundamental mechanisms by which chemical energy is transduced through catalysis to do mechanical work to be demonstrated.^6^

**Figure 1:**
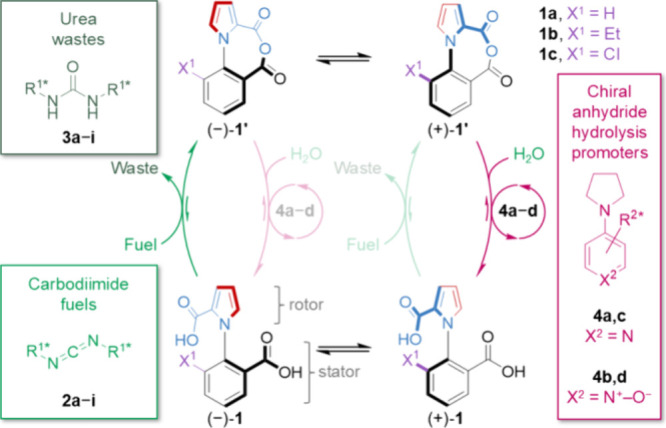
Chemomechanical cycle for the chemically fueled directional rotation of diacid motor-molecule **1**^2^. R^1^* and R^2^* = chiral substituents (see Figure 3 for R^1^* groups; see Figure 4 for R^2^* groups). Upon fueling with a chiral carbodiimide (**2a**–**i**, see Figure 3), the carboxylic acid groups of (−)-**1** react faster than (+)-**1** with one enantiomer of the fuel (Curtin–Hammett principle) to form the anhydride tether between the rotor and stator. Thus, (−)-**1′** is generated faster than (+)-**1′**. Rotamers (−)-**1′** and (+)-**1′** can rapidly exchange via a ring-flip. Hydrolysis of (+)-**1′** is promoted by one enantiomer of a chiral anhydride hydrolysis promoter (**4a**–**j**, see Figure 4) faster than the other (again, Curtin–Hammett principle), generating (+)-**1** faster. Diacid (+)-**1** can then convert back to (−)-**1** by rotation of the rotor CO_2_H group past the X^1^-substituent of the stator. For motor **1a** (X^1^ = H), passage of the CO_2_H group past the X^1^-substituent is fast. For motor **1b** (X^1^ = Et), passage of the CO_2_H group past the Et group is sterically blocked, allowing the ratio of atropisomers (−)-**1** and (+)-**1** to be measured by chiral HPLC. This allows directionality in the motor cycle to be determined. For motor **1c**, X^1^ = Cl, rotation is very slow at room temperature. However, at 90 °C, passage of the CO_2_H group past the Cl-substituent occurs on a time scale of a few hours. This enables the different steps in a single 360° rotation of the motor-molecule to be followed (Figure 7). Faded arrows represent pathways that are slower than the analogous step shown with arrows of normal intensity.

The autonomous chemically fueled directional rotation of aromatic rings around the C–N bond of 1-phenylpyrrole 2,2′-dicarboxylic acid, **1a**, was recently reported (Figure 1).^2^ Directional 360° rotation of the pyrrole rotor about the phenyl stator during the motor-molecule’s catalysis of carbodiimide hydration proceeds as follows (illustrated for clockwise-biased rotation; Figure 1): (i) Steric hindrance prevents the acid groups in (±)-**1a** from being able to pass each other. Enantioselective reaction of conformer (−)-**1a** with a chiral carbodiimide fuel forms an *O*-acyl urea intermediate, which is rapidly converted to anhydride (−)-**1′a** plus urea waste. (ii) A ring flip of the tethered anhydride now allows the carbonyl groups of (−)-**1′a** to pass each other, accessing the mirror image conformer (+)-**1′a**. (iii) Conformational selection through enantioselective nucleophilic attack of the chiral anhydride promoter (**4a**–**d**) into the anhydride of (+)-**1′a**, followed by hydrolysis of the resulting intermediate then generates (+)-**1a**. (iv) Rotation of the rotor acid of (+)-**1a** past the X-substituent of the stator reforms (−)-**1a**, completing the catalytic cycle.

The component rotation in **1a** occurs through two Curtin–Hammett-mediated dynamic kinetic resolutions^18^ during the motor’s catalysis^57−64^ of carbodiimide hydration (i.e., the fuel-to-waste reaction).^49^ Directionally biased rotation of the catalyst’s components continues as long as unspent carbodiimide fuel remains. The rotation can be directly followed^65^ for a single 360° rotary cycle using a derivative of the motor that undergoes full rotation only very slowly (**1c**; 1-(6′-chlorophenyl)pyrrole 2,2′-dicarboxylic acid). A derivative of **1a** has been used to power the twisting of the polymer strands in a gel around one another, resulting in contraction of the gel.^6^ This results in mechanical work being done at the molecular and macroscopic levels by the transduction of chemical energy by catalysis.^6^

Despite exhibiting a directional bias of only ∼3:1 (i.e., on average one backward rotation occurs for every three forward rotations), rotary motor **1a**^2^ (Figure 1) possesses a number of desirable features: (i) The motor is structurally simple, allowing its mechanism to be understood in some detail and to be accurately described by kinetic modeling. (ii) The motor is an efficacious catalyst for the fuel-to-waste reaction,^49^ with <3% fuel reacting through the background (i.e., uncatalyzed) reaction pathway under the original operating conditions. (iii) The direction of rotation of the motor is determined solely by the chirality of the fuel and anhydride hydrolysis promoter, and so rotation can be powered in either direction. (iv) The motor is doubly kinetically gated,^8,20^ i.e., the reaction rates of both the motor with the carbodiimide (anhydride formation), and the regeneration of the resting state of the motor (anhydride hydrolysis), depend on the mechanical state (i.e., conformation) of the motor. (v) Each of the enantioselective steps in the motor cycle proceed independently of each other. Since the stereochemistry of the carbodiimide fuel affects only anhydride formation, and the stereochemistry of the hydrolysis promoter affects only anhydride hydrolysis, the different reagents in the chemical fueling system can be independently optimized for the chemical step they take part in in the catalytic cycle.

**Figure 2:**
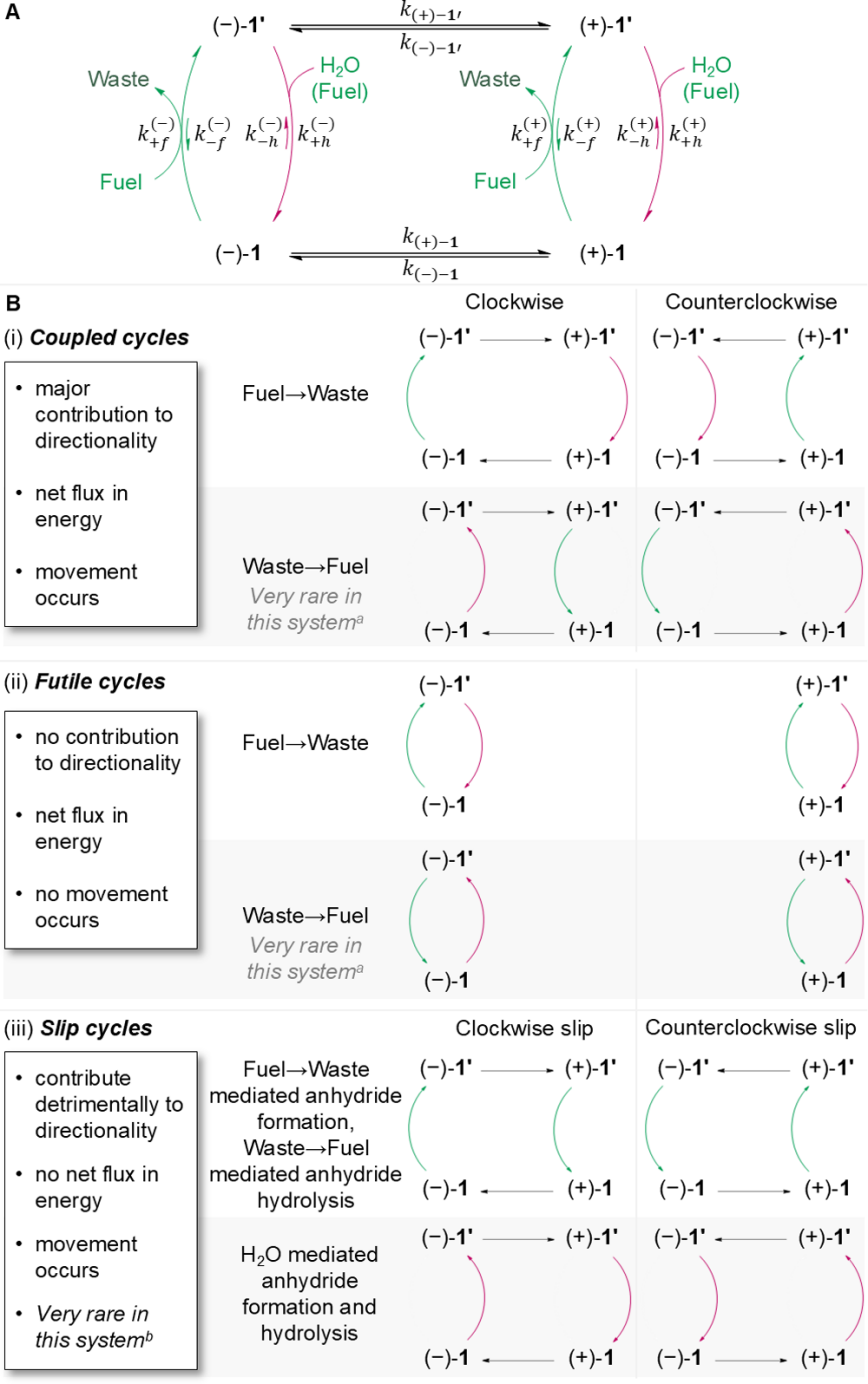
Reaction network (chemical engine cycle) of the fuel-driven rotation of motor **1**. (A) Scheme showing the various transformations involved in the operation of motor **1**, including the carbodiimide/fuel-to-urea/waste (green) and hydrolysis (red) reactions and their respective microscopic reverses, and the conformational exchange between the enantiomeric conformations of both the diacid **1** and anhydride **1′**. In the kinetic (*k*) descriptors, subscripts “*f*” and “*h*” denote anhydride formation and hydrolysis reactions, respectively, in the forward (+) or backward (−) pathways. Superscripts (−) and (+) indicate the axial stereochemistry of the conformations of diacid **1** and anhydride **1′**. (B) Various cyclic pathways through the chemical reaction network. ^a^Cycles involving the consumption of urea (waste) and the release of carbodiimide (fuel) are very rare in this motor system because of the large activation energy barrier for these chemical transformations to occur under the operation conditions. ^b^Slip cycles are also very rare in this motor system because of the large activation energy barrier for the acid groups to pass each other in the diacid form of the motor and because the tethering of the aryl rings prevents rear-side rotation in the anhydride form, **1′**.

**Figure 3:**
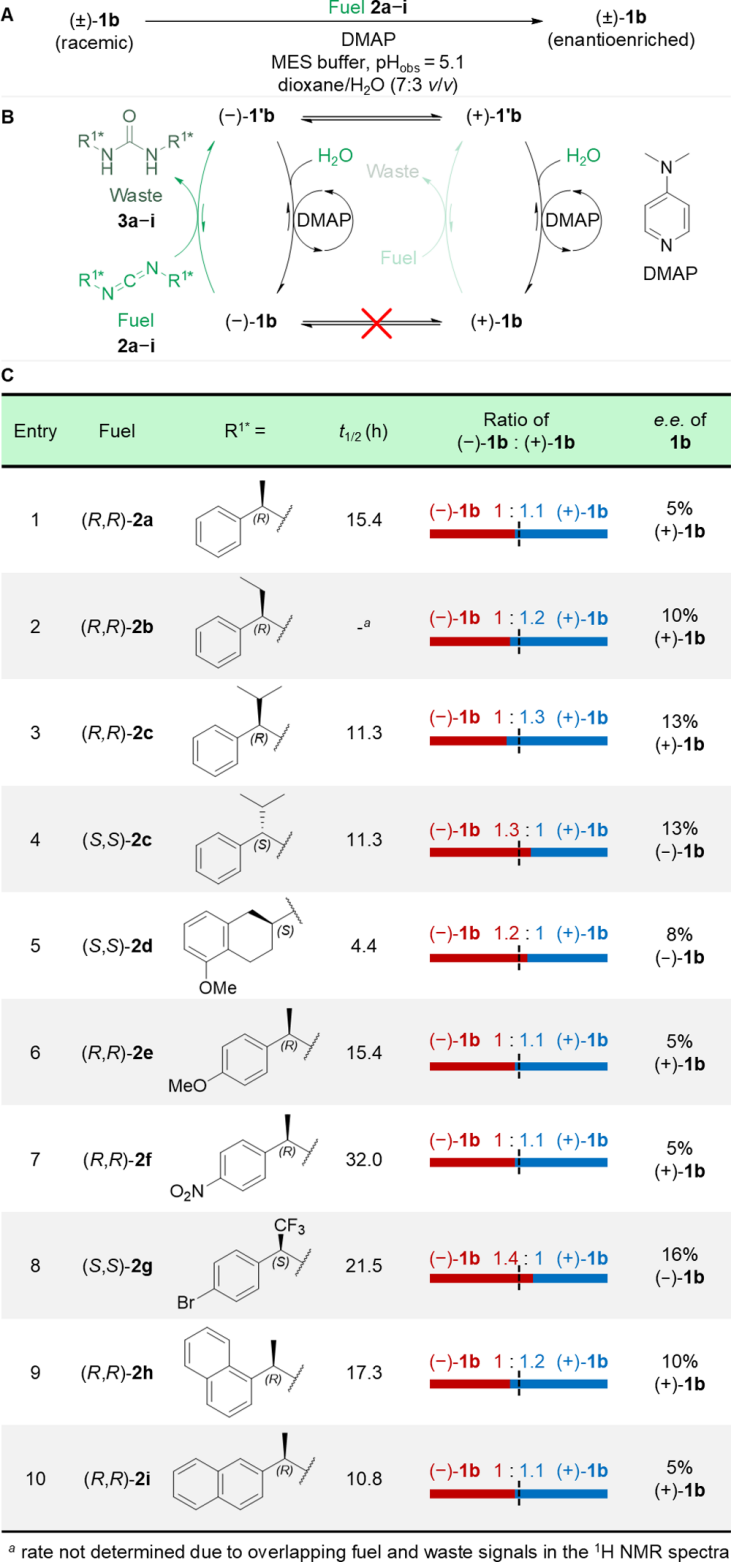
Effect of variation of the carbodiimide fuel structure on the directionality of model motor **1b**. (A) Racemic motor (±)-**1b** was fueled under a standard set of conditions ([motor **1**] = 1.0 mM, [fuel **2**] = 10.0 mM, [DMAP] = 1.0 mM, [MES buffer] = 100.0 mM, pH_obs_ = 5.1,^80^ dioxane/H_2_O (7:3 *v/v*), 10 °C, see the Supporting Information, Sections S3.1 and S4.2 for details) and the ratio of atropisomers following fuel consumption measured by chiral HPLC (ChiralPak IF column, 25 °C, CH_2_Cl_2_:*i-*PrOH:CF_3_CO_2_H:*n*-hexane, 66.5:3.4:0.1:30 *v/v/v/v*, 1 mL min^–1^, or *^i^*PrOH:CF_3_CO_2_H:*n*-hexane, 1.98:0.02:98 *v/v/v*, 2 mL min^–1^). (B) An achiral hydrolysis promoter (DMAP) ensures that the hydrolysis step does not contribute to the *e.e*.. This allows the measurement of the chemical gating of anhydride formation. (C) Table showing the structures of fuels **2a**–**i**, their half-lives of consumption in the reaction, and the measured directionalities, reported both as a ratio and an enantiomeric excess.

**Figure 4:**
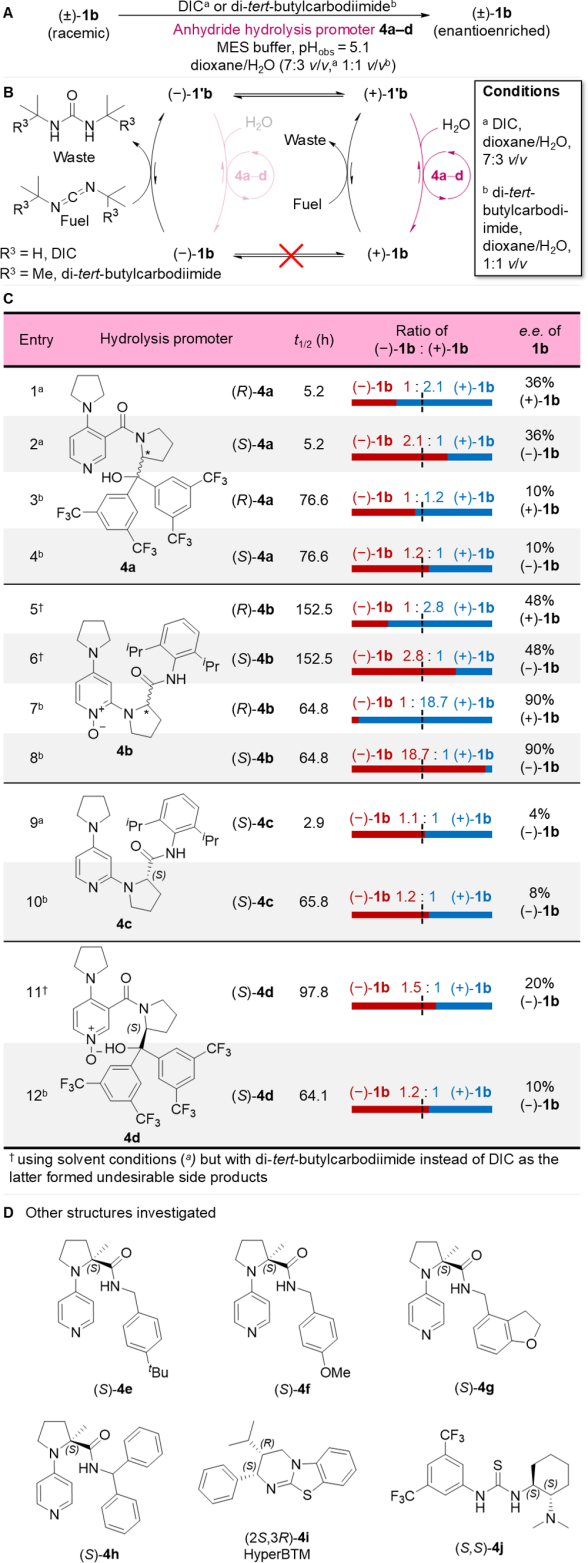
Effect of variation of the anhydride hydrolysis promoter on the directionality of model motor **1b**. (A) Racemic motor (±)-**1b** was fueled under a standard set of conditions (^a^[motor **1**] = 1.0 mM, [DIC] = 10.0 mM, [anhydride hydrolysis promoter **4a**–**d**] = 1.0 mM, [MES buffer] = 100.0 mM, pH_obs_ = 5.1,^80^ dioxane/H_2_O (7:3 *v/v*), 10 °C, or ^b^[motor **1**] = 1.0 mM, [di-*tert*-butylcarbodiimide] = 10.0 mM, [anhydride hydrolysis promoter **4a**–**d**] = 2.0 mM, [MES buffer] = 100.0 mM, pH_obs_ = 5.1,^80^ dioxane/H_2_O (1:1 *v/v*), rt, see the Supporting Information, Sections S3.2, S4.3, and S5 for details) and the ratio of atropisomers following fuel consumption measured by chiral HPLC as per Figure 3. (B) An achiral carbodiimide fuel (DIC or di-*tert*-butylcarbodiimide) was used, so that the anhydride formation step did not contribute to the *e.e.*. This experiment allows evaluation of the chemical gating of the anhydride hydrolysis step in isolation. (C) Table showing the structures of hydrolysis promoters **4a**–**d**, the half-life of the fuel used, and the measured directionalities, reported as both a ratio and an enantiomeric excess. (D) Other hydrolysis promoters investigated (**4e**–**j**), which displayed only very modest directionalities (see the Supporting Information, Section S4.3).

**Figure 5:**
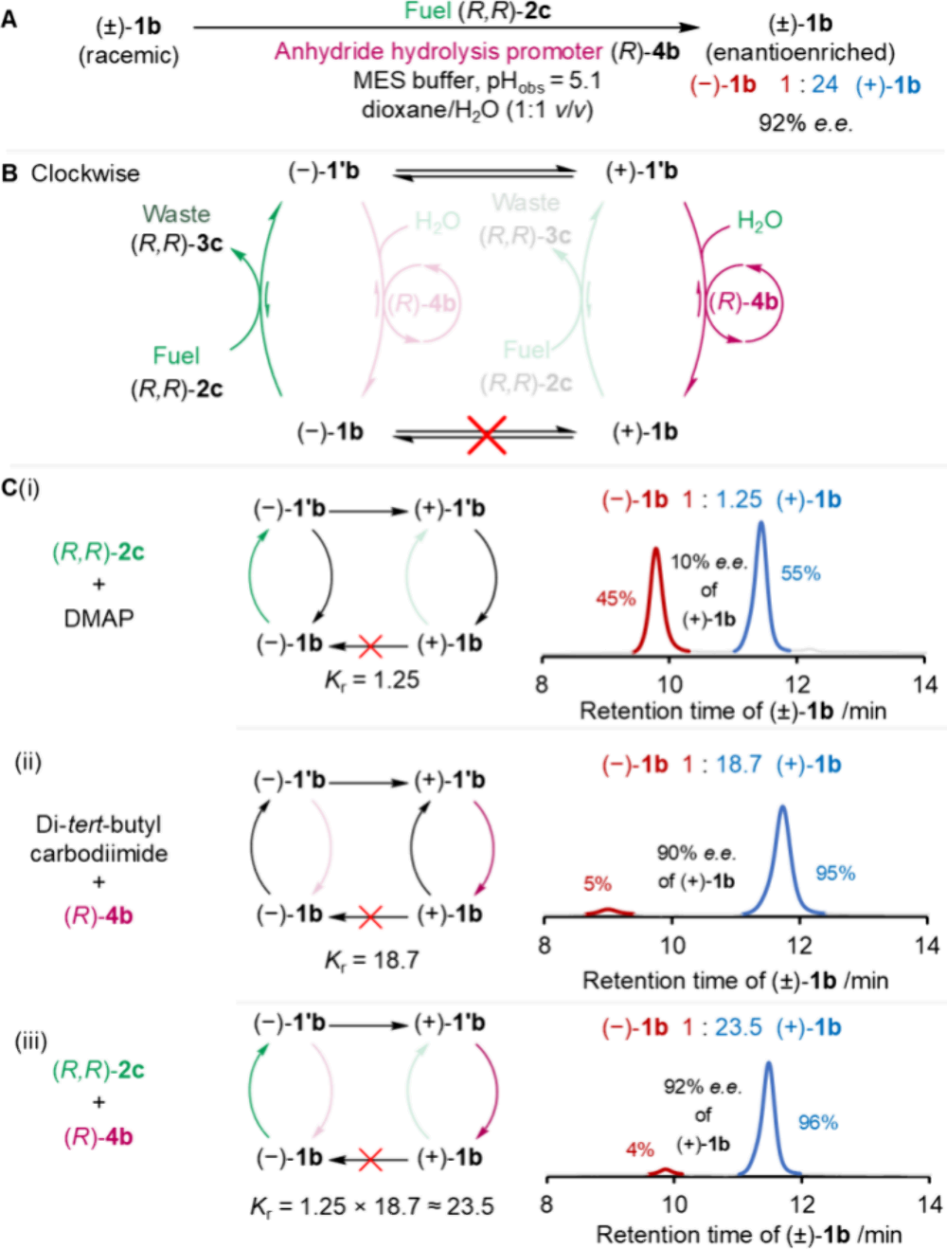
Double kinetic gating in the operation of model motor **1b**. (A) Racemic motor (±)-**1b** was fueled under reoptimized conditions ([motor **1b**] = 1.0 mM, [fuel] = 5.0 mM, [anhydride hydrolysis promoter] = 2.0 mM, [MES buffer] = 100.0 mM, pH_obs_ = 5.1,^80^ dioxane/H_2_O (1:1 *v/v*), r.t.). The ratio of (±)-**1b** after fueling was measured by chiral HPLC (ChiralPak IF column, 25 °C, *^i^*PrOH:CF_3_CO_2_H:*n*-hexane, 1.98:0.02:98 *v/v/v*, 2 mL min^–1^, see the Supporting Information, Section S6 for details). (B) The experiments allowed evaluation of resultant directionality when both chiral fuel (*R,R*)-**2c** and chiral hydrolysis promoter (*R*)-**4b** were used together. (C) Measured directionality directly corresponding to *K*_r_. In the singly kinetically gated experiments (i.e., using either chiral fuel or chiral promoter), the directionality also corresponds to the chemical gating of the individual step since there is no kinetic selection in the rest of the cycle. (i) Fueling with (*R,R*)-**2c** and DMAP under these conditions gave a directionality of 1:1.25 (10% *e.e.*), (ii) fueling with *N,N*-di-*tert*-butylcarbodiimide and (*R*)-**4b** gave a directionality of 1:18.7 (90% *e.e.*), and (iii) fueling with (*R,R*)-**2c** and (*R*)-**4b** gave a directionality of 1:24 (92% *e.e.*), corresponding to the product of the individual gatings.

**Figure 6:**
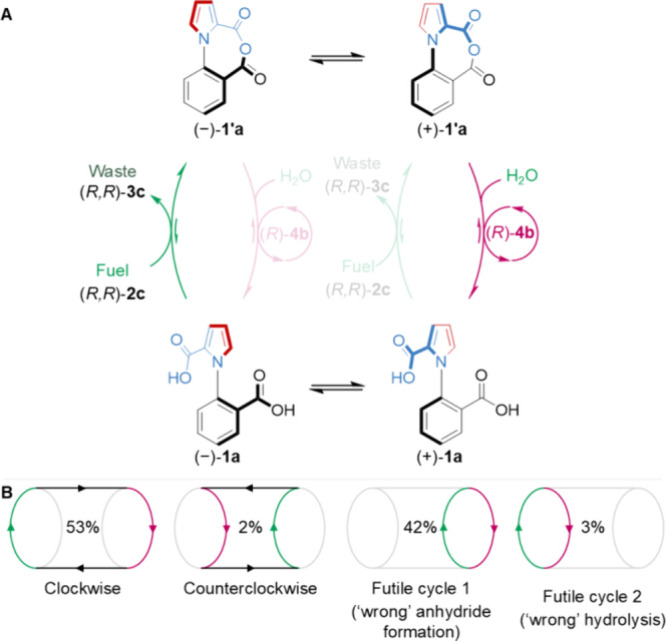
(A, B) Proportion of the catalyzed fuel-to-waste reaction proceeding via the coupled and futile cycles in the operation of motor **1a** under the reoptimized conditions shown in Figure 5 (see the Supporting Information, Section S9 for details). Slip cycles are negligible in this motor-molecule system (see Figure 2).

Below we discuss the chemical reaction network of motor **1** (Figure 2) and explore how structural variations of the chemical fuel (Figure 3) and the anhydride hydrolysis promoter (Figure 4) affect the directionality (Figure 5) and catalytic and coupling efficiency (Figure 6).^9,20^ This enables conclusions to be drawn regarding balancing the rate of catalysis with the directionality of rotation. The former correlates with motor speed and the fraction of the fuel that is productively used to drive the motor, while the latter is the product of the two kinetic gating values. We find that improved directionality occurs at the expense of slower motor speeds. We discuss the design principles that these results suggest might prove desirable and efficacious for subsequent generations of artificial chemically fueled motor-molecules.

## Results and Discussion

### Chemical Reaction Network of Motor-Molecule **1**

We can identify several important performance characteristics of molecular motors: (i) Directionality (i.e., ratio of forward cycles to backward cycles); (ii) speed (i.e., net forward cycles per unit time); (iii) coupling efficiency (i.e., net forward rotations per equivalent of fuel consumed via machine-catalyzed pathways); and (iv) fuel efficiency (i.e., net forward rotations per equivalent of supplied fuel).

The fuel efficiency of an autonomous chemically fueled motor is dependent both on the motor’s efficacy as a catalyst (catalytic efficiency, which is the proportion of the supplied fuel that is consumed via motor-catalyzed pathways) and the efficiency of the coupling of fuel consumption to the directional movement (coupling efficiency, see above). Motor speed is also contingent on the efficiency of the coupling, along with the average rate of fuel consumption by the motor (see the Supporting Information, Section S10).

Directionality is the principal requirement for any molecular motor.^9,20^ While other properties, such as speed and efficiency, may be important in particular contexts,^9,56−72^ fundamentally a linear motor should move forward not backward, and a rotary motor should turn in one direction rather than the other. We note that the directionality of molecular motors is governed by statistical thermodynamics, and thus will always necessarily involve a certain proportion of “backward” rotations. Directional motion in an information ratchet-driven molecular motor is a direct consequence of the kinetic asymmetry^17,20,73^ in the machine’s chemical engine cycle (Figure 1).^11−20,49,73^ For motor **1**^2^, kinetic asymmetry arises solely through chemical gating—that is the kinetically controlled enantioselectivity of anhydride formation (Figure 1, dark green vs light green) and anhydride hydrolysis (Figure 1, dark pink vs light pink).^20,49^ The kinetic asymmetry results in a kinetic preference for following specific cycles of reactions within the chemical reaction network, imparting directionality to the system as a whole when driven by energy dissipation from the catalyzed fuel-to-waste reaction.^20,73^

To visualize how directionality arises, it is helpful to deconstruct the chemical reaction network of motor **1** into different cyclic routes through the network (Figure 2).^74^ These cycles can be classified into three categories,^17,20^ which have the potential to affect the overall directionality and efficiency of the molecular motor in different ways (Figure 2B):

i. Coupled cycles (Figure 2B(i)) are the productive pathways for chemical engines since they couple the conversion of fuel to waste with the directional movement of the motor, enabling energy dissipation from the fuel-to-waste reaction to drive directional motion. Through these cycles, motor-molecule **1** catalyzes carbodiimide hydration and performs a directional rotation. Coupled cycles can be clockwise or counterclockwise, contributing positively or negatively to directionality. As a consequence of microscopic reversibility,^75^ reverse cycles involving the conversion of waste-to-fuel must also be considered in this category. However, in the case of these carbodiimide hydration driven motors, the high chemical potential of the fuel-to-waste reaction biases the fuel-to-waste reaction so heavily in favor of the waste as to make waste-to-fuel processes vanishingly rare.
ii. Futile cycles (Figure 2B(ii)) consume fuel without net movement occurring, thus dissipating the energy released from the motor-catalyzed fuel-to-waste reaction without transduction to the mechanical degree of freedom of the motor.^11^ In this process, the motor remains in one side (i.e., left or right) of the chemical reaction network shown in Figure 2A, cycling between (−)-**1** and (−)-**1′**, or (+)-**1** and (+)-**1′**, while consuming fuel. Futile cycles do not contribute to directionality (since they involve no net movement), but due to fuel consumption they decrease the coupling efficiency of the motor (i.e., the fraction of forward rotations that occur for each equivalent of fuel converted to waste). As with coupled cycles, reverse cycles involving waste-to-fuel conversion are also included in this category.^19^
iii. Slip cycles (Figure 2B(iii)) involve movement without net consumption of fuel. Either the fuel is both formed and consumed within the slip cycle, or the motor rotates without involving any chemical steps (e.g., the acid groups slip past each other). Slip cycles do not involve a net exchange of energy with the environment and so, as a consequence of microscopic reversibility, slip cycle pathways necessarily occur with the same frequency in both the clockwise and counterclockwise directions (in the absence of an applied force). As they are inherently nondirectional, the presence of slip cycles decrease directionality by competing with the number of coupled cycles.^76^ As they do not consume fuel, slip cycles do not affect motor efficiency. Slip cycles are vanishingly rare for motor **1** as there is a large activation energy barrier for the acid groups to slip past each other in the diacid state of the motor, and the acyl groups cannot pass each other, other than through ring-flipping, in the tethered (anhydride) state of the motor (**1′**).

For motor **1**^2^, both chemical steps (carbodiimide-induced anhydride formation and anhydride hydrolysis) are highly exergonic under the reaction conditions,^77,78^ making all of the cycles featuring the microscopic reverse of either step (i.e., reacting urea with anhydride to form a carbodiimide or spontaneous dehydration of the diacid motor to form an anhydride) extremely rare. Therefore, the only substantial factors affecting directionality and efficiency in this motor system are the ratio between the dissipative clockwise and counterclockwise coupled cycles (i.e., kinetic asymmetry), and the frequency of futile cycles compared to coupled cycles.^20^

For clockwise rotation, the desired clockwise coupled cycles can be favored by improving the degree of chemical gating, which increases kinetic asymmetry.^20^ Chemical gating of anhydride formation depends only on the Curtin–Hammett discrimination of the enantiomeric conformations of the diacid form of the motor with the chiral carbodiimide.^2^ Likewise, gating of anhydride hydrolysis depends only on the Curtin–Hammett discrimination of the enantiomeric conformations of the anhydride form of the motor with the chiral hydrolysis promoter.^2^ Consequently, variations in the structure of the fuel and the hydrolysis promoter can be explored independently, assessing how they affect directionality and other aspects of motor performance. Since the two chemical gatings combine multiplicatively to generate the overall kinetic asymmetry,^8,15,17,20,49^ even small improvements in the gating of these individual steps can result in a substantial improvement in the overall directionality of the motor. The following Sections describe the effects of varying the structure of the carbodiimide fuel and the anhydride hydrolysis promoter on the performance characteristics of motor operation using derivative **1b** of the motor-molecule where atropisomer isomerization is blocked by an Et group.^2^

### Varying the Carbodiimide Fuel Structure

The influence of the carbodiimide fuel structure on the kinetic gating of anhydride formation was probed independent of the kinetic gating of the anhydride hydrolysis step by using an achiral hydrolysis promoter, 4-dimethylaminopyridine (DMAP). Under our standard fueling conditions, where anhydride hydrolysis is fast with respect to anhydride formation,^79^ fueling a model diacid (**1b**) with an excess of the chiral carbodiimide led to a constant enantiomeric excess (*e.e*.) of the diacid at the chemically fueled steady state (Figure 3; see the Supporting Information, Sections S3.1 and S6.3). The originally reported conditions used to operate **1b** with DMAP and fuel **2a** resulted in a modest enantioselectivity of 1:1.1 (5% enantiomeric excess (*e.e.*)).^2^ We synthesized a series of other chiral carbodiimides (**2b**–**i**, see the Supporting Information, Section S2.2) to explore the influence of carbodiimide structure on this kinetically gated step. The carbodiimide fuels vary in the sterics of the aliphatic component (**2a**–**c**), aromatic substituents (**2a**,**h**,**i**) and other stereoelectronic characteristics (**2d**–**g**).

Model motor **1b** was fueled using the previously established standard operating conditions^2^ with fuels **2a**–**i** in the presence of DMAP as the achiral anhydride hydrolysis promoter ([motor **1b**] = 1.0 mM, [fuel **2**] = 10.0 mM, [DMAP] = 1.0 mM, [2-(*N*-morpholino)ethanesulfonic acid (MES) buffer] = 100.0 mM, pH_obs_ = 5.1,^80^ dioxane/H_2_O (7:3 *v/v*), 10 °C), see the Supporting Information, Sections S3.1 and S4.2 for details). The results are shown in Figure 3.

Notable differences in the *e.e.* values of **1b** (Figure 3) arise from the use of fuels **2a**–**c**, which differ in the steric bulk of the alkyl groups. The isopropyl groups in **2c** result in a directionality of 1:1.3 (13% *e.e.*), compared to 1:1.2 (10% *e.e.*) and 1:1.1 (5% *e.e.*) for the less-bulky ethyl (**2b**) and methyl groups (**2a**), respectively. The bulkier fuel, **2c**, also shows a slightly faster rate of fuel consumption (*t*_1/2, **2c**_ = 11.3 h *cf. t*_1/2, **2a**_ = 15.4 h). The basicity of the carbodiimide is enhanced by the higher degree of substitution, accelerating its hydration under acidic pH.^81^

Restricting the conformational freedom around the asymmetric center in fuel **2d** had little effect of the directionality (1.2:1; 8% *e.e.*), but increased the rate of fuel consumption (i.e., catalysis) by the motor (*t*_1/2, **2d**_ = 4.4 h) over background, leading to enhanced catalytic efficiency (95%, see the Supporting Information, Section S3.1). Increasing the size of the aryl group (fuels **2h** and **2i**, cf. fuel **2a**) had little effect on directionality or rate of fuel use (1:1.2 with *t*_1/2, **2h**_ = 17.3 h for **2h**; 1:1.1 with *t*_1/2, **2i**_ = 10.8 h for **2i**; 1:1.1 with *t*_1/2, **2a**_ = 15.4 h for **2a**). Varying the electronic nature of the aromatic ring from electron-rich (**2e**) to electron-poor (**2f**), also had little effect on directionality. Replacing the alkyl at the asymmetric center with a CF_3_ group (**2g**), resulted in a small increase in directionality (1.4:1; 16% *e.e.*) at the expense of lower catalytic efficiency (73%, see the Supporting Information, Section S3.1). In addition to assessing the directionality induced by the different fuels, we also determined the kinetics of the motor-catalyzed fuel-to-waste reaction. This characterizes how efficiently the motor can use the fuel as well as the speed of motor rotation.^9^ Under the standard operating conditions,^2^ all of the fuels except **2g** showed similar catalytic efficiencies, with 85–98% of fuel-to-waste reactions proceeding by the machine-catalyzed pathway. Carbodiimide **2g** had a high background rate of hydration under the operating conditions, leaving a relatively small proportion of fuel molecules to react through the motor-catalyzed pathway (Supporting Information, Section S3.1). Overall, carbodiimide **2c** provides a useful balance of directionality, speed and efficiency under the operating conditions.

### Varying the Anhydride Hydrolysis Promoter Structure

Chemical gating^20^ of the anhydride hydrolysis step results from enantioselective hydrolysis of the rapidly exchanging mirror-image conformations of the anhydride form of the motor ((−)-**1′**⇋(+)-**1′**). This dynamic kinetic resolution^18^ requires a chiral catalyst for anhydride hydrolysis. We will refer to this additive as a “hydrolysis promoter”, rather than as a catalyst, so as to not cause confusion with the overall role of the motor-molecule in catalyzing the transformation of carbodiimide plus water to urea. The hydrolysis promoter needs to differentiate between the atropisomeric conformations of the anhydride, and also outcompete both direct aqueous hydrolysis of the anhydride at pH 5.1 and intermolecular anhydride formation, which can result in the buildup of motor oligomers.^2^

The influence of the structure of the hydrolysis promoter on the kinetic gating of anhydride hydrolysis could be determined independent of the kinetic gating of the anhydride formation step by using an achiral carbodiimide fuel, such as *N,N*-diisopropylcarbodiimide (DIC) or *N,N-*di-*tert*-butylcarbodiimide. We evaluated potential chiral hydrolysis promoters based on 3-substituted- and 2-substituted-4-pyrrolidinopyridines (**4a**^82^ and **4c**,^83^Figure 4C) and their corresponding *N*-oxides (**4d** and **4b**,^83^Figure 4C). Several other chiral 4-pyrrolidinopyridine derivatives (**4e**–**h**,^84^Figure 4D), isothiourea **4i**(85) (Hyper BTM, Figure 4D), thiourea **4j**(86) (Takemoto’s catalyst, Figure 4D) and an additional DMAP-derivative^87^ and a DMAP-*N*-oxide^88^ developed by the Spivey group were also investigated (see Section S4.3 for a full list of hydrolysis promoters investigated).

Model motor **1b** was fueled under the standard conditions used for the chiral carbodiimide fuels in Figure 3, but this time using achiral *N,N*-diisopropylcarbodiimide (DIC) in the presence of anhydride hydrolysis promoters **4a**–**j** ([**1b**] = 1.0 mM, [DIC] = 10.0 mM, [**4a**–**j**] = 1.0 mM, [MES-buffer] = 100.0 mM, pH_obs_ = 5.1,^80^ dioxane/H_2_O (7:3 *v/v*), 10 °C). The results are shown in Figure 4 (Supporting Information, Sections S3.2, S4.3, and S5). Of the 4-pyrrolidinopyridine anhydride hydrolysis promoters (**4a,c,e**–**j**), **4a** produced the highest degree of deracemization (36% *e.e.*; Figure 4C, entries 1 and 2) of **1b** under fueling with DIC. *N*-Oxide **4b** generates a higher *e.e.* (48% *e.e*.; Figure 4C, entries 5 and 6) but at the expense of much slower reaction times (albeit using a bulkier carbodiimide fuel, di-*tert*-butylcarbodiimide). In all cases, use of the enantiomeric hydrolysis promoter generated an equal and opposite *e.e.* in **1b**.

The operation of **1b** with *N*-oxide **4b** and DIC resulted in the accumulation of the *N*-acyl urea of **1b** due to relatively slow hydrolysis of the motor–promoter *N*-oxide-ester (Supporting Information, Sections S5.1 and S8). While one acid group of the motor is temporarily blocked as the *N*-oxide-ester, any *O*-acyl urea formed by reaction of the other motor carboxylic acid group with DIC, will tend to rearrange to the *N*-acyl urea, instead of forming the intramolecular anhydride.

The issue with *N*-acyl urea formation with **4b** could be substantially reduced in a number ways: (i) Increasing the steric bulk of the carbodiimide fuel (replacing DIC with *N,N*-di-*tert*-butylcarbodiimide) (see the Supporting Information, Section S5.1). However, although this reduces the rate of rearrangement to the *N*-acyl urea, it also results in a slower rate of the motor-catalyzed fuel-to-waste reaction (fuel *t*_1/2_ = 76.6 h). (ii) Alternatively, the carbodiimide fuel could be kept constant at low concentration (≤1 mM) by continuous syringe pump addition, allowing time for full hydrolysis of the motor–promoter adduct (Supporting Information, Section S6.3). (iii) 1-Hydroxybenzotriazole (HOBt) could be added to quickly form an OBt ester from the *O*-acyl urea before the *O*-acyl → *N*-acyl urea rearrangement occurs, allowing enough time for the *N*-oxide ester to fully hydrolyze (see the Supporting Information, Section S8.2).

Using bulky di-*tert*-butylcarbodiimide in place of DIC in the presence of hydrolysis promoter **4b**, **1b** was deracemized in 48% *e.e.* (Figure 4C, entries 5 and 6), without any *N*-acyl urea being detected. No oligomeric anhydrides were observed under these conditions, a minor side-reaction which occurs under the original motor-operating conditions^2^ (Supporting Information, Section S5). Similarly, **4c**, the 4-pyrrolidinopyridine analog of *N*-oxide **4b**, generated 4% *e.e.* of **1b** (using DIC; Figure 4C, entry 9), likely a consequence of the poor nucleophilicity of *ortho-*substituted pyridines.^89^ Compound **4d**, the *N*-oxide analog of 4-pyrrolidinopyridine **4a**, also generated modest deracemization of **1b** (20% *e.e.* with *N,N*-di-*tert*-butylcarbodiimide; Figure 4C, entries 11).

### Optimization of Motor Fueling Conditions with Hydrolysis Promoter **4b**

Since *N*-oxide **4b** generates the highest enantioselectivity of deracemization of **1b**, we reoptimized the fueling conditions for this hydrolysis promoter with a view to further increasing the directionality of motor operation (Supporting Information, Section S5). These optimized conditions feature an increase in both the proportion of water and the concentration of the hydrolysis promoter: [**1b**] = 1.0 mM, [*N,N*-di-*tert*-butylcarbodiimide] = 10.0 mM, [**4b**] = 2.0 mM, [MES buffer] = 100.0 mM, pH_obs_ = 5.1,^80^ dioxane-*d*_8_/D_2_O (1:1 *v/v*), r.t. Under these conditions, the deracemization of **1b** rose to 90% *e.e.* under fueling with *N,N*-di-*tert*-butylcarbodiimide with either handedness of **4b** (Figure 4C, entries 7 and 8). However, fueling with anhydride hydrolysis promoters **4a**, **4c** or **4d** under the reoptimized conditions did not result in significant increases in *e.e.* (Figure 4C, entries 3, 4, 10, 12), and gave worse directionality with **4d**.

### Double Kinetic Gating of Motor Model **1b** with Optimized Fuel and Hydrolysis Promoter

Having identified *N,N*-di(isopropylbenzyl)carbodiimide **2c** and *N*-oxide anhydride hydrolysis promoter **4b** as the chiral reagents that give the highest directionality in the individual chemical gating steps, we combined these in a fueling system that drives motor **1** through double kinetic gating (Figure 5).^8,20^ Due to the excellent enantioselectivity for deracemization achieved with **4b** under the reoptimized motor-operating conditions, we used these conditions for the double kinetic gating experiments. Under these conditions the enantioselectivity of the anhydride-forming step with fuel **2c** and DMAP decreased slightly to 10% *e.e.* ([**1b**] = 1.0 mM, [**2c**] = 10.0 mM, [DMAP] = 2.0 mM, [MES buffer] = 100.0 mM, pH_obs_ = 5.1,^80^ dioxane-*d*_8_/D_2_O (1:1 *v/v*), r.t., Supporting Information, Section S6.1).

The directionality obtained from the individual kinetic gatings of the fueling and hydrolysis steps should, in principle, combine multiplicatively to give a directionality of ∼24:1 (1.3 × 18.7 = 24.3) for doubly kinetically gated operation.^20^ However, fueling **1b** in the presence of **4b** with 10 equiv of **2c** added all-at-once at time zero, resulted in appreciable *N*-acyl urea accumulation. This is likely a consequence of the slow rate of hydrolysis of the intermediate motor-ester of the pyridine *N*-oxide (Supporting Information, Section S5, S6.2, and S8). To overcome this issue, we continuously fueled **1b** with a low concentration of **2c** by syringe pump addition throughout the course of motor operation (Supporting Information, Section S6.3). This is reminiscent of the on-demand availability of ATP in cells, where low ATP concentrations (<10 μM) are maintained for motor operation.^90^ Maintaining a low fuel concentration in this way reduced the unwanted side reactions, resulting in a 92% *e.e.* of (+)-**1b** which was maintained over the course of 90 h (Figure 5C(iii)), matching the directionality predicted from the individual gating experiments (Figure 5C(i,ii)). From the rate of fuel consumption and the individual gatings, under the chemostated operating conditions **1b** completes a rotational catalytic cycle every ∼40 h, one backward rotation for every 24 forward rotations in both clockwise and counterclockwise directions using chirality-matched fuel and hydrolysis promoter, with >99% of the fuel-to-waste reactions proceeding through the catalyzed pathway (see the Supporting Information, Section S10).

Since mechanical exchange is fast and unbiased in motor **1a**, the contributions of each cycle for fuel-to-waste conversion should be similar to the chemical gating for each step measured for **1b** (Supporting Information, Section S9). The close correlation between the rates of anhydride formation and hydrolysis in the catalytic cycles of **1a** and **1b** is apparent from their very similar rates of catalysis.^2^ Applying the individual rates measured for **1b** to 360° rotation of **1a** indicates that ∼51% of motor-catalyzed fuel-to-waste reactions result in net directional rotation (53% forward −2% backward; Figure 6 and Supporting Information, Section S9).

### Single 360° Directionally Biased Rotation of Motor **1c**

**Figure 7:**
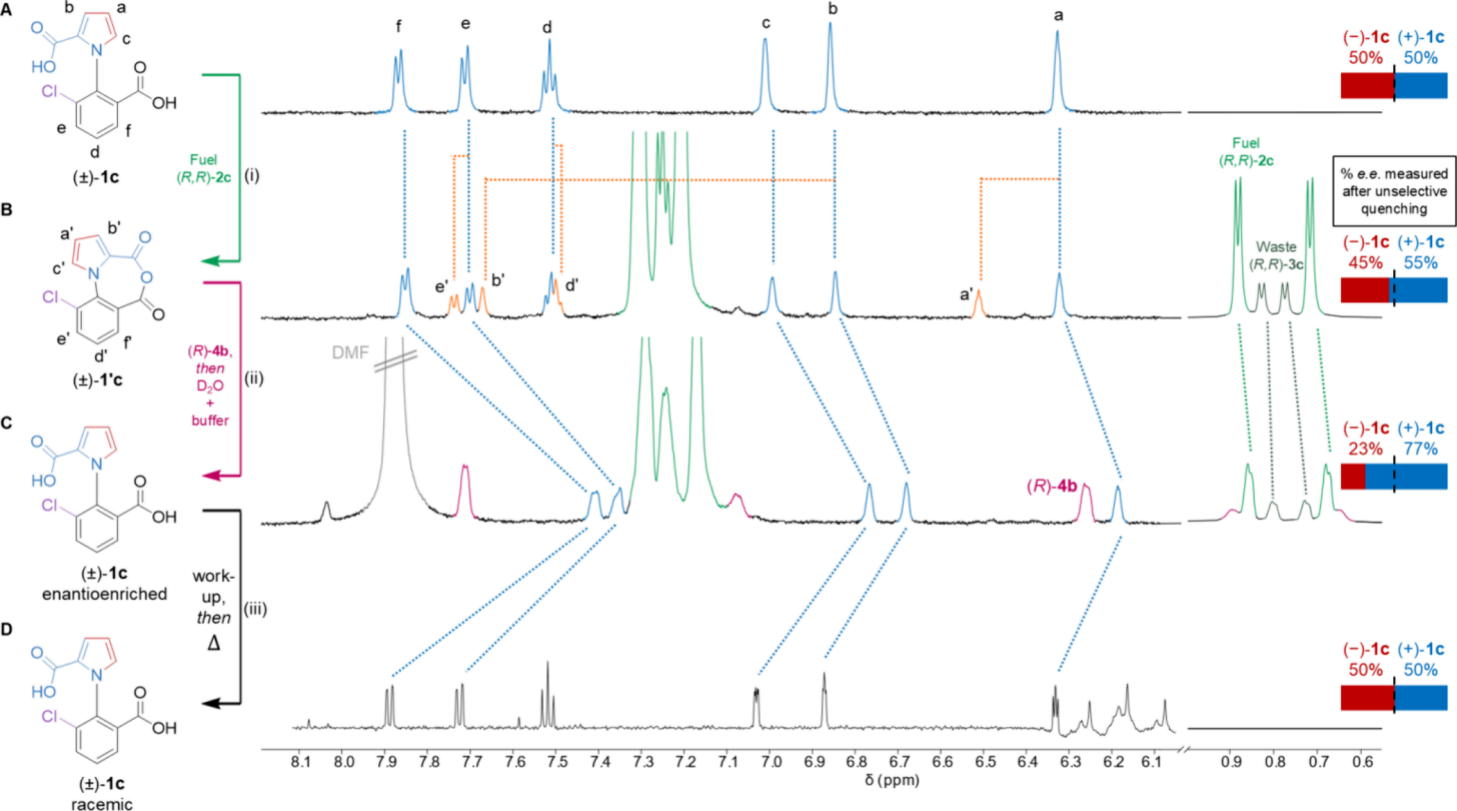
Stepwise 360° directionally biased rotation of the pyrrole rotor around the phenyl stator of **1c**. Partial ^1^H NMR spectra (CD_3_CN or CD_3_CN:D_2_O (1:1 *v/v*), 600 MHz, 298 K) showing the stepwise formation and hydrolysis of anhydride **1′c**. The region 6.0–8.2 ppm is scaled vertically 15× compared to region 0.5–1.0 ppm. (A) Racemic motor (±)-**1c** (1 equiv, 1.0 mM), in CD_3_CN. (B) 5 min after addition of fuel (*R,R*)-**2c** ((i), 2 equiv, 2.0 mM), in CD_3_CN. Quenching a similar sample with basic buffer solution and DMAP-mediated unselective hydrolysis afforded 10% *e.e.* of (+)-**1c** ([DMAP] = 2.0 mM, [MOPS buffer] = 100.0 mM, pH_obs_ = 7.9, D_2_O 50% *v/v*). (C) Three days after addition of (*R*)-**4b** to selectively ring-open **1′c** directly followed by basic buffer to complete the hydrolysis step yielded motor **1c** in 54% *e.e.*. ((ii), [**4b**] = 2.0 mM, [MOPS buffer] = 100.0 mM, pH_obs_ = 7.9, CD_3_CN:D_2_O (1:1 *v/v*)). The DMF peak originates from the stock solution of (*R*)-**4b**. (D) Heating motor-molecule **1c** after isolation for 22 h at 90 °C led to complete racemization (the gas phase barrier to rotation of **1c** was previously calculated^2^ to be 114 kJ mol^–1^ at 293 K), (iii), completing one full 360° directionally biased rotation of the pyrrole rotor about the phenyl stator. The ratio of (±)-**1c** after fueling was measured by chiral HPLC (ChiralPak IF column, 25 °C, *^i^*PrOH:CF_3_CO_2_H:*n*-hexane, 1.98:0.02:98 *v/v/v*, 2 mL min^–1^, see the Supporting Information, Section S7 for details).

Complete 360° about the C–N bond in motor-molecule **1c** (1-(6′-chlorophenyl)pyrrole 2,2′-dicarboxylic acid) is very slow at room temperature as passage of the pyrrole carboxylic acid group past the Cl-substituent is sterically hindered.^2^ The resulting slow interconversion of atropisomers (−)-**1c** and (+)-**1c** enables the enantioenrichement in the diacids to be followed stepwise for a single 360° rotation of the rotor about the stator (Figure 7).

Treatment of the racemic motor-molecule (±)-**1c** (Figure 7A) with (*R,R*)-**2c** formed anhydride **1′c** (Figure 7B(i)). Quenching the stepwise motor operation at about 30% conversion of **1c** to **1′c** produced 10% *e.e.* of (+)-**1c**, showing that (−)-**1c** had reacted faster with (*R,R*)-**2c** than (+)-**1c** (see the Supporting Information, Section S7). A similar stepwise fueling of **1c** was carried out and quenched at about the same conversion to **1′c**, this time with hydrolysis promoter (*R*)-**4b**, (ii), followed immediately by a basic buffer solution (MOPS buffer, pH_obs_ = 7.9) that stops motor-catalyzed carbodiimide hydration.^64^ Complete hydrolysis of the anhydride afforded (+)-**1c** in 54% *e.e.* (Figure 7C(ii)), demonstrating additional directional bias had been provided by anhydride hydrolysis. Heating the enantioenriched motor after isolation led to racemization (see the Supporting Information, Section S7 for details). As the acid groups cannot pass each other, this racemization step must occur by the rotor carboxylic acid group passing over the chlorine substituent of the stator, completing a single 360° directionally biased rotation of the components.

## Conclusions

The combination of chiral carbodiimide **2c** and chirality-matched hydrolysis promoter **4b** drives directional rotation of a 1-phenylpyrrole 2,2′-dicarboxylic acid rotary molecular motor (**1a**) through a doubly kinetically gated catalytic cycle,^8,20^ with a directionality of ∼24:1, a substantial increase over the ∼3:1 directionality obtained with the original fueling system.^2^ Under these conditions >99% (up from 97% obtained with the original fueling system) of the fuel reacts through motor-catalyzed pathways and >50% (up from 14%) of the fuel-to-waste reactions result in directional rotation of the motor components. However, attaining such high directionality and efficiency requires a slow rate of fuel addition (because *N*-oxide esters are formed at both carboxylic acid sites at high carbodiimide concentrations, blocking anhydride formation and leading to *N*-acyl urea formation, not because there is any change in kinetic asymmetry). The slow rate of fuel addition required means a modest speed of rotation (each catalytic cycle takes ∼40 h under these conditions), reflecting the sort of trade-offs in different aspects of performance that are common to both macro-scale motors^91^ and biomolecular machinery.^66−72,92^

The mechanism and performance indicators for how this minimalist catalysis-driven molecular motor works are closely related to the transduction of chemical energy through motor-catalysis in biology. Identifying key performance characteristics for such motors, and understanding how different pathways within the catalyst reaction network influence efficiency and directionality, should aid the design of artificial chemically fueled molecular motors that perform useful tasks.^6^

## Supporting Information Available

Experimental and operation procedures, synthesis, characterization data, NMR, mass spectrometry, and HPLC data (PDF)

## ABBREVIATIONS

HPLC: high-performance liquid chromatography
NMR: nuclear magnetic resonance.
