# Supplementary material for: Structural Influence of the Chemical Fueling System on a Catalysis-Driven Rotary Molecular Motor
Source: J Am Chem Soc. 2025 Feb 27;147(10):8785–95. doi: 10.1021/jacs.5c00028 (PMC11912321; doi:10.1021/jacs.5c00028)
Supplement: Supplementary file 1 — ja5c00028_si_001.pdf [file ja5c00028_si_001.pdf]

Structural influence of the chemical fueling system on a catalysis-driven rotary molecular motor

Hua-Kui Liu,[1‡] Toufic W. Mrad,[1‡] Axel Troncossi,[1‡] Stefan Borsley,[1] Benjamin M. W. Roberts,[1] Alexander Betts,[1] David A. Leigh[1, 2*]

[1]Department of Chemistry, University of Manchester, Manchester M13 9PL, UK.

[2]School of Chemistry and Molecular Engineering, East China Normal University, Shanghai, China

*Correspondence to: david.leigh@manchester.ac.uk

Supporting Information

Table of Contents

S1 General Information S3

S2 Synthesis and Characterization of Compounds S5

S2.1 Synthesis and characterization of motor compounds **1a–c** S5

S2.2 Synthesis and characterization of carbodiimide fuels **2a–i** S7

S2.3 Synthesis and characterization of hydrolysis promoters **4a–h** S16

S3 Monitoring fuel consumption during autonomous motor operation using ^{1}H NMR S21

S3.1 Monitoring the consumption of DIC and fuels **2a–i** S21

S3.2 Monitoring fuel consumption in the presence of hydrolysis promoters **4b–d** S31

S4 Single kinetic gating analysis S33

S4.1 Assignment of atropisomers of (±)-**1b** to HPLC peaks S34

S4.2 Enantioenrichment using fuels **2a–i** S35

S4.3 Enantioenrichment using hydrolysis promoters **4a–j** S37

S4.4 Methods to measure kinetic gating S39

S4.4.1 Gating of the anhydride hydrolysis step S39

S4.4.2 Gating of the anhydride formation step S39

S5 Optimization of the hydrolysis kinetic gating S45

S5.1 Optimizations for hydrolysis promoter **4b** S45

S5.2 Enantioenrichment using hydrolysis promoters **4a–d** under the re-optimized conditions S48

S5.3 Monitoring fuel consumption in the presence of hydrolysis promoters **4a–d** under the re-optimized conditions S50

S6 Double kinetic gating analysis S52

S6.1 Kinetic gating of **2c** under the re-optimized conditions of **4b** S52

S6.2 Batch-fueling experiment S53

S6.3 Chemostating experiment S54

S7 Completing 360° rotation using motor–molecule **1c** S57

S8 Mitigating *N*-acyl urea formation S62

S8.1 Mechanism of degradation of motor **1** S62

S8.2 Using HOBt as a protecting agent S65

S9 Probabilities of the different chemomechanical cycles S68

S10 Estimating the rotation rate of the motor at steady-state S70

S11 NMR Spectra of new compounds S72

S12 References S99

S1. General Information

Unless stated otherwise, reagents were obtained from commercial sources and used without purification. Anhydrous solvents were obtained by passing the solvent through an activated alumina column on a Phoenix SDS (solvent drying system; JC Meyer Solvent Systems, CA, USA). ^{1}H NMR and ^{13}C NMR spectra were recorded on a Bruker Avance III instrument with an Oxford AS600 magnet equipped with a cryoprobe [5 mm CPDCH 13C1H/D] (600 MHz) at a constant temperature of 25 °C. ^{19}F NMR spectra were recorded on a Bruker Avance III instrument with an Oxford AS600 magnet equipped with a cryoprobe [5 mm Prodigy] (500 MHz) at a constant temperature of 25 °C. ^{1}H and ^{13}C chemical shifts are reported in parts per million (ppm) from low to high field and referenced to the literature values for chemical shifts of residual non-deuterated solvent, with respect to tetramethylsilane (0.00 ppm) as an external standard. All ^{1}H resonances are reported to the nearest 0.01 ppm. The multiplicity of ^{1}H signals are indicated as: s = singlet; d = doublet; t = triplet; q = quartet; multiplet; br = broad; or combinations of thereof. Coupling constants (*J*) are quoted in Hz and reported to the nearest 0.1 Hz. Where appropriate, averages of the signals from peaks displaying multiplicity were used to calculate the value of the coupling constant. Flash column chromatography was carried out using Silica 60 Å (particle size 40–63 µm, Sigma Aldrich, UK) as the stationary phase. Analytical TLC was performed on precoated silica gel plates (0.25 mm thick, 60 F254, Merck, Germany) and visualized using both short and long wave ultraviolet light in combination with standard laboratory stains (acidic potassium permanganate, iodine vapor). Low resolution ESI mass spectrometry was performed with a Thermo Scientific LCQ Fleet Ion Trap Mass Spectrometer or an Agilent Technologies 1200 LC system with an Advion Expression CMS L single quadrupole MS detector. High-resolution mass spectrometry (HRMS) was carried out at the Mass Spectrometry Service, Department of Chemistry, University of Manchester. High-performance liquid chromatography (HPLC) was performed on an Agilent 1260 Infinity system. Column and conditions are specified

below. HPLC data were analyzed in Open Labs CDS software, and traces were exported as .csv data files for further plotting in Microsoft Excel.

Buffer preparation and pH reporting:

MES/MOPS Buffer stocks were made at a 1 M concentration in H_2O or D_2O, as appropriate, and adjusted to the desired pH/pH_{obs} by addition of HCl/DCl and NaOH/NaOD. The pH/pH_{obs} values recorded for individual experiments are those of these 1 M buffer solutions before dilution with organic and aqueous solvent as required.

Abbreviations:

APCI: Atmospheric-Pressure Chemical Ionization; Boc: *tert*-butoxycarbonyl; COSY: Correlated Spectroscopy; DEPT: Distortionless Enhancement by Polarization Transfer; DFT: Density Functional Theory; DIC: *N,N'*-Diisopropylcarbodiimide; DIPEA: *N*,*N*-diisopropylethylamine; DIU: Diisopropylurea; DMF: *N,N*-Dimethylformamide; DMSO: Dimethylsulfoxide; *e.e.*: enantiomeric excess; ESI: Electrospray Ionization; EtOAc: Ethyl Acetate; h: hour; HATU: Hexafluorophosphate Azabenzotriazole Tetramethyl Uronium; HMBC: Heteronuclear Multiple Bond Correlation; HOBt: Hydroxybenzotriazole; HPLC: High-Performance Liquid Chromatography; HRMS: High-Resolution Mass Spectrometry; HSQC: Heteronuclear Single Quantum Coherence; KHMDS: Potassium bis(trimethylsilyl)amide; MES: 2-(*N*-morpholino)ethanesulfonic acid**;** MOPS: 3-(*N*-morpholino)propanesulfonic acid; MWI: Microwave Irradiation; NMR: Nuclear Magnetic Resonance; pet: petroleum; ppm: parts per million; rt: room temperature; THF; Tetrahydrofuran; TLC: Thin Layer Chromatography.

S2. Synthesis and Characterization of Compounds

S2.1 Synthesis and characterization of motor compounds 1a–c

Compounds **1a**, **1b**, and **1c** were synthesized according to literature procedures and the structural characterization data was in agreement with reported values.[S1]

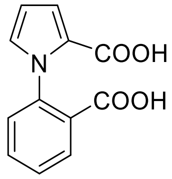

1a

^{1}H NMR (600 MHz, CD_3CN) δ 7.94 (dd, *J* = 7.7, 1.6 Hz, 1H), 7.61 (td, *J* = 7.7, 1.6 Hz, 1H), 7.52 (td, *J* = 7.7, 1.3 Hz, 1H), 7.28 (d, *J* = 7.8 Hz, 1H), 6.98 (dd, *J* = 4.0, 1.7 Hz, 1H), 6.92 (t, *J* = 2.1 Hz, 1H), 6.27 (dd, *J* = 3.9, 2.7 Hz, 1H).

^{13}C NMR (151 MHz, DMSO-d_6) δ 166.63, 161.61, 141.26, 133.36, 131.41, 130.96, 130.02, 129.85, 129.33, 125.10, 118.92, 109.82.

ESI-HRMS *m*/*z*: calcd. for $C_{12}H_9NO_4$ $[M+Na]^+$ 254.0424, found 254.0429.

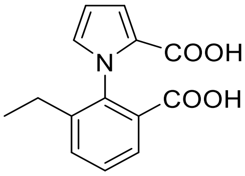

1b

^{1}H NMR (600 MHz, CD_3CN) δ 7.75 (dd, *J* = 7.7, 1.6 Hz, 1H), 7.55 (dd, *J* = 7.8, 1.6 Hz, 1H), 7.47 (t, *J* = 7.7 Hz, 1H), 7.00 (dd, *J* = 3.9, 1.7 Hz, 1H), 6.83 (dd, *J* = 2.6, 1.7 Hz, 1H), 6.30 (dd, *J* = 3.9, 2.6 Hz, 1H), 2.09 (s, 2H), 1.02 (t, *J* = 7.6 Hz, 3H).

^{13}C NMR (151 MHz, DMSO-d_6) δ 166.96, 161.56, 144.16, 139.49, 133.48, 130.98, 130.42, 129.43, 128.91, 125.01, 118.54, 109.93, 24.24, 15.13.

ESI-HRMS *m*/*z*: calcd. for $C_{14}H_{13}NO_4$ $[M-H]^-$ 258.0772, found 258.0762.

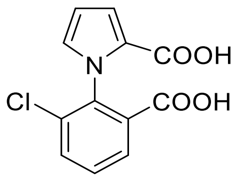

1c

^{1}H NMR (600 MHz, CD_3CN) δ 9.42 (s, 1H), 7.88 (d, *J* = 7.9 Hz, 1H), 7.72 (d, *J* = 8.1 Hz, 1H), 7.52 (t, *J* = 8.0 Hz, 1H), 7.02 (dd, *J* = 4.0, 1.8 Hz, 1H), 6.87 (t, *J* = 2.3 Hz, 1H), 6.33 (t, *J* = 3.4 Hz, 1H).

^{13}C NMR (151 MHz, DMSO-d_6) δ 165.93, 161.44, 138.76, 135.06, 133.88, 132.48, 130.58, 130.31, 130.03, 124.91, 118.61, 110.51.

ESI-HRMS *m*/*z*: calcd. for $C_{12}H_8ClNO_4$ $[M+Na]^+$ 288.0034, found 288.0041.

S2.2 Synthesis and characterization of carbodiimide fuels 2a–i

Ureas **3a–g** and Carbodiimides **2a–h** were synthesized according to a modified version of literature procedures (Scheme S1).[S2]

General procedure 1: synthesis of ureas **3a–f** and **3h**

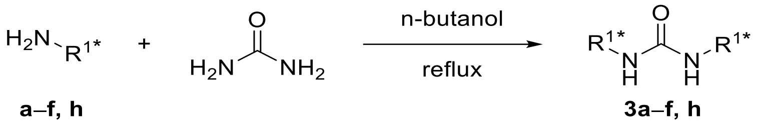

Scheme S1. Reagents and conditions for synthesis of urea compounds **3a–**f and **h**.

A solution of urea (1.0 equiv.) and amine **a–f** or **h** (2.4 equiv., see main text Figure 3 for R[1]* groups) in *n*-butanol (1.3 M) was refluxed until all the urea was consumed (around 16 h) and the desired product **3a–f, 3h** precipitated. The mixture was filtered through a sintered funnel and the precipitate washed with *n*-hexane and allowed to dry under air. Unless otherwise stated, the product was used without further purification.

Procedure: synthesis of urea **3g**

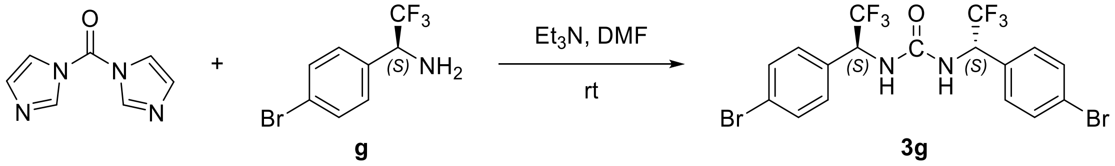

Scheme S2. Reagents and conditions forsynthesis of urea **3g**.

To a suspension of 1,1'-carbonyldiimidazole (100 mg, 0.62 mmol) in DMF (2.0 mL, 0.3 M) were added triethylamine (0.35 mL, 2.47 mmol) and amine **g** (314 mg, 1.24 mmol). The reaction mixture was stirred at room temperature overnight and then concentrated under the reduced pressure. Dilution with H_2O caused urea **3g** to precipitate. Washing with CH_2Cl_2 afforded the product in 40% yield (130 mg, 0.24 mmol).

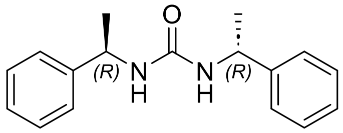

3a

Compound **3a** was synthesized according to general procedure 1 in 95% yield.

^{1}H NMR (600 MHz, DMSO-d_6) δ 7.31 (t, *J* = 7.5 Hz, 4H), 7.28 – 7.24 (m, 4H), 7.24 – 7.16 (m, 2H), 6.26 (d, *J* = 8.1 Hz, 2H), 4.70 (p, *J* = 7.2 Hz, 2H), 1.29 (d, *J* = 7.0 Hz, 6H).

^{13}C NMR (151 MHz, DMSO-d_6) δ 156.48, 145.75, 128.22, 126.44, 125.75, 48.54, 23.40.

APCI-HRMS *m*/*z*: calcd. for $C_{17}H_{20}N_2O$ $[M-H]^-$ 267.1503, found 267.1500.

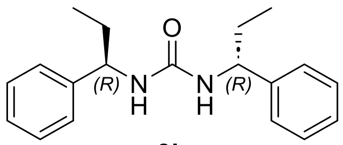

3b

Compound **3b** was synthesized according to general procedure 1 in 68% yield after flash column chromatography (SiO_2, EtOAc : *n*-hexane, 1:1 *v/v*).

^{1}H NMR (600 MHz, DMSO-d_6) δ 7.31 (t, *J* = 7.6 Hz, 4H), 7.25 – 7.18 (m, 6H), 6.27 (d, *J* = 8.5 Hz, 2H), 4.48 (q, *J* = 7.4 Hz, 2H), 1.67 – 1.54 (m, 4H), 0.75 (t, *J* = 7.3 Hz, 6H).

^{13}C NMR (151 MHz, DMSO-d_6) δ 157.06, 144.54, 128.17, 126.46, 126.23, 54.50, 29.94, 10.61.

ESI-HRMS *m*/*z*: calcd. for $C_{19}H_{24}N_2O$ $[M-H]^-$ 295.1816, found 295.1829.

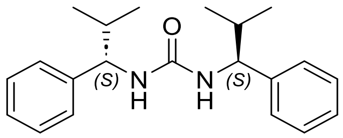

3c

Compound **3c** was synthesized according to general procedure 1 in 81% yield.

^{1}H NMR (600 MHz, DMSO-d_6) δ 7.34 – 7.28 (m, 4H), 7.23 – 7.17 (m, 6H), 6.38 (d, *J* = 9.2 Hz, 2H), 4.43 (dd, *J* = 9.1, 6.2 Hz, 2H), 1.88 (hept, *J* = 6.7 Hz, 2H), 0.75 (dd, *J* = 6.8, 2.3 Hz, 12H).

^{13}C NMR (151 MHz, DMSO-d_6) δ 157.35, 143.68, 127.95, 126.60, 126.31, 58.31, 33.49, 19.76, 17.85.

ESI-HRMS *m*/*z*: calcd. for $C_{21}H_{28}N_2O$ $[M+H]^+$ 325.2274, found 325.2266.

Note: δ = 19.76 and 17.85 are the methyl carbon signals.

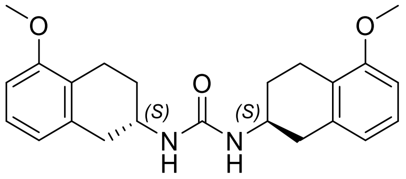

3d

Compound **3d** was synthesized according to general procedure 1 in 95% yield.

^{1}**H NMR** (600 MHz, DMSO-*d*$_6$) δ 7.06 (t, *J* = 7.9 Hz, 2H), 6.72 (d, *J* = 8.1 Hz, 2H), 6.64 (d, *J* = 7.6 Hz, 2H), 5.86 (d, *J* = 7.7 Hz, 2H), 3.85 – 3.76 (m, 2H), 3.74 (s, 6H), 2.91 (dd, *J* = 16.3, 4.8 Hz, 2H), 2.65 (dt, *J* = 17.8, 6.2 Hz, 2H), 2.58 – 2.47 (m, 4H), 1.89 – 1.82 (m, 2H), 1.62 – 1.53 (m, 2H).

^{13}**C NMR** (151 MHz, DMSO-*d*$_6$) δ 157.32, 156.83, 136.04, 126.42, 123.88, 121.51, 107.48, 55.20, 44.20, 36.00, 28.27, 21.00.

ESI-HRMS *m*/*z*: calcd. for $C_{23}H_{28}N_2O_3$ $[M+Na]^+$ 403.1992, found 403.1981.

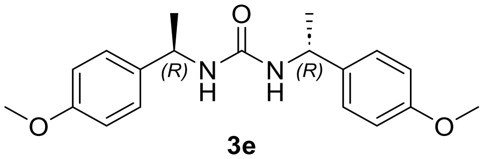

3e

Compound **3e** was synthesized according to general procedure 1 in 79% yield.

^{1}**H NMR** (600 MHz, DMSO-*d*$_6$) δ 7.18 (d, *J* = 8.8 Hz, 4H), 6.87 (d, *J* = 8.6 Hz, 4H), 6.13 (d, *J* = 8.1 Hz, 2H), 4.64 (p, *J* = 7.0 Hz, 2H), 3.72 (s, 6H), 1.25 (d, *J* = 6.9 Hz, 6H).

^{13}**C NMR** (151 MHz, DMSO-*d*$_6$) δ 157.91, 156.48, 137.71, 126.89, 113.59, 55.06, 47.89, 23.44.

ESI-HRMS *m*/*z*: calcd. for $C_{19}H_{24}N_2O_3$ $[M+Na]^+$ 351.1679, found 351.1692.

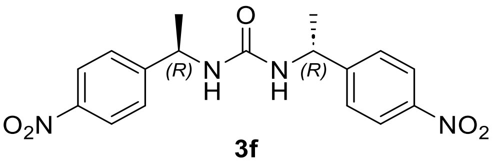

3f

Compound **3f** was synthesized according to general procedure 1 in 94% yield.

^{1}H NMR (600 MHz, DMSO-d_6) δ 8.18 (d, J = 8.6 Hz, 4H), 7.52 (d, J = 8.4 Hz, 4H), 6.58 (d, J = 7.5 Hz, 2H), 4.76 (p, J = 7.2 Hz, 2H), 1.31 (d, J = 7.0 Hz, 6H).

^{13}C NMR (151 MHz, DMSO-d_6) δ 156.53, 154.12, 146.29, 127.09, 123.70, 48.77, 22.79.

ESI-HRMS *m/z*: calcd. for $C_{17}H_{18}N_4O_5$ $[M-H]^-$ 357.1204, found 357.1214.

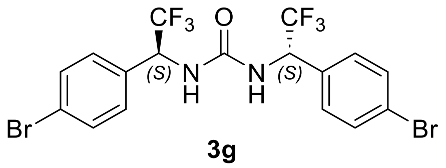

3g

^{1}H NMR (600 MHz, DMSO-d_6) δ 7.68 (d, J = 8.4 Hz, 4H), 7.46 (d, J = 9.7 Hz, 2H), 7.43 (d, J = 8.2 Hz, 4H), 5.65 – 5.53 (m, 2H).

^{13}C NMR (151 MHz, DMSO-d_6) δ 155.55, 133.06, 131.96, 130.25, 124.83 (q, J = 282.3 Hz) 122.66, 53.87 (q, J = 30.3 Hz).

^{19}F NMR (471 MHz, DMSO-d_6) δ -73.54 (d, J = 8.2 Hz).

APCI-HRMS *m/z*: calcd. for $C_{17}H_{12}Br_2F_6N_2O$ $[M+H]^+$ 534.9274, found 534.9275.

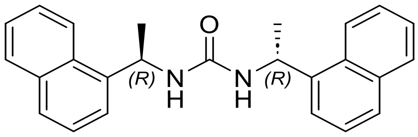

3h

Compound **3h** was synthesized according to general procedure 1 in 63% yield.

^{1}H NMR (600 MHz, DMSO-d_6) δ 8.13 (d, J = 8.4 Hz, 2H), 7.94 (d, J = 8.0 Hz, 2H), 7.84 – 7.79 (m, 2H), 7.58 – 7.50 (m, 8H), 6.44 (d, J = 8.1 Hz, 2H), 5.54 (p, J = 7.2 Hz, 2H), 1.43 (d, J = 6.9 Hz, 6H).

^{13}C NMR (151 MHz, DMSO-d_6) δ 156.43, 141.33, 133.44, 130.35, 128.65, 127.11, 126.12, 125.60, 125.54, 123.27, 121.95, 44.67, 22.55.

ESI-HRMS *m/z*: calcd. for $C_{25}H_{24}N_2O$ $[M+Na]^+$ 391.1781, found 391.1791.

General procedure 2: synthesis of carbodiimides **2a–h**

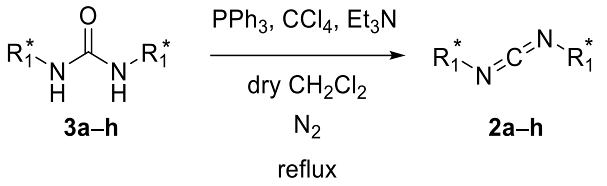

Scheme S3. Reagents and conditions for synthesis of carbodiimides **2a–h**.

Urea **3a–h** and triphenylphosphine (1.2 equiv., see main text Figure 3 for R_1* groups) were suspended in dry CH_2Cl_2 (1.6 M) under a nitrogen atmosphere. Then, carbon tetrachloride (1.0 equiv.) and triethylamine (1 equiv.) were added and the reaction mixture was refluxed for 16 h. The reaction mixture was cooled down to room temperature and diluted with CH_2Cl_2. All volatiles were removed under reduced pressure. The residue was purified by flash column chromatography.

Procedure: synthesis of carbodiimide **2i**.[S3]

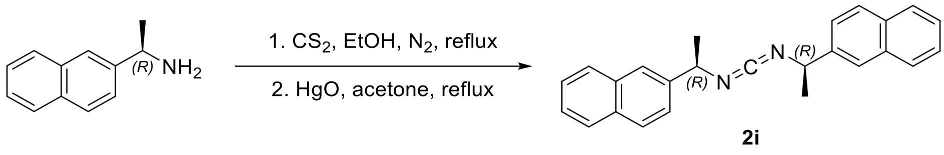

Scheme S4. Reagents and conditions for synthesis of carbodiimide **2i**.

(*R*)-1-(naphthalen-2-yl)ethan-1-amine (400 mg, 2.3 mmol) was dissolved in ethanol (2.3 mL, 1.0 M) under a nitrogen atmosphere. Then, CS_2 (0.28 mL, 4.6 mmol) was injected with a syringe. The reaction mixture was refluxed for 16 h and then cooled down to room temperature. Thiourea precipitated and was used without further purification. Thiourea (100 mg, 0.26 mmol) and mercuric (II) oxide (red, 113 mg, 0.52 mmol, 2 equiv.) were dissolved in acetone (0.90 mL, 0.3 M). The mixture was refluxed for 6 h. The formed mercuric sulfide was filtered off and a further 2.0 equiv. of mercuric (II) oxide (red) were added. The mixture was refluxed for a further 6 h and filtered again. The solution was concentrated under reduced pressure and flash chromatography (SiO_2, 100% CH_2Cl_2) afforded compound **2i** as a yellow oil in 15% yield (14 mg, 0.04 mmol).

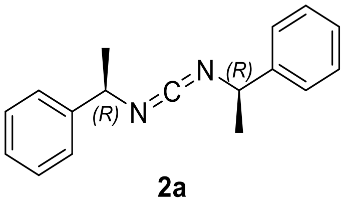

2a

Compound **2a** was synthesized[S2] according to general procedure 2 in 28% yield after flash column chromatography (SiO_2, 100% CH_2Cl_2).

^{1}H NMR (600 MHz, $CDCl_3$) δ 7.42 – 7.32 (m, 4H), 7.31 – 7.25 (m, 6H), 4.58 (q, *J* = 6.7 Hz, 2H), 1.50 (d, *J* = 6.8 Hz, 6H).

^{13}C NMR (151 MHz, $CDCl_3$) δ 143.71, 140.50, 128.58, 127.43, 126.06, 56.76, 24.75.

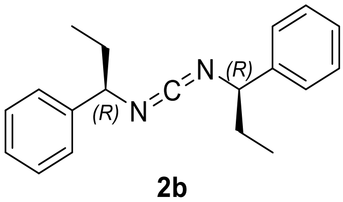

2b

Compound **2b** was synthesized according to general procedure 2 in 10% yield after flash column chromatography (SiO_2, CH_2Cl_2: *n*-hexane, 1:4 → 1:1).

^{1}H NMR (600 MHz, $CDCl_3$) δ 7.31 – 7.27 (m, 4H), 7.25 – 7.22 (m, 2H), 7.21 – 7.18 (m, 4H), 4.26 (t, *J* = 6.9 Hz, 2H), 1.75 (m, 4H), 0.86 (t, *J* = 7.4 Hz, 6H).

^{13}C NMR (151 MHz, $CDCl_3$) δ 142.69, 139.80, 128.55, 127.45, 126.65, 63.47, 31.92, 11.08.

ESI-HRMS *m*/*z*: calcd. for $C_{19}H_{22}N_2$ $[M+Na]^+$ 301.1675, found 301.1672.

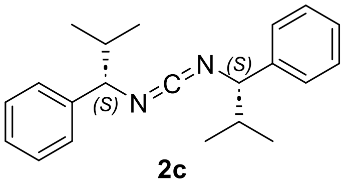

2c

Compound **2c** was synthesized according to general procedure 2 in 44% yield after flash column chromatography (SiO_2, 100% CH_2Cl_2).

^{1}H NMR (600 MHz, $CDCl_3$) δ 7.30 – 7.25 (m, 4H), 7.24 – 7.20 (m, 2H), 7.20 – 7.15 (m, 4H), 4.08 (d, *J* = 7.2 Hz, 2H), 1.89 (octet, *J* = 6.7 Hz, 2H), 0.90 (d, *J* = 6.6 Hz, 6H), 0.75 (d, *J* = 6.7 Hz, 6H).

^{13}C NMR (151 MHz, $CDCl_3$) δ 142.00, 139.05, 128.30, 127.27, 127.13, 68.42, 35.54, 19.79, 18.91.

ESI-HRMS *m*/*z*: calcd. for $C_{21}H_{26}N_2$ $[M+Na]^+$ 329.1988, found 329.1981.

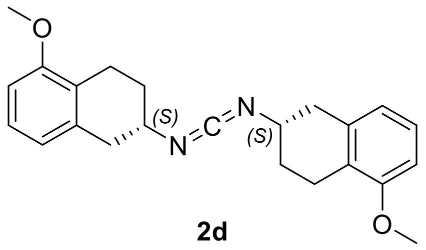

2d

Compound **2d** was synthesized according to general procedure 2 in 11% yield after flash column chromatography (SiO_2, 100% EtOAc).

^{1}H NMR (600 MHz, $CDCl_3$) δ 7.09 (t, *J* = 7.9 Hz, 2H), 6.70 – 6.61 (m, 4H), 3.79 (m, 8H), 3.60 (m, 2H), 2.95 (dd, *J* = 16.2, 4.8 Hz, 2H), 2.86 (dt, *J* = 17.9, 5.8 Hz, 2H), 2.71 (dd, *J* = 16.2, 8.3 Hz, 2H), 2.65 – 2.56 (m, 2H), 2.05 – 1.98 (m, 2H), 1.72 (m, 2H).

^{13}C NMR (151 MHz, $CDCl_3$) δ 157.26, 139.78, 135.62, 126.52, 124.48, 121.55, 107.39, 55.35, 52.42, 37.53, 30.28, 21.60.

ESI-HRMS *m*/*z*: calcd. for $C_{23}H_{26}N_2O_2$ $[M+Na]^+$ 385.1886, found 385.1879.

Note: 29.86 ppm in the carbon spectrum corresponds to the signal for grease.

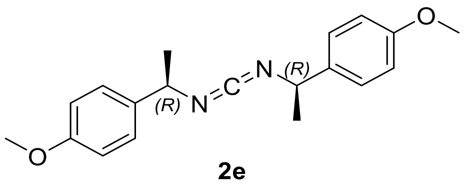

2e

Compound **2e** was synthesized according to general procedure 2 in 15% yield after flash column chromatography (SiO_2, 100% CH_2Cl_2).

^{1}H NMR (600 MHz, $CDCl_3$) δ 7.15 (d, *J* = 8.6 Hz, 4H), 6.84 (d, *J* = 8.6 Hz, 4H), 4.49 (q, *J* = 6.7 Hz, 2H), 3.80 (s, 6H), 1.44 (d, *J* = 6.8 Hz, 6H).

^{13}C NMR (151 MHz, $CDCl_3$) δ 158.89, 140.88, 136.05, 127.25, 113.89, 56.29, 55.39, 24.69.

ESI-HRMS *m*/*z*: calcd. for $C_{19}H_{22}N_2O_2$ $[M+Na]^+$ 333.1573, found 333.1571.

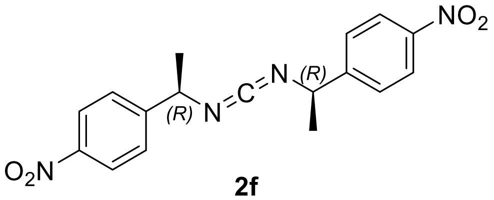

2f

Compound **2f** was synthesized according to general procedure 2 in 13% yield after flash chromatography (SiO_2, 100% CH_2Cl_2).

^{1}H NMR (600 MHz, $CDCl_3$) δ 8.19 (d, *J* = 8.8 Hz, 4H), 7.47 (d, *J* = 8.4 Hz, 4H), 4.76 (q, *J* = 6.8 Hz, 2H), 1.54 (d, *J* = 6.8 Hz, 6H).

^{13}C NMR (151 MHz, $CDCl_3$) δ 150.62, 147.36, 139.23, 126.91, 124.03, 56.16, 25.15.

ESI-HRMS *m*/*z*: calcd. for $C_{17}H_{16}N_4O_4$ $[M+Na]^+$ 363.1064, found 363.1071.

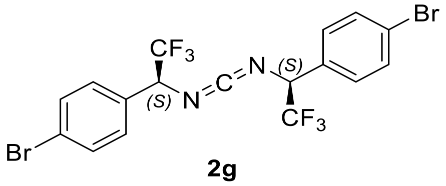

2g

Compound **2g** was synthesized according to general procedure 2 in 20% yield after flash chromatography (SiO_2, 100% CH_2Cl_2).

^{1}H NMR (600 MHz, $CDCl_3$) δ 7.51 (d, *J* = 8.5 Hz, 4H), 7.22 (d, *J* = 8.4 Hz, 4H), 4.93 (q, *J* = 6.9 Hz, 2H).

^{13}C NMR (151 MHz, $CDCl_3$) δ 137.13, 132.04, 131.23, 129.54, 123.19 (d, *J* = 281.7 Hz), 124.09, 61.41 (q, *J* = 31.9 Hz).

^{19}F NMR (471 MHz, $CDCl_3$) δ -75.53 (m).

ESI-HRMS *m*/*z*: calcd. for $C_{17}H_{10}Br_2F_6N_2$ $[M-H]^-$ 512.9042, found 512.9049.

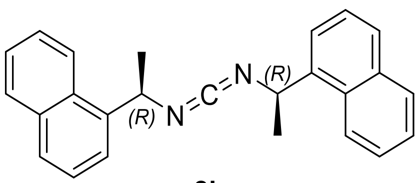

2h

Compound **2h** was synthesized according to general procedure 2 in 15% yield after flash column chromatography (SiO_2, 100% CH_2Cl_2).

^{1}H NMR (600 MHz, $CDCl_3$) δ 7.99 (m, 2H), 7.87 – 7.83 (m, 2H), 7.78 – 7.73 (m, 2H), 7.51 – 7.44 (m, 4H), 7.43 – 7.38 (m, 4H), 5.26 (q, *J* = 6.7 Hz, 2H), 1.52 (d, *J* = 6.7 Hz, 6H).

^{13}C NMR (151 MHz, $CDCl_3$) δ 139.13, 133.92, 130.40, 129.00, 128.05, 126.22, 125.64, 125.53, 123.22, 122.92, 53.01, 23.63.

APCI-HRMS *m*/*z*: calcd. for $C_{25}H_{22}N_2$ $[M+H]^+$ 351.1856, found 351.1842.

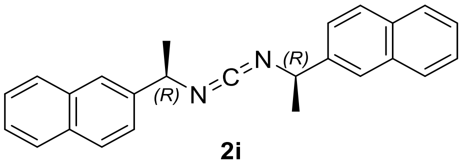

2i

Compound **2i** was synthesized according to a modified version of reported procedures[S3] in 15% yield after flash column chromatography (SiO_2, 100% CH_2Cl_2).

^{1}H NMR (600 MHz, $CDCl_3$) δ 7.78 (dt, *J* = 7.0, 3.6 Hz), 7.74 – 7.69 (m, 4H), 7.68 – 7.64 (m, 2H), 7.46 (dt, *J* = 6.2, 3.4 Hz, 4H), 7.32 (dd, *J* = 8.5, 1.8 Hz, 2H), 4.72 (q, *J* = 6.7 Hz, 2H), 1.55 (d, *J* = 6.7 Hz, 6H).

^{13}C NMR (151 MHz, $CDCl_3$) δ 141.11, 140.62, 133.38, 132.87, 128.44, 128.08, 127.77, 126.26, 125.94, 124.58, 124.42, 57.01, 24.76.

APCI-HRMS *m*/*z*: calcd. for $C_{25}H_{22}N_2$ $[M+H]^+$ 351.1856, found 351.1851.

S2.3 Synthesis and characterization of hydrolysis promoters 4a–h

Compounds (*R*)-**4a** and (*S*)-**4a** were synthesized in accordance with previous publications. All spectral data were in agreement with reported values.[S4]

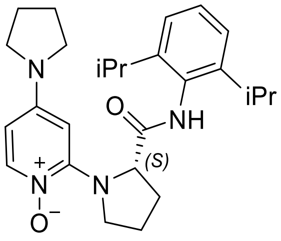

(S)-**4b**

Compounds (*R*)-**4b** and (*S*)-**4b** were synthesized in accordance with previous publications. All spectral data were in agreement with reported values.[S5, S6]

^{1}H NMR (600 MHz, $CDCl_3$) δ 9.82 (s, 1H), 7.79 (d, *J* = 7.4 Hz, 1H), 7.19 (t, *J* = 7.7 Hz, 1H), 7.06 (d, *J* = 7.8 Hz, 2H), 6.03 (dd, *J* = 7.4, 3.0 Hz, 1H), 5.80 (d, *J* = 3.0 Hz, 1H), 5.52 (t, *J* = 6.8 Hz, 1H), 4.08 – 4.01 (m, 1H), 3.40 (ddd, *J* = 9.6, 8.0, 4.1 Hz, 1H), 3.34 – 3.27 (m, 4H), 2.74 (s, 2H), 2.45 (td, *J* = 13.4, 7.9 Hz, 1H), 2.38 – 2.30 (m, 1H), 2.29 – 2.21 (m, 1H), 2.09 – 1.95 (m, 5H), 1.00 (d, *J* = 6.9 Hz, 12H).

^{13}C NMR (151 MHz, $CDCl_3$) δ 171.28, 150.65, 147.56, 145.89, 140.29, 131.57, 127.70, 123.04, 100.84, 94.16, 63.15, 51.10, 47.60, 29.13, 28.46, 25.43, 24.22, 23.70.

ESI-HRMS *m*/*z*: calcd. for $C_{26}H_{36}N_4O_2$ $[M+H]^+$ 437.2912, found 437.2899.

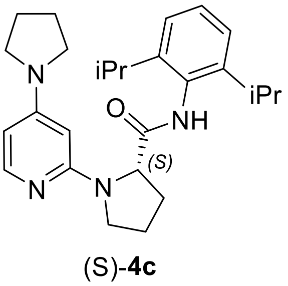

(S)-**4c**

Compound (*S*)-**4c** was synthesized in accordance with previous publications. All spectral data were in agreement with reported values (Scheme S6).[S5]

^{1}H NMR (600 MHz, $CDCl_3$) δ 8.70 (s, 1H), 7.84 (d, *J* = 6.0 Hz, 1H), 7.22 (t, *J* = 7.7 Hz, 1H), 7.11 (d, *J* = 7.7 Hz, 2H), 5.97 (d, *J* = 3.9 Hz, 1H), 5.54 (s, 1H), 4.71 (s, 1H), 3.70

(t, J = 7.9 Hz, 1H), 3.45 – 3.40 (m, 1H), 3.35 – 3.28 (m, 4H), 3.08 – 3.00 (m, 2H), 2.56 – 2.50 (m, 1H), 2.17 – 2.09 (m, 3H), 2.04 – 1.99 (m, 4H), 1.14 (d, J = 6.6 Hz, 6H), 1.08 (d, J = 6.8 Hz, 6H).

^{13}C NMR (151 MHz, $CDCl_3$) δ 173.28, 158.61, 153.42, 146.09, 131.61, 127.81, 123.17, 123.17, 99.90, 87.99, 61.95, 48.41, 47.05, 28.59, 25.30, 24.76, 23.61, 23.46.

ESI-HRMS *m*/*z*: calcd. for $C_{26}H_{36}N_4O$ $[M+H]^+$ 421.2983, found 421.3001.

Compound (*S*)-**4d** was synthesised according to a modified version of a procedure reported in previous publications (Scheme S5).[S4]

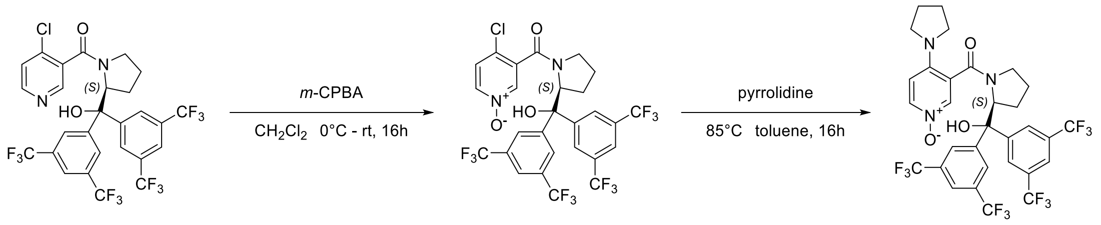

S1 **S2** (*S*)-**4d**

Scheme S5. Reagents and conditions for synthesis of compound (*S*)-**4d**.[S4]

Procedure: Synthesis of chloropyridine-oxide **S2**

Chloropyridine **S1** (0.750 g, 1.13 mmol, 1.0 eq.) and m-CPBA (0.487 g, 2.82 mmol, 2.5 eq.) were placed in a DCM (42 mL) solution at 0 °C. The reaction mixture was stirred at room temperature for 24 h. Upon completion of the reaction, DCM was removed under reduced pressure. Subsequently, brine (50 mL) was added, and the resulting suspension was extracted with dichloromethane (3 × 100 mL). The combined organic phases were dried over anhydrous Na_2SO_4, filtered, and the solvents evaporated under reduced pressure. Purification through flash column chromatography on silica gel using DCM/MeOH = 10/1 as eluent afforded **S2** as a colorless solid (0.625 g, 81%).

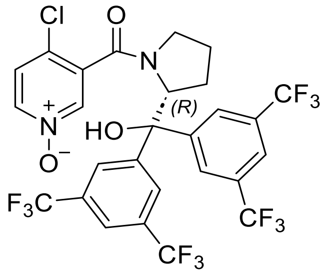

S2

^{1}H NMR (600 MHz, Acetone-*d_6*) δ 8.50 (s, 2H), 8.31 (s, 2H), 8.18 – 8.10 (m, 1H), 8.04 (d, 2H), 7.87 (s, 1H), 7.49 (s, 1H), 6.57 – 6.28 (m, 1H), 5.93 (s, 1H), 3.61 (q, J = 10.0 Hz, 1H), 3.56 – 3.45 (m, 1H), 2.35 – 2.29 (m, 1H), 2.08 – 2.06 (m, 1H), 2.01 – 1.94 (m, 1H), 1.89 – 1.81 (m, 1H).

^{13}C NMR (151 MHz, Acetone-*d_6*) δ 164.21, 148.77, 148.37, 141.01, 138.52, 136.30, 131.78 (dq, J = 165.6, 33.0 Hz), 128.52 (d, J = 49.0 Hz), 128.35, 124.48 (qd, J = 272.1, 22.0 Hz), 122.26 (d, J = 83.1 Hz), 81.84, 64.50, 51.48, 28.71, 25.60.

^{19}F NMR (471 MHz, $CDCl_3$) δ -62.82 (d, *J* = 112.5 Hz).

ESI-HRMS *m*/*z*: calcd. for $C_{27}H_{17}ClF_{12}N_2O_3$ $[M+H]^+$ 681.0809, found 681.0785.

Procedure: Synthesis of (*S*)-**4d**

Chloropyridine oxide **S2** (0.400 g, 0.587 mmol, 1.0 eq.) was placed in a pyrrolidine (2 ml) solution and degassed under a stream of nitrogen for 20 min. The reaction mixture was then sealed and heated at 80 °C for 16 h. After cooling to room temperature, pyrrolidine was removed under reduced pressure. Subsequently, brine (50 mL) was added, and the resulting suspension was extracted with dichloromethane (3 × 100 mL). The combined organic phases were dried over anhydrous Na_2SO_4, filtered, and the solvents evaporated under reduced pressure. Purification through flash column chromatography on silica gel using DCM/MeOH = 50/1 as eluent afforded (*S*)-**4d** as a brown solid (0.319 g, 76%).

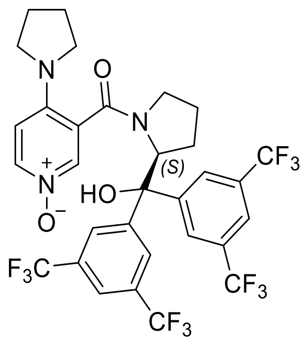

(*S*)-**4d**

^{1}H NMR (600 MHz, $CDCl_3$) δ 9.76 (s, 1H), 8.20 (s, 2H), 8.15 (s, 2H), 8.09 (dd, *J* = 7.3, 2.2 Hz, 1H), 7.78 (s, 1H), 7.53 (s, 1H), 6.55 (d, *J* = 7.4 Hz, 1H), 5.70 (t, *J* = 7.1 Hz, 1H), 4.00 (td, *J* = 10.4, 6.7 Hz, 1H), 3.69 (ddd, *J* = 11.0, 7.8, 3.0 Hz, 1H), 3.46 (td, *J* = 9.7, 6.3 Hz, 2H), 3.08 – 2.99 (m, 2H), 2.17 – 2.09 (m, 2H), 2.07 – 2.01 (m, 1H), 1.98 – 1.90 (m, 4H), 1.66 – 1.58 (m, 1H).

^{13}C NMR (151 MHz, $CDCl_3$) δ 167.06, 148.30, 147.03, 144.68, 139.13 (d, *J* = 375.3 Hz), 131.11 (dq, *J* = 160.8, 33.1 Hz), 123.25 (dd, *J* = 272.8, 31.0 Hz), 123.25 (qd, *J* = 272.8, 31.0 Hz) ,120.96 (d, *J* = 37.4 Hz), 117.93, 109.83, 80.64, 62.14, 53.15, 50.36, 28.07, 25.59, 24.97.

^{19}F NMR (471 MHz, $CDCl_3$) δ -62.89 (d, *J* = 254.5 Hz).

ESI-HRMS *m*/*z*: calcd. for $C_{31}H_{25}F_{12}N_3O_3$ $[M+H]^+$ 716.1777, found 716.1745.

Compounds **4e–g** were synthesized in accordance with previous publications.[S7] All spectral data were in agreement with reported values.

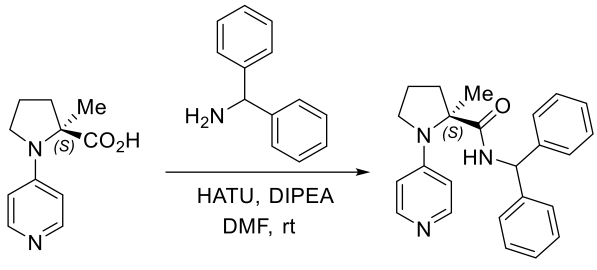

S7

(*S*)-**4h**

Scheme S6. Reagents and conditions for synthesis of (*S*)-**4h**.

Procedure: Synthesis of (*S*)-**4h.** [S7]

Compound **S7** (0.05 mmol) was stirred in the presence of HATU (1.2 equiv.) and DIPEA (2.5 equiv.) for 1 hour at room temperature before addition of diphenylmethanamine (2 equiv.). The reaction was stirred overnight at room temperature. The reaction mixture was then diluted with H_2O (30 mL) and extracted with EtOAc (3 x 20 mL). Compound (*S*)-**4h** was purified by column chromatography (CH_2Cl_2 to CH_2Cl_2/MeOH = 8/2 v/v) in 20% yield.

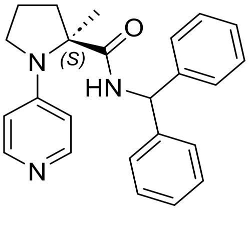

(*S*)-**4h**

^{1}H NMR (600 MHz, CD_3OD) δ 7.98 (d, *J* = 6.1 Hz, 2H), 7.33 (t, *J* = 7.5 Hz, 2H), 7.27 (t, *J* = 7.3 Hz, 1H), 7.22 (d, *J* = 7.7 Hz, 2H), 7.18 (d, *J* = 6.4 Hz, 3H), 7.01 (d, *J* = 6.8 Hz, 2H), 6.50 (d, *J* = 6.0 Hz, 2H), 6.27 (s, 1H), 3.79 (q, *J* = 7.4 Hz, 1H), 3.53 (q, *J* = 7.0 Hz, 1H), 2.38 – 2.31 (m, 1H), 2.19 – 2.07 (m, 3H), 1.57 (s, 3H).

^{13}C NMR (151 MHz, CD_3OD) δ 175.63, 153.06, 147.01, 142.62, 142.22, 129.50, 129.26, 129.18, 128.82, 128.41, 128.39, 110.59, 68.87, 58.31, 50.53, 42.83, 23.60, 19.89. (the carbon atoms of the phenyl groups are not equivalent).

ESI-HRMS *m*/*z*: calcd. for $C_{24}H_{25}N_3O$ $[M+H]^+$ 372.2070, found 372.2084.

S3. Monitoring fuel consumption during autonomous motor operation using ^{1}H NMR

S3.1 Monitoring the consumption of DIC and fuels 2a–i

Preparation of samples

[Racemic (±)-**1b**] = 1.0 mM, [DMAP] = 1.0 mM, [Fuel **2a**–**i**] = 10.0 mM, [MES buffer] = 100 mM (pH_{obs} 5.10 in D_2O), CD_3CN:D_2O (7:3 *v/v*) at r.t.

In an NMR tube, racemic (±)-**1b** (5.0 μL of a 0.1 M stock solution in DMF) was diluted into 350 μL of CD_3CN. To the solution was added 100 μL of D_2O, 100 mM of MES buffer (50 μL of a 1.0 M stock solution in D_2O, pH_{obs} 5.10), and DMAP (2.5 μL of a 0.2 M stock solution in CD_3CN:D_2O, 1:1 *v/v*). Carbodiimide fuel (25 μL of a 0.2 M stock solution in CD_3CN, 10.0 mM) was added and the reaction was monitored over time by ^{1}H NMR spectroscopy. Background experiments were performed under identical conditions but lacking racemic (±)-**1b**. Concentration data were obtained from the relative integrals of the ^{1}H NMR signals corresponding to the aliphatic protons of fuels **2a**–**i** (Figures S1–9C). The data were used to fit a line produced by a *pseudo*-first order rate equation using non-linear regression, by varying the *pseudo*-first order rate constant (k_{obs}) and the initial concentration (Figures S1–9B and S1–9D). Comparison of these k_{obs} values with that of the uncatalyzed background reaction was used to assess the percentage of fuel that was used by **1b** during motor operation (Figures S1–9E).

A

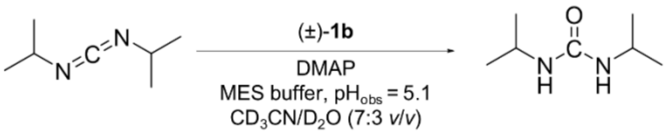

B

Reaction	k_{obs} (s^{-1})	k_{cat} (s^{-1})	Rate enhancement	$t_{1/2}$ (h)	% Fuel consumption
Background	6.85 x 10^{-6}	-	-	28.1	22
Catalysed	2.70 x 10^{-5}	2.02 x 10^{-5}	3.9	7.1	78

C

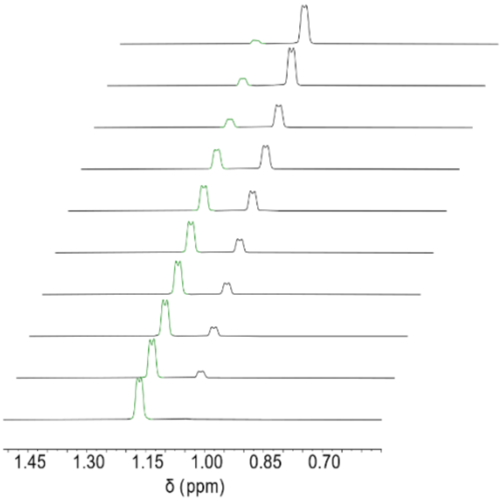

D

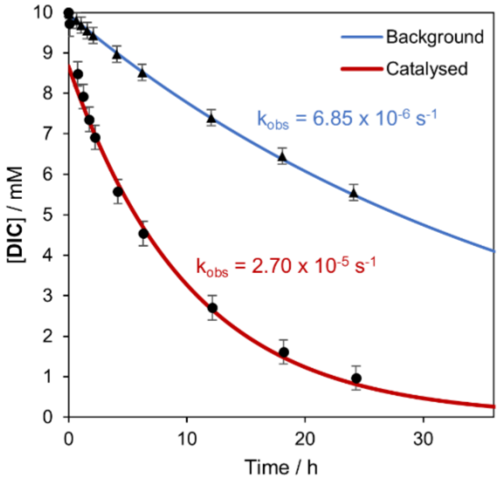

Figure S1. Kinetics of fuel consumption catalyzed by **1b.** (**A**) [Racemic (±)-**1b**] = 1.0 mM, [DMAP] = 1.0 mM, [DIC] = 10.0 mM, [MES buffer] = 100 mM (pH_{obs} 5.10 in D_2O) CD_3CN:D_2O (7:3 *v/v*) at r.t. (**B**) Table showing the observed rate constant k_{obs}, the computed catalyzed rate constant k_{cat}, the rate enhancement factor of the consumption of fuel by the catalyst **1b**, and the half-life of the fuel for the background and catalyzed pathways pertaining to the fueling reaction shown in panel **A**. Rate enhancement corresponds to 78% of fuel molecules reacting via the machine-catalyzed pathway. (**C**) Partial ^{1}H NMR spectra (MES-buffered (100 mM, pH_{obs} 5.10) CD_3CN:D_2O (7:3 *v/v*), 600 MHz, 298 K) showing the disappearance of DIC fuel and appearance of urea waste (CH_3 signals). (**D**) Kinetics of carbodiimide hydration in the absence and presence of **1b** (1.0 mM), determined by ^{1}H NMR spectroscopy. Solid lines represent the fit to pseudo-first order kinetics (k_{obs}).

A

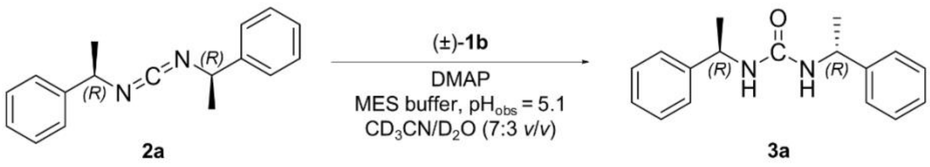

B

Reaction	k_{obs} (s^{-1})	k_{cat} (s^{-1})	Rate enhancement	$t_{1/2}$ (h)	% Fuel consumption
Background	1.84 x 10^{-6}	-	-	104.8	14
Catalysed	1.25 x 10^{-5}	1.06 x 10^{-5}	6.8	15.4	86

C

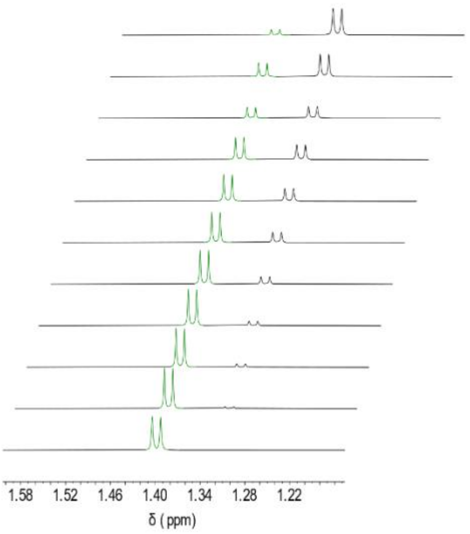

D

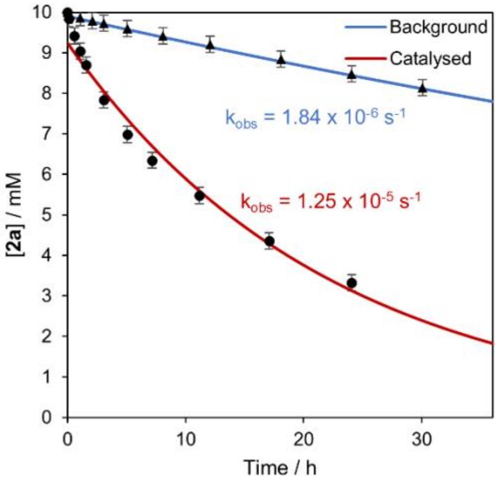

Figure S2. Kinetics of fuel consumption catalyzed by **1b.** (**A**) [Racemic (±)-**1b**] = 1.0 mM, [DMAP] = 1.0 mM, [**2a**] = 10.0 mM, [MES buffer] = 100 mM (pH$_{obs}$ 5.10 in D_2O) $CD_3CN:D_2O$ (7:3 *v/v*) at r.t. (**B**) Table showing the observed rate constant k_{obs}, the computed catalyzed rate constant k_{cat}, the rate enhancement factor of the consumption of fuel by the catalyst **1b**, and the half-life of the fuel for the background and catalyzed pathways pertaining to the fueling reaction shown in panel **A**. Rate enhancement corresponds to 86% of fuel molecules reacting via the machine-catalyzed pathway. (**C**) Partial ^{1}H NMR spectra (MES-buffered (100 mM, pH$_{obs}$ 5.10) $CD_3CN:D_2O$ (7:3 *v/v*), 600 MHz, 298 K) showing the disappearance of **2a** fuel and appearance of **3a** urea waste (CH_3 signals). (**D**) Kinetics of carbodiimide hydration in the absence and presence of **1b** (1.0 mM), determined by ^{1}H NMR spectroscopy. Solid lines represent the fit to pseudo-first order kinetics (k_{obs}).

A

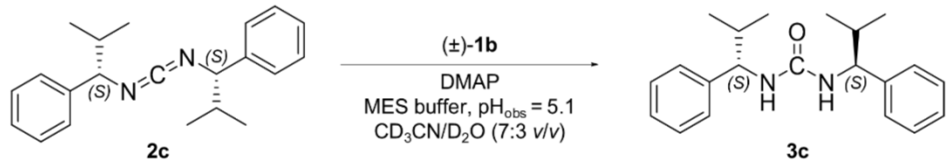

B

Reaction	k_{obs} (s^{-1})	k_{cat} (s^{-1})	Rate enhancement	$t_{1/2}$ (h)	% Fuel consumption
Background	4.59 x 10^{-7}	-	-	419.3	2
Catalysed	1.70 x 10^{-5}	1.65 x 10^{-5}	37.0	11.3	98

C **D**

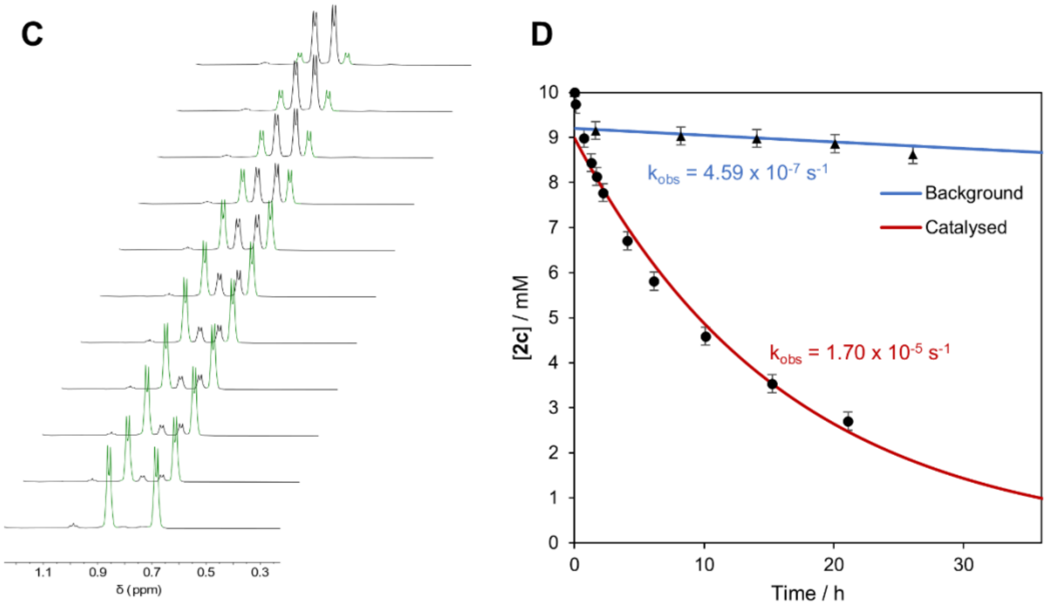

Figure S3. Kinetics of fuel consumption catalyzed by **1b.** (**A**) [Racemic (±)-**1b**] = 1.0 mM, [DMAP] = 1.0 mM, [**2c**] = 10.0 mM, [MES buffer] = 100 mM (pH$_{obs}$ 5.10 in D_2O) $CD_3CN:D_2O$ (7:3 *v/v*) at r.t. (**B**) Table showing the observed rate constant k_{obs}, the computed catalyzed rate constant k_{cat}, the rate enhancement factor of the consumption of fuel by the catalyst **1b**, and the half-life of the fuel for the background and catalyzed pathways pertaining to the fueling reaction shown in panel **A**. Rate enhancement corresponds to 98% of fuel molecules reacting via the machine-catalyzed pathway. (**C**) Partial ^{1}H NMR spectra (MES-buffered (100 mM, pH$_{obs}$ 5.10) $CD_3CN:D_2O$ (7:3 *v/v*), 600 MHz, 298 K) showing the disappearance of **2c** fuel and appearance of **3c** urea waste (CH_3 signals). (**D**) Kinetics of carbodiimide hydration in the absence and presence of **1b** (1.0 mM), determined by ^{1}H NMR spectroscopy. Solid lines represent the fit to pseudo-first order kinetics (k_{obs}).

A

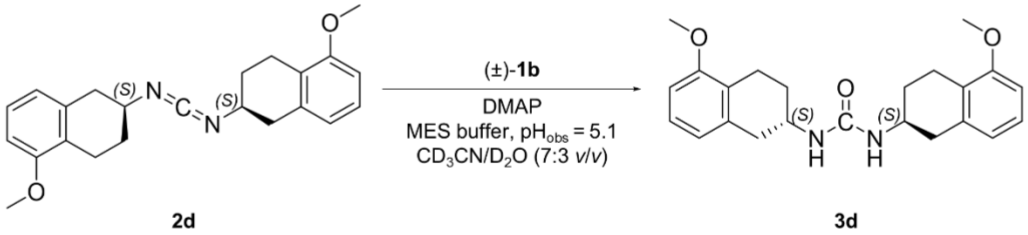

B

Reaction	k_{obs} (s^{-1})	k_{cat} (s^{-1})	Rate enhancement	$t_{1/2}$ (h)	% Fuel consumption
Background	2.39 x 10^{-6}	-	-	80.5	5
Catalysed	4.41 x 10^{-5}	4.18 x 10^{-5}	18.5	4.4	95

C

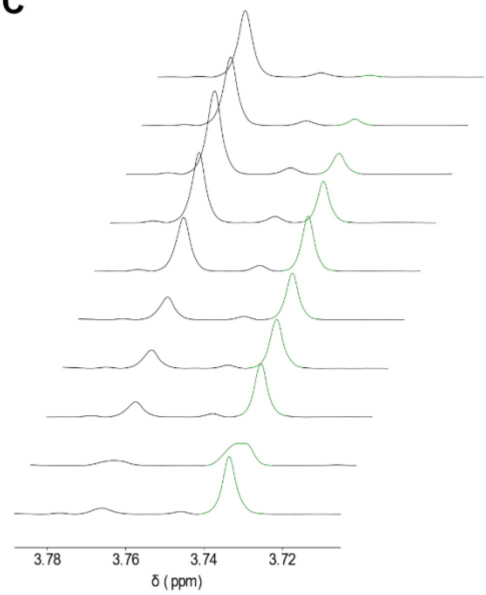

D

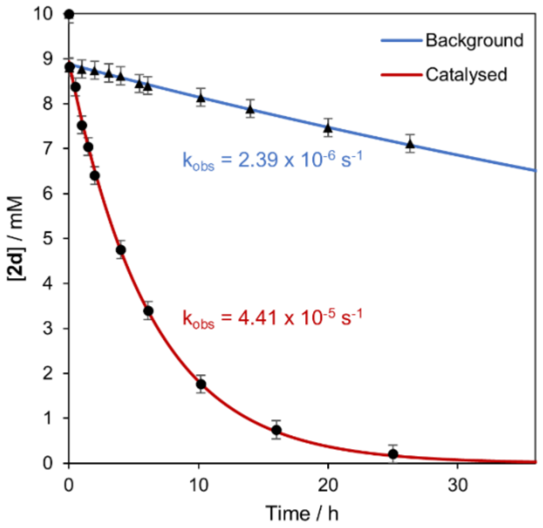

Figure S4. Kinetics of fuel consumption catalyzed by **1b.** (**A**) [Racemic (±)-**1b**] = 1.0 mM, [DMAP] = 1.0 mM, [**2d**] = 10.0 mM, [MES buffer] = 100 mM (pH_{obs} 5.10 in D_2O) CD_3CN:D_2O (7:3 *v/v*) at r.t. (**B**) Table showing the observed rate constant k_{obs}, the computed catalyzed rate constant k_{cat}, the rate enhancement factor of the consumption of fuel by the catalyst **1b**, and the half-life of the fuel for the background and catalyzed pathways pertaining to the fueling reaction shown in panel **A**. Rate enhancement corresponds to 95% of fuel molecules reacting via the machine-catalyzed pathway. (**C**) Partial ^{1}H NMR spectra (MES-buffered (100 mM, pH_{obs} 5.10) CD_3CN:D_2O (7:3 *v/v*), 600 MHz, 298 K) showing the disappearance of **2d** fuel and appearance of **3d** urea waste (CH_3 signals). (**D**) Kinetics of carbodiimide hydration in the absence and presence of **1b** (1.0 mM), determined by ^{1}H NMR spectroscopy. Solid lines represent the fit to pseudo-first order kinetics (k_{obs}).

A

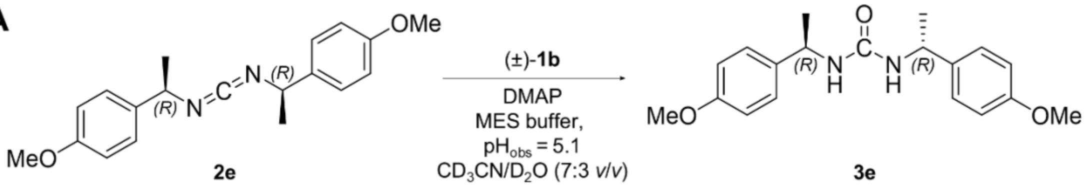

B

Reaction	k_{obs} (s^{-1})	k_{cat} (s^{-1})	Rate enhancement	$t_{1/2}$ (h)	% Fuel consumption
Background	1.43×10^{-6}	-	-	135.0	10
Catalysed	1.25×10^{-5}	1.11×10^{-5}	8.8	15.4	90

C **D**

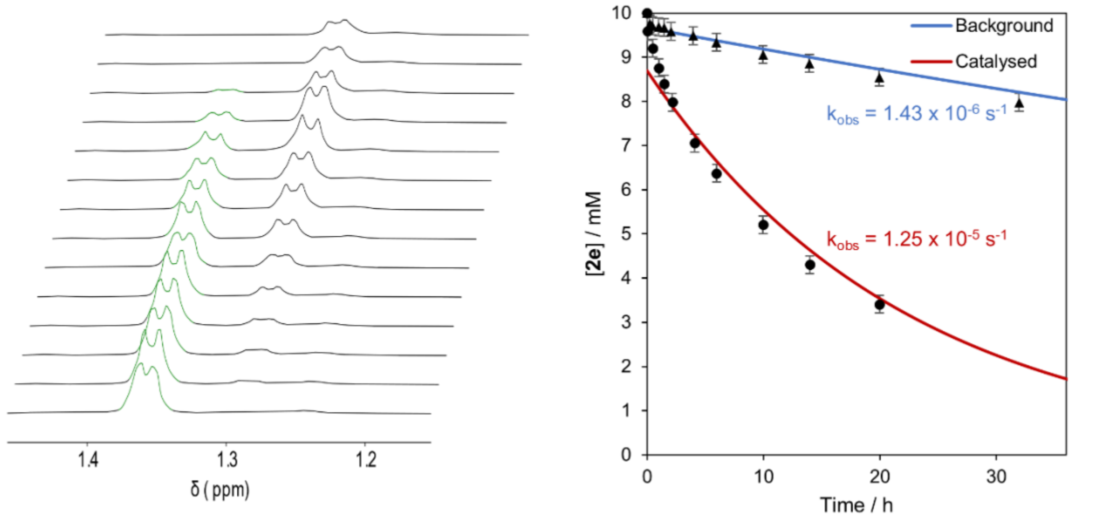

Figure S5. Kinetics of fuel consumption catalyzed by **1b.** (**A**) [Racemic (±)-**1b**] = 1.0 mM, [DMAP] = 1.0 mM, [**2e**] = 10.0 mM, [MES buffer] = 100 mM (pH_{obs} 5.10 in D_2O) CD_3CN:D_2O (7:3 *v/v*) at r.t. (**B**) Table showing the observed rate constant k_{obs}, the computed catalyzed rate constant k_{cat}, the rate enhancement factor of the consumption of fuel by the catalyst **1b**, and the half-life of the fuel for the background and catalyzed pathways pertaining to the fueling reaction shown in panel **A**. Rate enhancement corresponds to 90% of fuel molecules reacting via the machine-catalyzed pathway. (**C**) Partial ^{1}H NMR spectra (MES-buffered (100 mM, pH_{obs} 5.10) CD_3CN:D_2O (7:3 *v/v*), 600 MHz, 298 K) showing the disappearance of **2e** fuel and appearance of **3e** urea waste (CH_3 signals). (**D**) Kinetics of carbodiimide hydration in the absence and presence of **1b** (1.0 mM), determined by ^{1}H NMR spectroscopy. Solid lines represent the fit to pseudo-first order kinetics (k_{obs}).

A

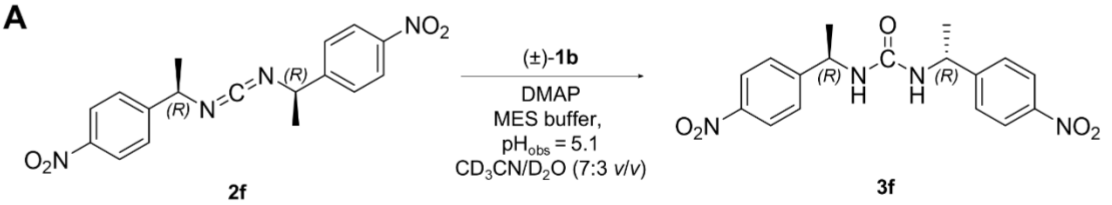

B

Reaction	k_{obs} (s^{-1})	k_{cat} (s^{-1})	Rate enhancement	$t_{1/2}$ (h)	% Fuel consumption
Background	6.16 x 10^{-7}	-	-	312.3	10
Catalysed	6.02 x 10^{-6}	5.40 x 10^{-6}	9.8	32.0	90

C **D**

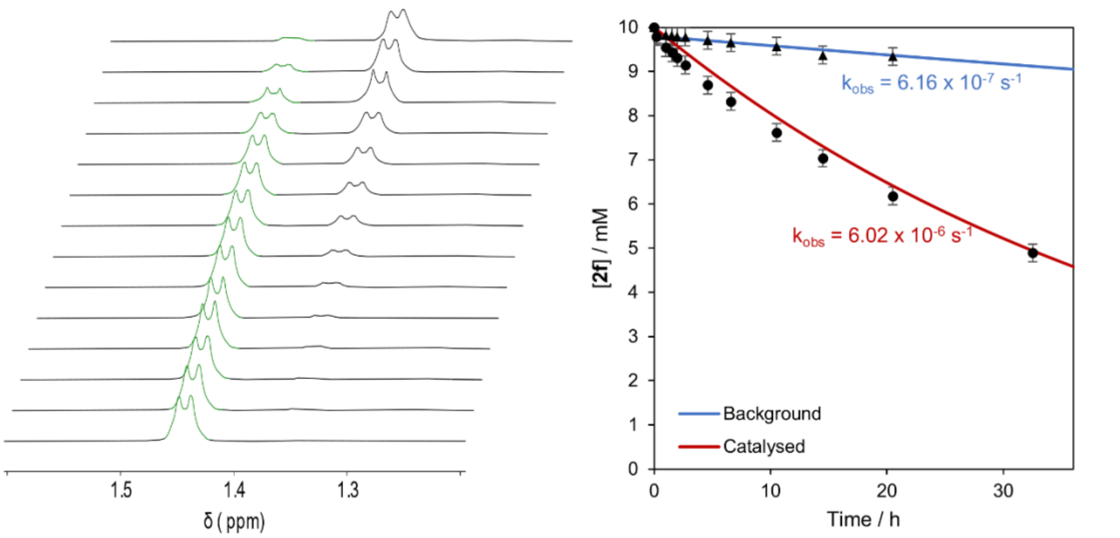

Figure S6. Kinetics of fuel consumption catalyzed by **1b.** (**A**) [Racemic (±)-**1b**] = 1.0 mM, [DMAP] = 1.0 mM, [**2f**] = 10.0 mM, [MES buffer] = 100 mM (pH_{obs} 5.10 in D_2O) CD_3CN:D_2O (7:3 *v/v*) at r.t. (**B**) Table showing the observed rate constant k_{obs}, the computed catalyzed rate constant k_{cat}, the rate enhancement factor of the consumption of fuel by the catalyst **1b**, and the half-life of the fuel for the background and catalyzed pathways pertaining to the fueling reaction shown in panel **A**. Rate enhancement corresponds to 90% of fuel molecules reacting via the machine-catalyzed pathway. (**C**) Partial ^{1}H NMR spectra (MES-buffered (100 mM, pH_{obs} 5.10) CD_3CN:D_2O (7:3 *v/v*), 600 MHz, 298 K) showing the disappearance of **2f** fuel and appearance of **3f** urea waste (CH_3 signals). (**D**) Kinetics of carbodiimide hydration in the absence and presence of **1b** (1.0 mM), determined by ^{1}H NMR spectroscopy. Solid lines represent the fit to pseudo-first order kinetics (k_{obs}).

A

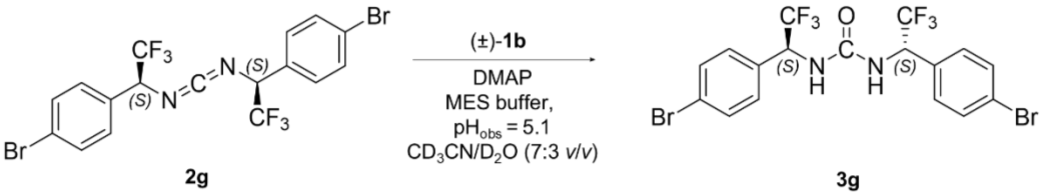

B

Reaction	k_{obs} (s^{-1})	k_{cat} (s^{-1})	Rate enhancement	$t_{1/2}$ (h)	% Fuel consumption
Background	2.46 x 10^{-6}	-	-	78.3	27
Catalysed	8.94 x 10^{-6}	6.49 x 10^{-6}	3.6	21.5	73

C

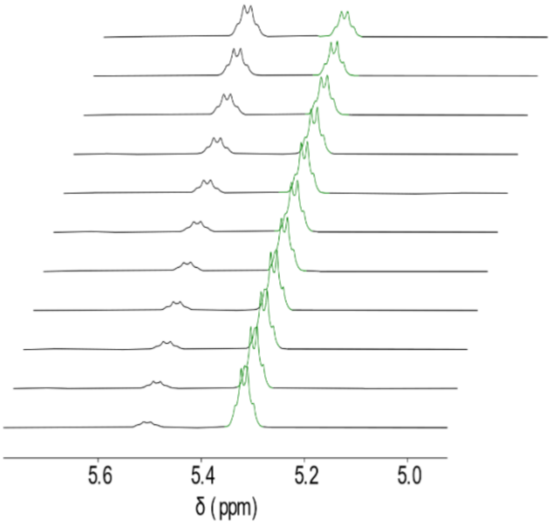

D

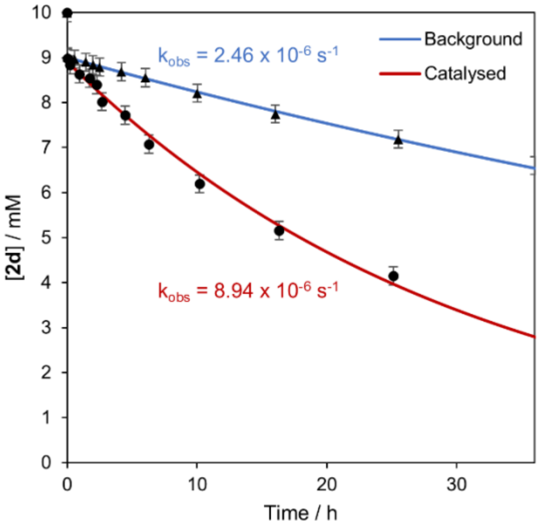

Figure S7. Kinetics of fuel consumption catalyzed by **1b.** (**A**) [Racemic (±)-**1b**] = 1.0 mM, [DMAP] = 1.0 mM, [**2g**] = 10.0 mM, [MES buffer] = 100 mM (pH_{obs} 5.10 in D_2O) CD_3CN:D_2O (7:3 *v/v*) at r.t. (**B**) Table showing the observed rate constant k_{obs}, the computed catalyzed rate constant k_{cat}, the rate enhancement factor of the consumption of fuel by the catalyst **1b**, and the half-life of the fuel for the background and catalyzed pathways pertaining to the fueling reaction shown in panel **A**. Rate enhancement corresponds to 73% of fuel molecules reacting via the machine-catalyzed pathway. (**C**) Partial ^{1}H NMR spectra (MES-buffered (100 mM, pH_{obs} 5.10) CD_3CN:D_2O (7:3 *v/v*), 600 MHz, 298 K) showing the disappearance of **2g** fuel and appearance of **3g** urea waste (CH signals). (**D**) Kinetics of carbodiimide hydration in the absence and presence of **1b** (1.0 mM), determined by ^{1}H NMR spectroscopy. Solid lines represent the fit to pseudo-first order kinetics (k_{obs}).

A

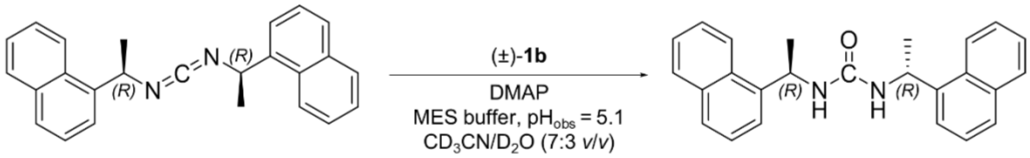

2h **3h**

B

Reaction	k_{obs} (s^{-1})	k_{cat} (s^{-1})	Rate enhancement	$t_{1/2}$ (h)	% Fuel consumption
Background	1.18 x 10^{-6}	-	-	163.3	12
Catalysed	1.11 x 10^{-5}	9.95 x 10^{-6}	9.4	17.3	88

C

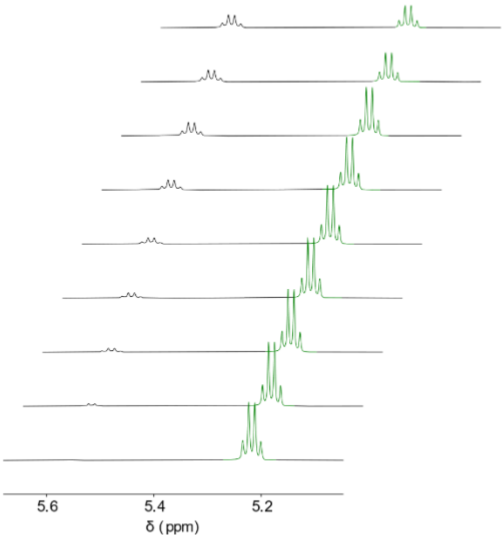

D

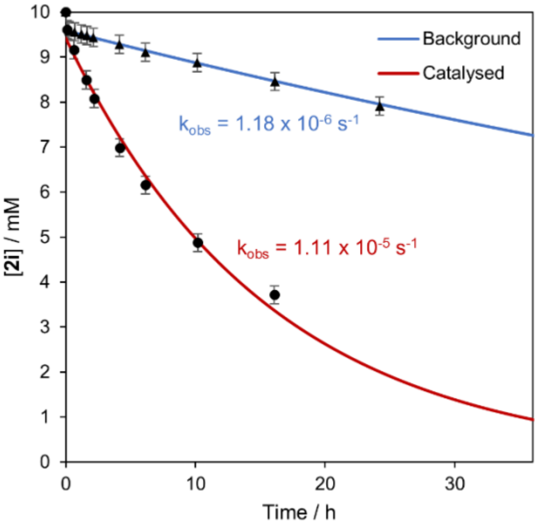

Figure S8. Kinetics of fuel consumption catalyzed by **1b.** (**A**) [Racemic (±)-**1b**] = 1.0 mM, [DMAP] = 1.0 mM, [**2h**] = 10.0 mM, [MES buffer] = 100 mM (pH_{obs} 5.10 in D_2O) CD_3CN:D_2O (7:3 *v/v*) at r.t. (**B**) Table showing the observed rate constant k_{obs}, the computed catalyzed rate constant k_{cat}, the rate enhancement factor of the consumption of fuel by the catalyst **1b**, and the half-life of the fuel for the background and catalyzed pathways pertaining to the fueling reaction shown in panel **A**. Rate enhancement corresponds to 88% of fuel molecules reacting via the machine-catalyzed pathway. (**C**) Partial ^{1}H NMR spectra (MES-buffered (100 mM, pH_{obs} 5.10) CD_3CN:D_2O (7:3 *v/v*), 600 MHz, 298 K) showing the disappearance of **2h** fuel and appearance of **3h** urea waste (CH_3 signals). (**D**) Kinetics of carbodiimide hydration in the absence and presence of **1b** (1.0 mM), determined by ^{1}H NMR spectroscopy. Solid lines represent the fit to pseudo-first order kinetics (k_{obs}).

A

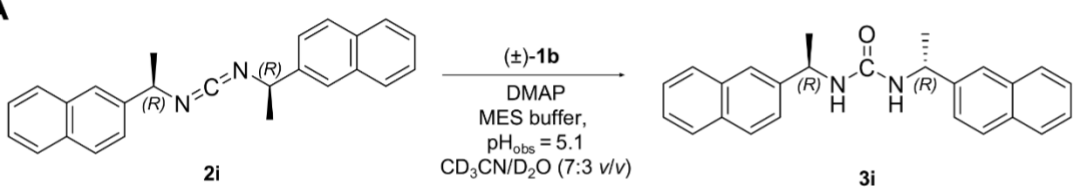

B

Reaction	k_{obs} (s^{-1})	k_{cat} (s^{-1})	Rate enhancement	$t_{1/2}$ (h)	% Fuel consumption
Background	2.14×10^{-6}	-	-	90.1	10
Catalysed	1.78×10^{-5}	1.56×10^{-5}	8.3	10.8	90

C **D**

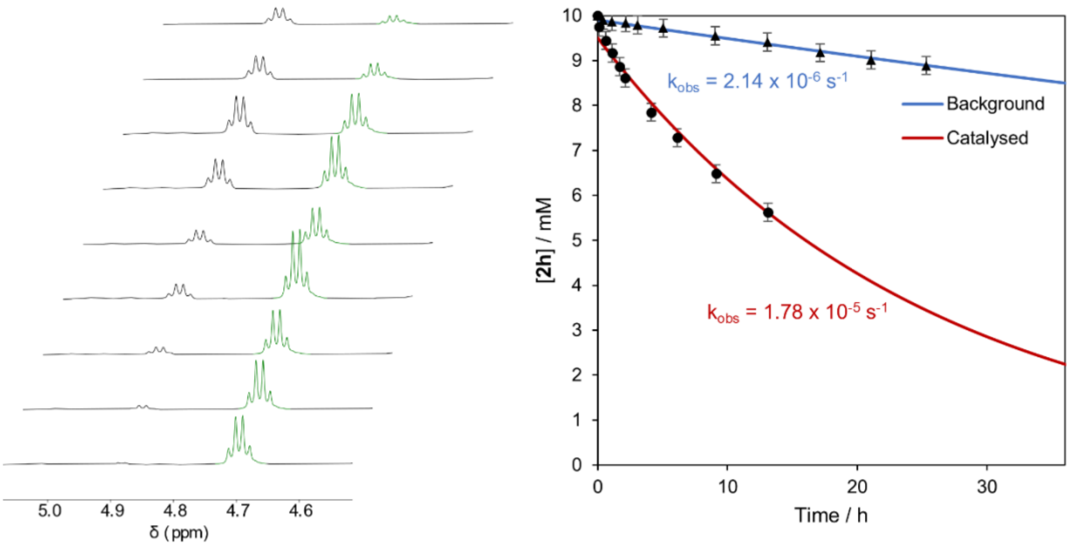

Figure S9. Kinetics of fuel consumption catalyzed by **1b.** (**A**) [Racemic (±)-**1b**] = 1.0 mM, [DMAP] = 1.0 mM, [**2i**] = 10.0 mM, [MES buffer] = 100 mM (pH_{obs} 5.10 in D_2O) CD_3CN:D_2O (7:3 *v/v*) at r.t. (**B**) Table showing the observed rate constant k_{obs}, the computed catalyzed rate constant k_{cat}, the rate enhancement factor of the consumption of fuel by the catalyst **1b**, and the half-life of the fuel for the background and catalyzed pathways pertaining to the fueling reaction shown in panel **A**. Rate enhancement corresponds to 90% of fuel molecules reacting via the machine-catalyzed pathway. (**C**) Partial ^{1}H NMR spectra (MES-buffered (100 mM, pH_{obs} 5.10) CD_3CN:D_2O (7:3 *v/v*), 600 MHz, 298 K) showing the disappearance of **2i** fuel and appearance of **3i** urea waste (CH_3 signals). (**D**) Kinetics of carbodiimide hydration in the absence and presence of **1b** (1.0 mM), determined by ^{1}H NMR spectroscopy. Solid lines represent the fit to pseudo-first order kinetics (k_{obs}).

S3.2 Monitoring fuel consumption in the presence of hydrolysis promoters 4b–d

Preparation of samples

[Racemic (±)-**1b**] = 1.0 mM, [**4b–d**] = 1.0 mM, [Fuel] = 10.0 mM, [MES buffer] = 100 mM (pH_{obs} 5.10 in D_2O) CD_3CN:D_2O (7:3 *v/v*) at r.t.

In an NMR tube, racemic (±)-**1b** (5.0 μL of a 0.1 M stock solution in DMF) was diluted into 350 μL of CD_3CN. To the solution was added 100 μL of D_2O, 100 mM of MES buffer (50 μL of a 1.0 M stock solution in D_2O, pH_{obs} 5.10), and hydrolysis promoter **4b–d** (2.0 μL of a 0.25 M stock solution in dioxane-d_8). DIC[c] or di-*tert*-butylcarbodiimide[d] fuel (25 μL of a 0.2 M stock solution in CD_3CN, 10.0 mM) was added and the reaction was monitored over time by ^{1}H NMR spectroscopy. Concentration data were obtained from the relative integrals of the ^{1}H NMR signals corresponding to the aliphatic protons of DIC[c] or di-*tert*-butylcarbodiimide[d]. The data were used to fit a line produced by a *pseudo*-first order rate equation using non-linear regression, by varying the *pseudo*-first order rate constant (k_{obs}) and the initial concentration (Figure S10).

A

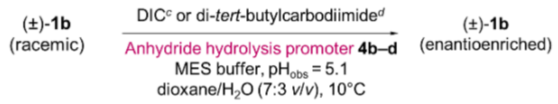

B

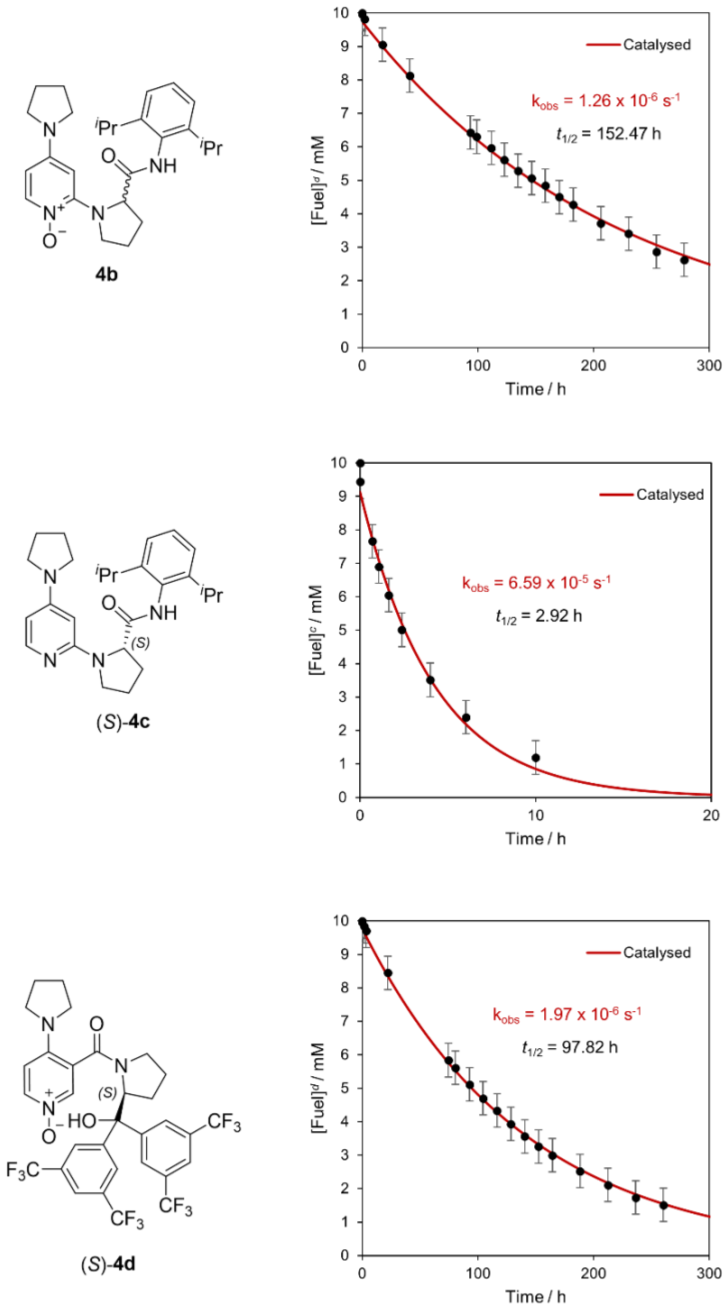

Figure S10. Kinetics of fuel consumption catalyzed by **1b** in the presence of hydrolysis promoters **4b─d**. (**A**) [Racemic (±)-**1b**] = 1.0 mM, [**4b─d**] = 1.0 mM, [c][DIC] = 10.0 Mm or [d][di-*tert*-butylcarbodiimide] = 10.0 mM, [MES buffer] = 100 mM (pH_{obs} 5.10 in D_2O) CD_3CN:D_2O (7:3 *v/v*) at r.t. (**B**) Plots showing the observed rate constant k_{obs} of the consumption of fuel by the catalyst **1b** in the presence of the respective hydrolysis promoters **4b─d**, and the half-life of the fuel pertaining to the fueling reaction shown in panel **A**. The kinetics of fuel consumption were determined by ^{1}H NMR spectroscopy. Solid lines represent the fit to pseudo-first order kinetics (k_{obs}).

S4. Single kinetic gating analysis

High-performance liquid chromatography (HPLC) was performed on an Agilent 1260 Infinity system using ChiralPak IF column. HPLC data were analyzed in Open Labs CDS software, and traces were exported as .csv data files for further plotting in Microsoft Excel. Eluent A is composed of CH_2Cl_2:*i*-PrOH:CF_3CO_2H:*n*-hexane, 66.5:3.4:0.1:30 (*v/v/v/v*) at 25 °C with a flowrate of 1 mL min^{-1}. Eluent B is composed of *i*-PrOH:CF_3CO_2H:*n*-hexane, 1.98:0.02:98 (*v/v/v*) at 25 °C with a flowrate of 2 mL min^{-1}. Traces based on the absorbances at 265 nm (≈ λ_{max} of motor **1b**) are reported. The exact ratio of the two components was adjusted depending on the other components in the mixture for analysis in order to avoid overlap of the peaks. It was also found that variations in solvent and water content of the injection solution resulted in some variation in the retention times of various compounds, hence the need for specifically tuning the HPLC conditions for each experiment to prevent peaks overlapping. Eluent mixtures A or B are reported for individual HPLC traces. Atropisomers (−)-**1b** and (+)-**1b** were assigned according to literature references.[S1]

S4.1 Assignment of atropisomers of (±)-1b to HPLC peaks

Atropisomers of (±)-**1b** were assigned according to previous publications (Figure S11).[S1]

To get accurate retention times in comparison to the fueling experiments, the HPLC sample was prepared according to fueling conditions, without the addition of fuel or hydrolysis promoter: [Racemic motor (±)-**1b**] = 1.0 mM, [MES buffer] = 100.0 mM, pH_{obs} = 5.1, dioxane:H_2O (7:3 *v/v*).

Preparation of sample

[Racemic (±)-**1b**] = 1.0 mM, [MES buffer] = 100 mM (pH_{obs} 5.10 in D_2O) dioxane/H_2O (7:3 *v/v*).

Racemic (±)-**1b** (2.5 μL of a 0.1 M stock solution in DMF) was diluted into 70 μL of dioxane. To the solution was added 20 μL of H_2O and 100 mM of MES buffer (10 μL of a 1.0 M stock solution in D_2O, pH_{obs} 5.10).

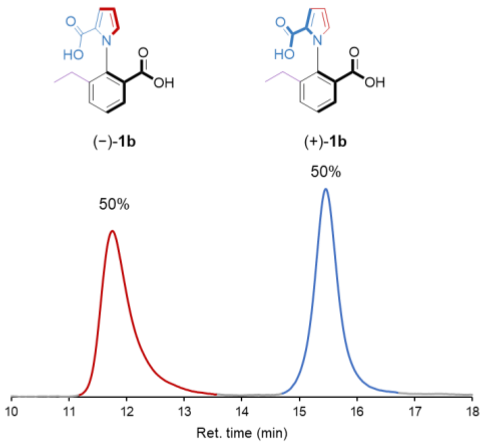

Figure S11. Chiral HPLC trace analysis (ChiralPak IF column, 25 °C, *i*-PrOH:CF_3CO_2H:*n*-hexane, 1.98:0.02:98 (*v/v/v*) 2 mL min^{-1}) of (±)-1b showing the retention times of atropisomers (−)-**1b** and (+)-**1b**.

S4.2 Enantioenrichment using fuels 2a–i

The kinetic gating of the fueling step was evaluated by applying the standard fueling conditions using each of the chiral fuels **2a–i** in separate samples along with achiral anhydride hydrolysis promoter 4-dimethylaminopyridine (DMAP). The respective directionalities and % *e.e.* are shown in Figure S12.

Preparation of samples

[Racemic (±)-**1b**] = 1.0 mM, [DMAP] = 1.0 mM, [Fuel **2a–i**] = 10.0 mM, [MES buffer] = 100 mM (pH_{obs} 5.10 in D_2O), dioxane:D_2O (7:3 *v/v*) at 10°C.

Racemic (±)-**1b** (5.0 μL of a 0.1 M stock solution in DMF) was diluted into 350 μL of dioxane. To the solution was added 100 μL of H_2O, 100 mM of MES buffer (50 μL of a 1.0 M stock solution in D_2O, pH_{obs} 5.10), and DMAP (2.5 μL of a 0.2 M stock solution in CD_3CN:D_2O, 1:1 *v/v*). Carbodiimide fuel **2a–i** (25.0 μL of a 0.2 M stock solution in dioxane, 10.0 mM) was added and the reaction was left to run at 10°C for 2 days.

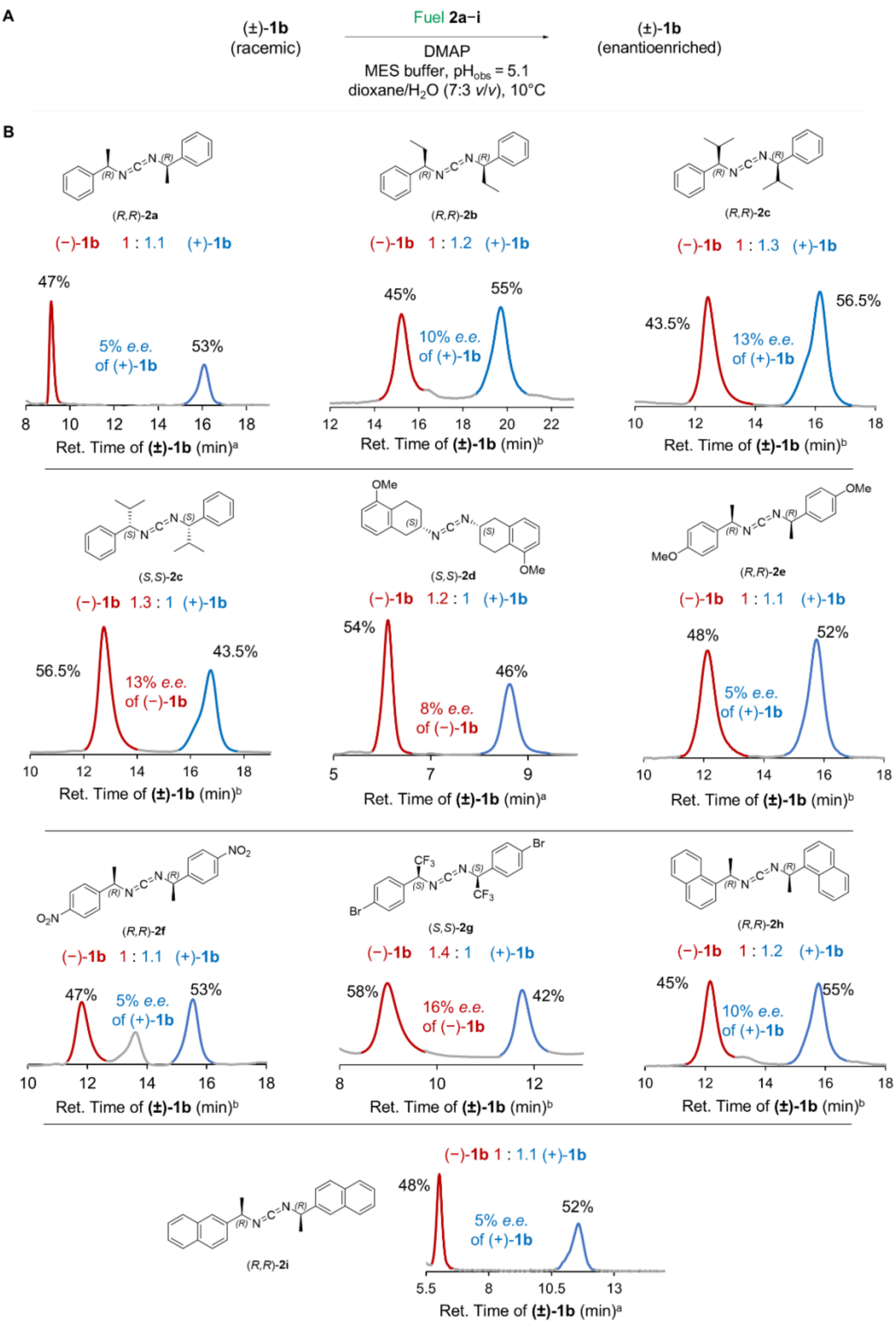

Figure S12. Enantioenrichment of **(±)-1b** using fuels **2a–i**. (**A**). General reaction scheme for the fueling reaction. [Racemic **(±)-1b**] = 1.0 mM, [DMAP] = 1.0 mM, [Fuel **2a–i**] = 10.0 mM, [MES buffer] = 100 mM (pH_{obs} 5.10 in D_2O) dioxane:H_2O (7:3 *v/v*) at 10 °C. (**B**) Chiral HPLC traces analysis (ChiralPak IF column, 25 °C, [a]CH_2Cl_2:*i*-PrOH:CF_3CO_2H:*n*-hexane, 66.5:3.4:0.1:30 (*v/v/v/v*), 1 mL min^{-1} or [b]*i*-PrOH:CF_3CO_2H:*n*-hexane, 1.98:0.02:98 (*v/v/v*), 2 mL min^{-1}) of **(±)-1b** subsequent to fueling with fuels **2a–i**. The structure of the fuel used, the resulting directionality and % *e.e.* of **(±)-1b** are shown with their respective HPLC traces.

S4.3 Enantioenrichment using hydrolysis promoters 4a–j

The kinetic gating of the anhydride hydrolysis step was evaluated by applying the standard fueling conditions using achiral fuel, either *N,N*-diisopropylcarbodiimide (DIC) or di-*tert*-butylcarbodiimide where appropriate, and each of the chiral anhydride hydrolysis promoters **4a–j** in separate samples. The respective directionalities and % *e.e.* are shown in Figure S13. We also investigated an additional DMAP and DMAP-*N*-oxide derivative developed by the Spivey group.

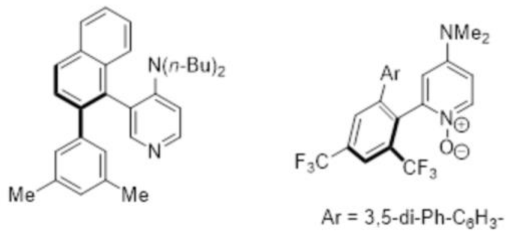

Ar = 3,5-di-Ph-C_6H_3-

Preparation of samples

[Racemic (±)-**1b**] = 1.0 mM, [**4a–j**] = 1.0 mM, [Fuel] = 10.0 mM, [MES buffer] = 100 mM (pH_{obs} 5.10 in D_2O), dioxane:H_2O (7:3 *v/v*) at 10°C.

Racemic (±)-**1b** (5.0 μL of a 0.1 M stock solution in DMF) was diluted into 350 μL of dioxane. To the solution was added 100 μL of H_2O, 100 mM of MES buffer (50 μL of a 1.0 M stock solution in D_2O, pH_{obs} 5.10), and promoter **4a–j** (2.5 μL of a 0.2 M stock solution in CD_3CN:D_2O, 1:1 *v/v*). DIC[c] or di-*tert*-butylcarbodiimide[d] fuel (25.0 μL of a 0.2 M stock solution in dioxane, 10.0 mM) was added and the reaction was left to run at 10°C for 2 days.

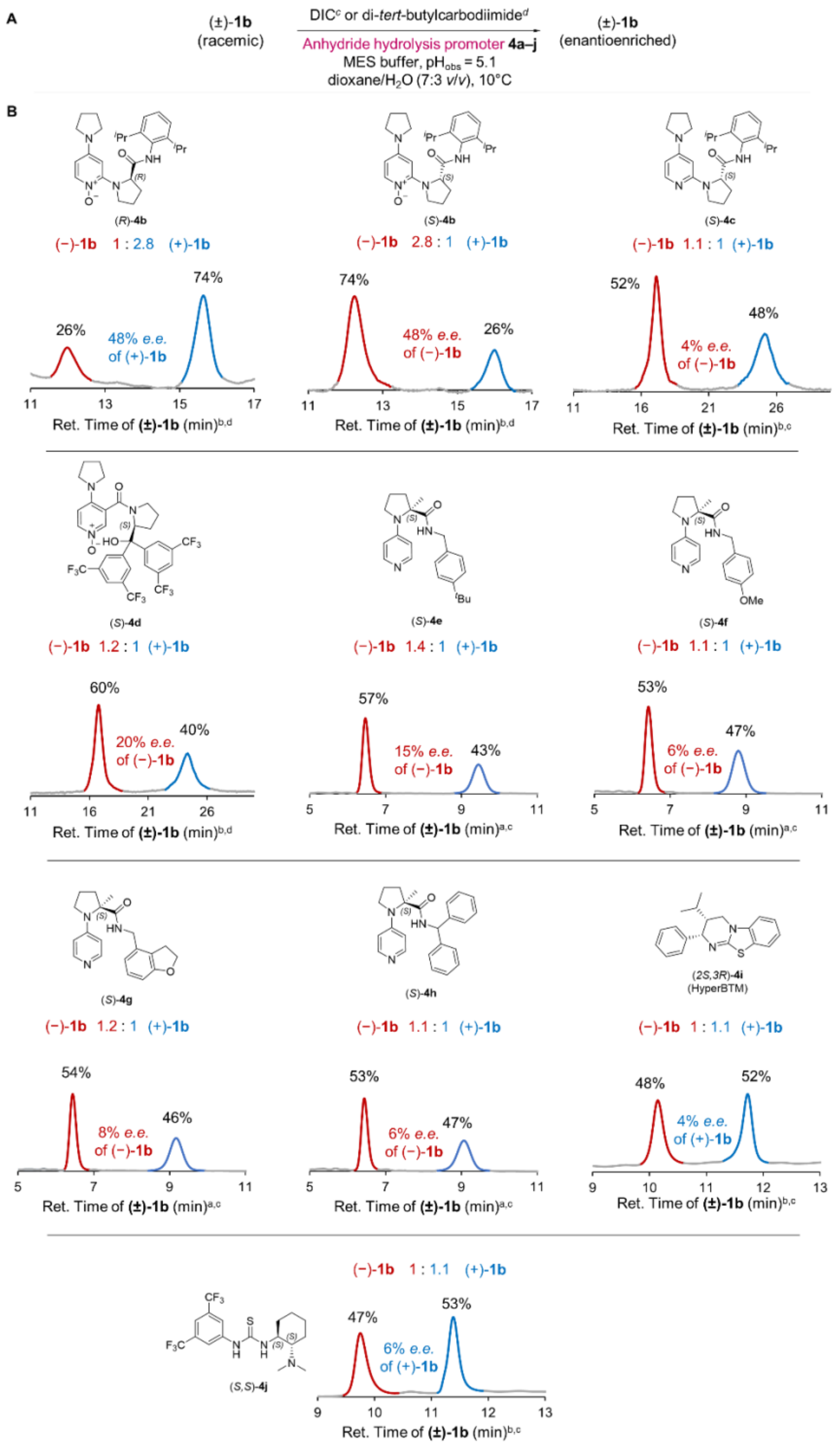

Figure S13. Enantioenrichment of **(±)-1b** using anhydride hydrolysis promoters **4a–j**. (**A**). General reaction scheme for the fueling reaction. [Racemic **(±)-1b**] = 1.0 mM, [**4a–j**] = 1.0 mM [c][DIC] = 10.0 Mm or [d][di-*tert*-butylcarbodiimide] = 10.0 mM, [MES buffer] = 100 mM (pH_{obs} 5.10 in D_2O) dioxane:H_2O (7:3 *v/v*) at 10 °C. (**B**) Chiral HPLC traces analysis (ChiralPak IF column, 25 °C, [a]CH_2Cl_2:*i*-PrOH:CF_3CO_2H:*n*-hexane, 66.5:3.4:0.1:30 (*v/v/v/v*), 1 mL min^{-1} or [b]*i*-PrOH:CF_3CO_2H:*n*-hexane, 1.98:0.02:98 (*v/v/v*), 2 mL min^{-1}) of **(±)-1b** subsequent to fueling with [c]DIC or [d]di-*tert*-butylcarbodiimide and hydrolysis promoters **4a–j**. The structure of the hydrolysis promoter used, the resulting directionality and % *e.e.* of **(±)-1b** are shown with their respective HPLC traces.

S4.4 Methods to measure kinetic gating

The kinetic biases (chemical gating) in molecular motors are best approximated using motor analogues that form stable atropisomers in the unfuelled state (diacid in this case). This way, once all motor has returned to the diacid state, the ratio between (+) and (−) atropisomers can be assessed using chiral HPLC. It is important that the gating is measured under the same reaction conditions as the motor operation, as changes in solvent, pH, ionic strength (etc.) may alter the gating, preventing valid extrapolation of the measurement.

S4.4.1 Gating of the anhydride hydrolysis step

This kinetic bias in anhydride hydrolysis, *i.e.*, the product of the chemical gating and the equilibrium constant between anhydride conformers, is found by adding an excess (at least 2 equivalents) of achiral *N,N*-diisopropylcarbodiimide (DIC) or di-*tert*-butylcarbodiimide fuel in the presence of the chosen chiral hydrolysis promotor. The excess of fuel ensures that, on average, each motor goes through the anhydride formation and hydrolysis cycle at least once, allowing the ratio of diacid atropisomers to reach a driven steady state. Once all fuel is used and all anhydride is hydrolysed, the ratio of diacid atropisomers can be measured to directly show the bias in anhydride hydrolysis.

$$\frac{k_{+H}^{(+)}}{k_{+H}^{(-)}} \approx \frac{[(+)\textbf{-1b}]_{SS}}{[(-)\textbf{-1b}]_{SS}} = \frac{p\big((+)\textbf{-1b}\big)_{SS}}{p\big((-)\textbf{-1b}\big)_{SS}} = \frac{int\big((+)\textbf{-1b}\big)}{int\big((-)\textbf{-1b}\big)} \qquad \textit{(Eq. 1)}$$

$k_{+H}^{(-)}$= rate constant for anhydride hydrolysis from (−)-**1′b**, $k_{+H}^{(+)}$= rate constant for anhydride hydrolysis from (+)-**1′b**, *p* = proportion (normalised concentration *vs* total concentration of motor **1b**), *int* = integral of the peak of (±)-**1b** measured by chiral HPLC, subscript *SS* = steady state value. Model-motor **1b** was used for the experiments.

S4.4.2 Gating of the anhydride formation step

For the motor-molecules discussed in this manuscript, formation of anhydride is always the 'slow' chemical step in the reaction cycle, making it the primary factor in determining the rate of the reaction cycle (rate limiting step assumption). As such, in the absence of chiral hydrolysis promotor to differentiate the rates of hydrolysis from

each anhydride conformer, the ratio of the driven rates of exchange between the two diacid atropisomers of the motor simplifies to the gating (ratio of rates) of anhydride formation multiplied by the proportion of each atropisomer. As with a standard equilibration (non-driven steady state), the driven steady state ratio of diacid atropisomers is reached when the ratio of rates is equal to the reciprocal ratio of the diacid species.

$$\frac{k_{+F}^{(-)}}{k_{+F}^{(+)}} \approx \frac{[(+)\textbf{-1b}]_{SS}}{[(-)\textbf{-1b}]_{SS}} = \frac{p\big((+)\textbf{-1b}\big)_{SS}}{p\big((-)\textbf{-1b}\big)_{SS}} = \frac{int\big((+)\textbf{-1b}\big)}{int\big((-)\textbf{-1b}\big)} \qquad \textit{(Eq. 2)}$$

$k_{+F}^{(-)}$= rate constant for anhydride formation from (−)-**1b**, $k_{+F}^{(+)}$= rate constant for anhydride formation from (+)-**1b**, *p* = proportion (normalised concentration *vs* total concentration of motor **1b**), *int* = integral of the peak of (±)-**1b** measured by chiral HPLC, subscript *SS* = steady state value. Model-motor **1b** was used for the experiments.

To reach the steady state ratio, an excess (at least 2 equivalents) of chiral carbodiimide fuel is added, and the reaction can be quenched (hydrolysing remaining anhydride) once steady state is reached after the motor has turned over more than one equivalent of fuel. The ratio of atropisomers can be directly assessed by chiral HPLC. This method will tend to produce an underestimation of gating as the rate of anhydride formation increases relative to the rate of hydrolysis, since more motor will remain in the non-biased anhydride state while fuel is present, thus decreasing apparent directionality.

Alternatively, gating of anhydride formation can be estimated from the ratio of unreacted acid in presence when a sub-stoichiometric amount of chiral carbodiimide fuel is used, following a typical kinetic resolution methodology. In this case the gating is found by adapting rate expression for anhydride formation, which is first order in terms of the model-motor **1b**.

$$[(-)\textbf{-1b}]_t = [(-)\textbf{-1b}]_0\, e^{-k_{+F}^{(-)} t[F]_t} \;\; so \; -k_{+F}^{(-)} t[F]_t = \ln\left(\frac{[(-)\textbf{-1b}]_t}{[(-)\textbf{-1b}]_0}\right) \qquad \textit{(Eq. 3)}$$

$k_{+F}^{(-)}$= rate constant for anhydride formation from (−)-**1b**, *t* = time, [F] = concentration of fuel, subscripts *t* = value at a given time, 0 = initial value (*t* = 0). Model-motor **1b** was used for the experiments.

The term $-t[F]$ appears in both the (−) and (+) atropisomer versions of the equations, so cancels in the ratio. Using chiral HPLC peak integrals to find concentrations, the

extent of reaction must also be known (concentration of anhydride formed), which can be measured by ^{1}H NMR directly before the reaction is stopped.

$$\frac{k_{+F}^{(-)}}{k_{+F}^{(+)}} \approx \frac{\ln\left(\frac{[(-)\text{-}\mathbf{1b}]_{SS}}{[(-)\text{-}\mathbf{1b}]_0}\right)}{\ln\left(\frac{[(+)\text{-}\mathbf{1b}]_{SS}}{[(+)\text{-}\mathbf{1b}]_0}\right)} = \frac{\ln\left(\frac{p((-)\text{-}\mathbf{1b})_t}{p((-)\text{-}\mathbf{1b})_0}\right)}{\ln\left(\frac{p((+)\text{-}\mathbf{1b})_t}{p((+)\text{-}\mathbf{1b})_0}\right)} = \frac{\ln\left(\frac{int((-)\text{-}\mathbf{1b}) \times (1 - p(\mathbf{1'b})_q)}{p((-)\text{-}\mathbf{1b})_0}\right)}{\ln\left(\frac{int((+)\text{-}\mathbf{1b}) \times (1 - p(\mathbf{1'b})_q)}{p((+)\text{-}\mathbf{1b})_0}\right)} \quad \textit{(Eq. 4)}$$

$k_{+F}^{(-)}$= rate constant for anhydride formation from (−)-**1b**, $k_{+F}^{(+)}$= rate constant for anhydride formation from (+)-**1b,** *int* = the integral of the peak of (±)-**1b** measured by chiral HPLC, *p* = proportion (normalised concentration *vs* total concentration of motor **1b**), subscripts *SS* = steady state, *t* = value at a given time, 0 = initial value (*t* = 0), *q* = value at point of quenching (*t* = *q*). Model-motor **1b** was used for the experiments.

However, care must be taken not to over-interpret the result obtain using this method. Experimentally, it is required that only the formation step takes place (so that anhydride hydrolysis doesn't replace reacted diacid), so the reaction must be performed in dry conditions. As water is a major component of the reaction solvent (30%), and it is clear that altering the solvent changes the chemical gating, the chemical gating calculated using this method should be taken as purely indicative and not used without further validation. Experimentally, an excess of carbodiimide fuel was used (to speed up the anhydride formation process) and the reaction was quenched before reaching completion, typically at conversions of 30% to 40%.

Nonetheless, it was found that the values of fueling gating determined by both methods described above were in accordance (within ± 0.1 error) for the carbodiimide fuels tested.

Table S1. Anhydride formation gating values calculated by fuelling model-motor **1b** to steady state, and by quenching the reaction after a sub-stoichiometric quantity of fuel had been consumed.

Fuel	*int*((−)-1b)	*int*((+)-1b)	Reaction extent $p(\mathbf{1'b})_q$	*p*((−)-1b)	*p*((+)-1b)	Gating (quenching)	Gating (steady state)
(*R*,*R*)-**2a**	0.49	0.51	0.36	0.32	0.32	1.1	1.1
(*R*,*R*)-**2b**	0.48	0.52	0.37	0.30	0.33	1.2	1.2
(*R*,*R*)-**2c**	0.48	0.52	0.40	0.29	0.31	1.2	1.3
(*R*,*R*)-**2e**	0.49	0.51	0.30	0.35	0.35	1.1	1.1

Experiments:

[Racemic (±)-**1b**] = 1.0 mM, [DMAP] = 2.0 mM, [Fuel] = 2.0 mM, [MOPS buffer] = 100 mM (pH_{obs} = 7.9 in D_2O) in CD_3CN:D_2O (1:1 *v/v*).

In an NMR tube, racemic (±)-**1b** (5.0 μL of a 0.1 M stock solution in DMF) was diluted into 500 μL of CD_3CN and ^1H NMR was recorded. To the solution was added 2.0 mM of chiral fuel (5.0 μL of a 0.2 M stock solution in CD_3CN) and the reaction was monitored for the first 5 minutes by ^1H NMR spectroscopy until about 30% – 40% of motor **1b** was converted to anhydride **1′b** (see Table S1 for respective proportions). Then, DMAP (10.0 μL of a 0.1 M stock solution in CD_3CN:D_2O, 1:1 *v/v*) was added followed by D_2O (400 μL) and 100 mM of MOPS buffer (100 μL of a 1.0 M stock solution in D_2O, pH_{obs} 7.9). The reaction was monitored by ^1H NMR spectroscopy until complete hydrolysis of **1′b** back into **1b**. Chiral HPLC was used to determine the ratio of atropisomers of **1b**.

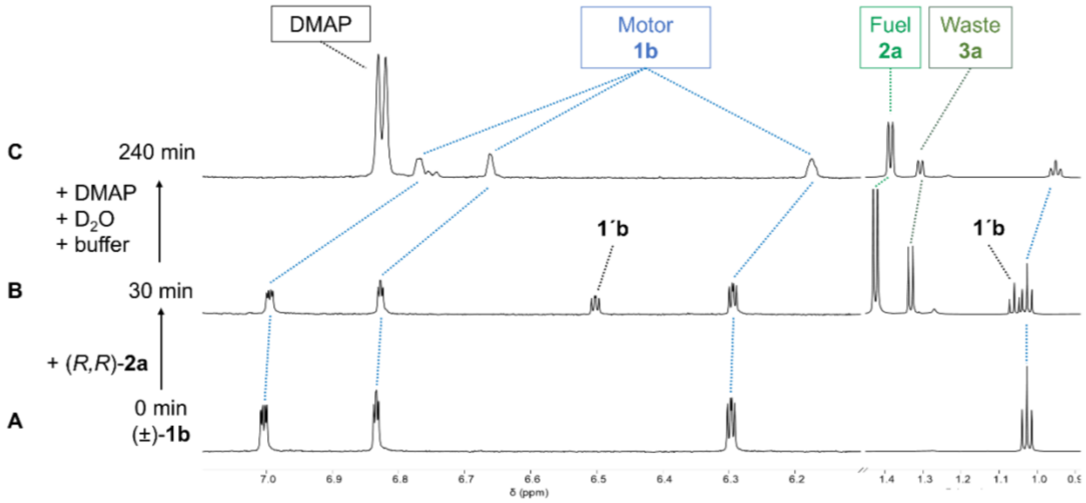

Figure S14. Singly kinetically gated stepwise anhydride formation and hydrolysis of **1b** using chiral fuel (*R,R*)-**2a**. Partial ^1H NMR spectra (CD_3CN, 600 MHz, 298 K) of the stepwise operation. The region 6.1–7.1 ppm is scaled vertically 15× compared to region 0.9–1.5 ppm. (**A**) Racemic **1b** in CD_3CN ([**1b**] = 1.0 mM). (**B**) 30 min after addition of fuel (*R,R*)-**2a** (2 equiv., [**2a**] = 2.0 mM), which reacts with **1b** to form anhydride **1′b** in 36% and urea waste. (**C**) 240 min after addition of DMAP as an achiral anhydride hydrolysis promoter (2 equiv., [DMAP] = 2.0 mM), D_2O (50% *v/v*), basic buffer ([MOPS buffer] = 100.0 mM, pH_{obs} = 7.9) to unselectively hydrolyze anhydride **1′b** back to **1b**. The basic buffer is to quench the reaction of the fuel-to-waste reaction to be able to compute the kinetic gating of the fueling step (see Figure S18A).

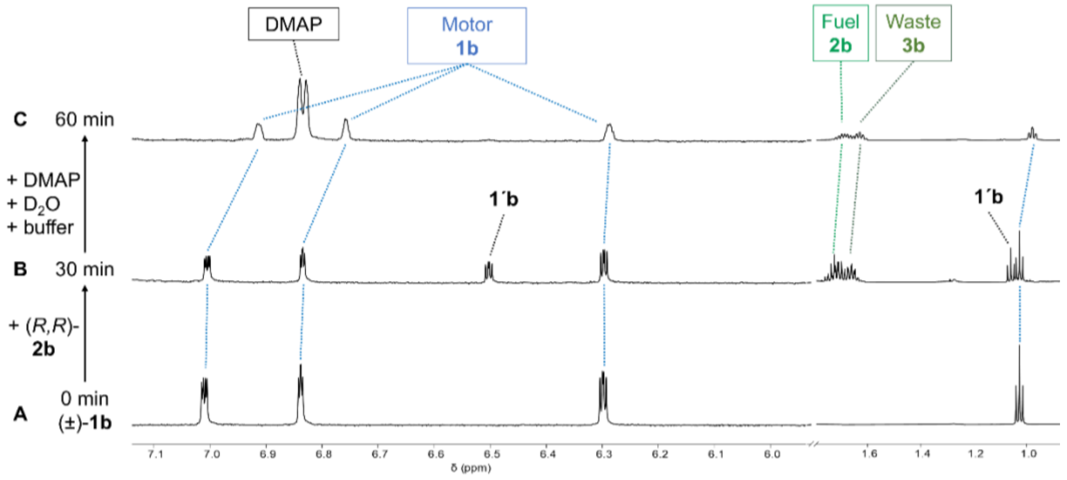

Figure S15. Singly kinetically gated stepwise anhydride formation and hydrolysis of **1b** using chiral fuel (*R*,*R*)-**2b**. Partial ^{1}H NMR spectra (CD_3CN, 600 MHz, 298 K) of the stepwise operation. The region 6.0–7.1 ppm is scaled vertically 15× compared to region 0.9–1.7 ppm. (**A**) Racemic **1b** in CD_3CN ([**1b**] = 1.0 mM). (**B**) 30 min after addition of fuel (*R*,*R*)-**2b** (2 equiv., [**2b**] = 2.0 mM), which reacts with **1b** to form anhydride **1′b** in 37% and urea waste. (**C**) 60 min after addition of DMAP as an achiral anhydride hydrolysis promoter (2 equiv., [DMAP] = 2.0 mM), D_2O (50% *v/v*), basic buffer ([MOPS buffer] = 100.0 mM, pH_{obs} = 7.9) to unselectively hydrolyse anhydride **1′b** back to **1b**. The basic buffer is to quench the reaction of the fuel-to-waste reaction to be able to compute the kinetic gating of the fueling step (see Figure S18B).

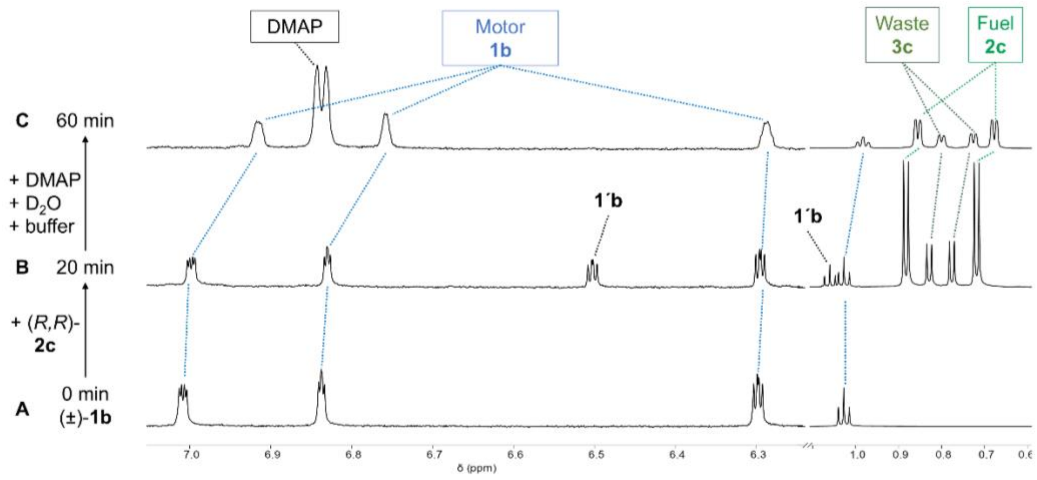

Figure S16. Singly kinetically gated stepwise anhydride formation and hydrolysis of **1b** using chiral fuel (*R*,*R*)-**2c**. Partial ^{1}H NMR spectra (CD_3CN, 600 MHz, 298 K) of the stepwise operation. The region 6.0–7.1 ppm is scaled vertically 15× compared to region 0.8–1.1 ppm. (**A**) Racemic **1b** in CD_3CN ([**1b**] = 1.0 mM). (**B**) 20 min after addition of fuel (*R*,*R*)-**2c** (2 equiv., [**2c**] = 2.0 mM), which reacts with **1b** to form anhydride **1′b** in 40% and urea waste. (**C**) 60 min after addition of DMAP as an achiral anhydride hydrolysis promoter (2 equiv., [DMAP] = 2.0 mM), D_2O (50% *v/v*), basic buffer ([MOPS buffer] = 100.0 mM, pH_{obs} = 7.9) to unselectively hydrolyse anhydride **1′b** back to **1b**. The basic buffer is to quench the reaction of the fuel-to-waste reaction to be able to compute the kinetic gating of the fueling step (see Figure S18C).

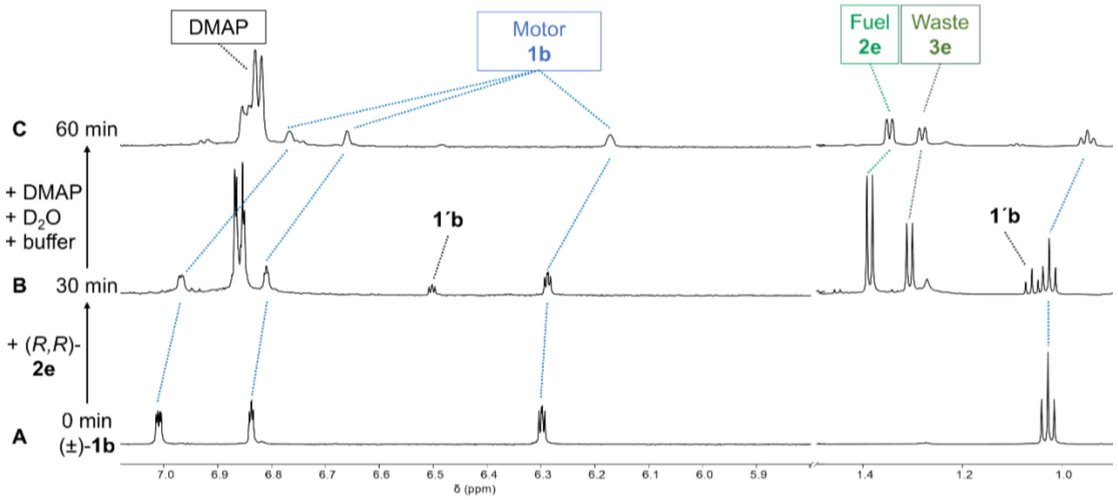

Figure S17. Singly kinetically gated stepwise anhydride formation and hydrolysis of **1b** using chiral fuel (*R,R*)-**2e**. Partial ^{1}H NMR spectra (CD_3CN, 600 MHz, 298 K) of the stepwise operation. The region 6.0–7.1 ppm is scaled vertically 15× compared to region 0.8–1.1 ppm. (**A**) Racemic **1b** in CD_3CN ([**1b**] = 1.0 mM). (**B**) 30 min after addition of fuel (*R,R*)-**2c** (2 equiv., [**2c**] = 2.0 mM), which reacts with **1b** to form anhydride **1′b** in 30% and urea waste. (**C**) 60 min after addition of DMAP as an achiral anhydride hydrolysis promoter (2 equiv., [DMAP] = 2.0 mM), D_2O (50% *v/v*), basic buffer ([MOPS buffer] = 100.0 mM, pH_{obs} = 7.9) to unselectively hydrolyse anhydride **1′b** back to **1b**. The basic buffer is to quench the reaction of the fuel-to-waste reaction to be able to compute the kinetic gating of the fueling step (see Figure S18D).

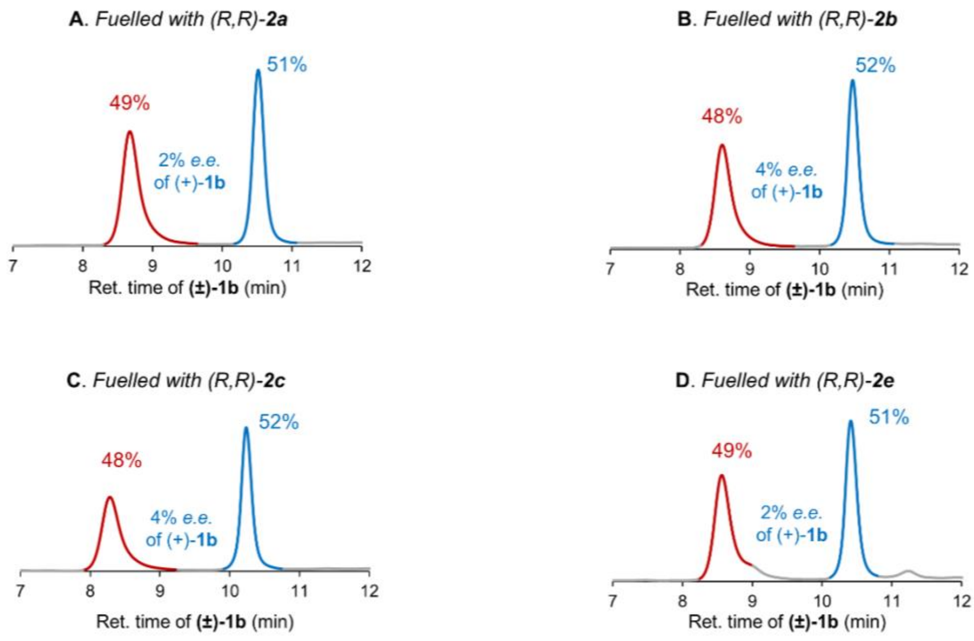

Figure S18. Chiral HPLC analysis (ChiralPak IF column, 25 °C, *i*-PrOH:CF_3CO_2H:*n*-hexane, 1.98:0.02:98 (*v/v/v*), 2 mL min^{-1}) after the sub-stoichiometric anhydride formation and hydrolysis of motor **1b** using (**A**) (*R,R*)-**2a** as shown in Figure S14, (**B**) (*R,R*)-**2b** as shown in Figure S15, (**C**) (*R,R*)-**2c** as shown in Figure S16, and (**D**) (*R,R*)-**2e** as shown in Figure S17.

S5. Optimization of the hydrolysis kinetic gating

S5.1 Optimizations for hydrolysis promoters 4b

After having examined the effect of the kinetic gating of the anhydride hydrolysis promoters **4a–j**, we selected compound **4b** as an optimal candidate for improving directionality. We proceeded by examining the fueling reaction periodically by ^{1}H NMR. We found that, in the presence of DIC, a side product formed (Figure S19A, see section S8 for details) which affected the recovery of motor **1** and the accuracy of the measurement of directionality and % *e.e.* To mitigate this effect, we used di-*tert*-butylcarbodiimide as a bulkier fuel instead of DIC. The fueling reaction was clean, and no side product or oligomers were formed (Figure S19B). The spectra of ^{1}H NMR also showed the emergence of a relatively long-lived hydrolysis intermediate, corresponding to an *N*-oxide ester (see section S8 for details). The results also show that the mechanical steady-state for motor **1b** under the re-optimized conditions is reached at 10 equivalents of fuel (Table S2, entries 3–6).

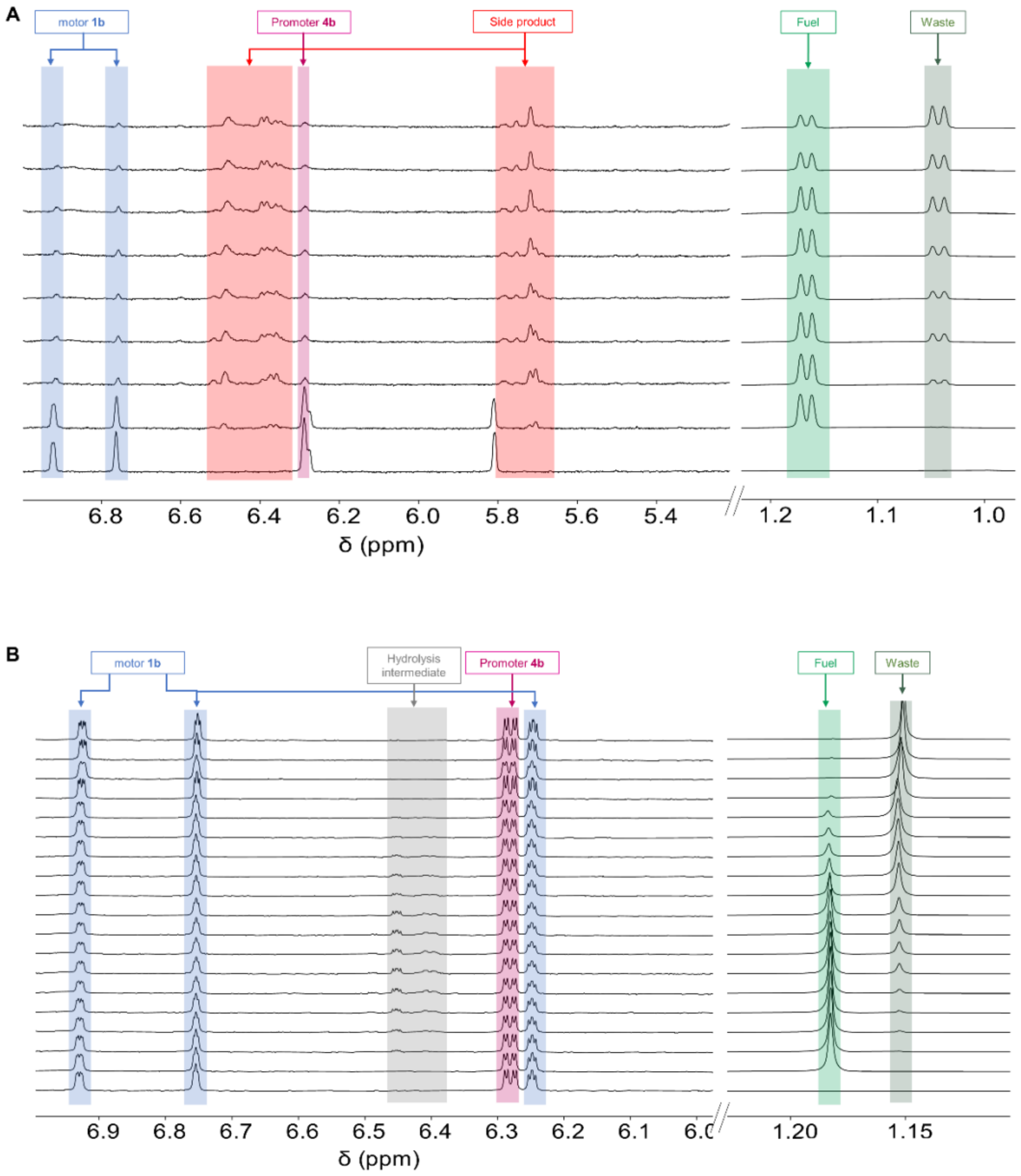

Figure S19. Partial ^{1}H NMR spectra of the fueling reaction of motor **1b**. (**A**) Using DIC as fuel, the spectra show the formation of side products (*N*-acyl urea) and poor regeneration of motor **1b** ([Racemic (±)-**1b**] = 1.0 mM, [**4b**] = 1.0 mM, [DIC] = 10.0 mM, [MES buffer] = 100 mM (pH_{obs} 5.10 in D_2O) CD_3CN/D_2O (7:3 *v/v*) at r.t.). (**B**) Using di-*tert*-butylcarbodiimide, the spectra show a clean fueling reaction with regeneration of motor **1b** and no formation of side products. ([Racemic (±)-**1b**] = 1.0 mM, [**4b**] = 2.0 mM, [di-*tert*-butylcarbodiimide] = 10.0 mM, [MES buffer] = 100 mM (pH_{obs} 5.10 in D_2O) dioxane-d_8:D_2O (1:1 *v/v*) at r.t.).

Table S2. Optimized conditions for the fueling of motor **1b** using promoter **4b**.

Entry	[Motor 1b]	[(*S*)-4b]	[DIC]	[di-*tert*-butylcarbodiimide]	Solvent (dioxane:H_2O)	[MES buffer] (pH_{obs} = 5.1)	% *e.e.* of (−)-1b	Directionality (−)-1b : (+)-1b
1	1 mM	1 mM	10 mM	0 mM	7:3	100 mM	-	-
2	1 mM	1mM	0 mM	10 mM	7:3	100 mM	48	2.8 : 1
3	1 mM	2mM	0 mM	5 mM	1:1	100 mM	86	13.2 : 1
4	1 mM	2mM	0 mM	10 mM	1:1	100 mM	90	18.7 : 1
5	1 mM	2mM	0 mM	20 mM	1:1	100 mM	90	18.7 : 1
6	1 mM	2mM	0 mM	30 mM	1:1	100 mM	90	18.7 : 1

S5.2 Enantioenrichment using hydrolysis promoters 4a–d under the re-optimized conditions

The kinetic gating of the anhydride hydrolysis step was evaluated by applying the re-optimized fueling conditions (Table S2, entry 4) using achiral fuel (di-*tert*-butylcarbodiimide) and each of the chiral anhydride hydrolysis promoters **4a**–**d** in separate samples. The respective directionalities and % *e.e* are shown in Figure S20.

Preparation of samples

[Racemic (±)-**1b**] = 1.0 mM, [**4a–d**] = 2.0 mM, [di-*tert*-butylcarbodiimide] = 10.0 mM, [MES buffer] = 100 mM (pH_{obs} 5.10 in D_2O) dioxane-d_8:D_2O (1:1 *v/v*) at r.t.

Aliquots (20.0 μL) were taken from the NMR tubes used to monitor fuel consumption in the presence of each of the hydrolysis promoters **4a–d** (see Section S5.3 for details).

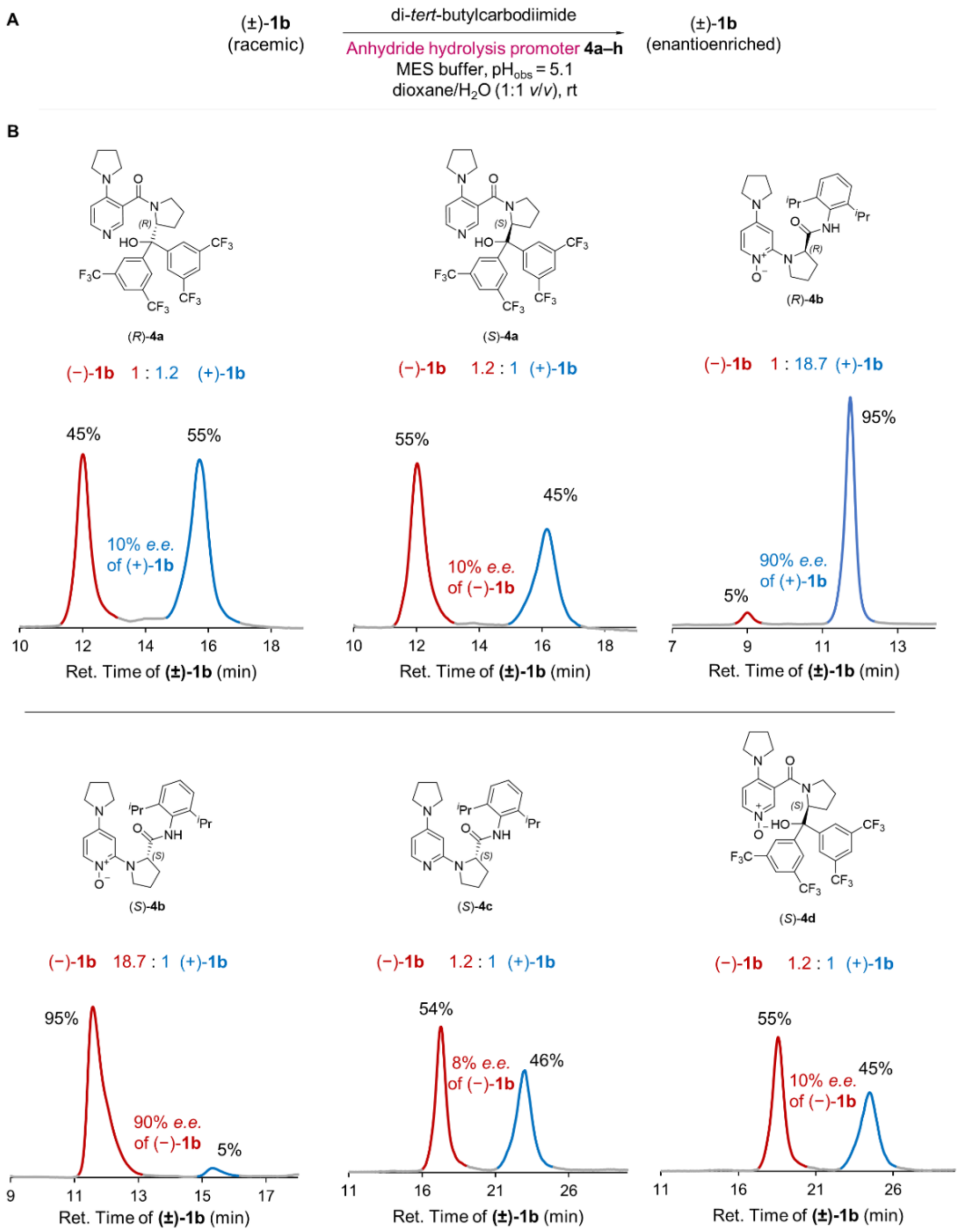

Figure S20. Chiral HPLC traces analysis (ChiralPak IF column, 25 °C, *i*-PrOH:CF_3CO_2H:*n*-hexane, 1.98:0.02:98 (*v/v/v*), 2 mL min^{-1}) of **(±)-1b** subsequent to fueling under re-optimized conditions with di-*tert*-butylcarbodiimide and hydrolysis promoters **4a–d**. [Racemic **(±)-1b**] = 1.0 mM, [**4a–d**] = 2.0 mM, [di-*tert*-butylcarbodiimide] = 10.0 mM, [MES buffer] = 100 mM (pH_{obs} 5.10 in D_2O) dioxane-d_8:D_2O (1:1 *v/v*) at r.t. The structure of the hydrolysis promoter used, the resulting directionality and % *e.e.* of **(±)-1b** are shown with their respective HPLC traces.

S5.3 Monitoring fuel consumption in the presence of hydrolysis promoters 4a–d under the re-optimized conditions

[Racemic (±)-**1b**] = 1.0 mM, [**4a–d**] = 2.0 mM, [di-*tert*-butylcarbodiimide] = 10.0 mM, [MES buffer] = 100 mM (pH_{obs} 5.10 in D_2O) dioxane-d_8:D_2O (1:1 *v/v*) at r.t.

In an NMR tube, racemic (±)-**1b** (5.0 μL of a 0.1 M stock solution in DMF) was diluted into 250 μL of dioxane-d_8. To the solution was added 180 μL of D_2O, 100 mM of MES buffer (50 μL of a 1.0 M stock solution in D_2O, pH_{obs} 5.10), and promoter **4a–d** (4.0 μL a 0.25 M stock solution in dioxane-d_8). Di-*tert*-butylcarbodiimide fuel (25 μL of a 0.2 M stock solution in dioxane-d_8, 10.0 mM) was added and the reaction was monitored over time by ^{1}H NMR spectroscopy. Concentration data were obtained from the relative integrals of the ^{1}H NMR signals corresponding to the aliphatic protons of di-*tert*-butylcarbodiimide. The data were used to fit a line produced by a *pseudo*-first order rate equation using non-linear regression, by varying the *pseudo*-first order rate constant (k_{obs}) and the initial concentration (Figure S21).

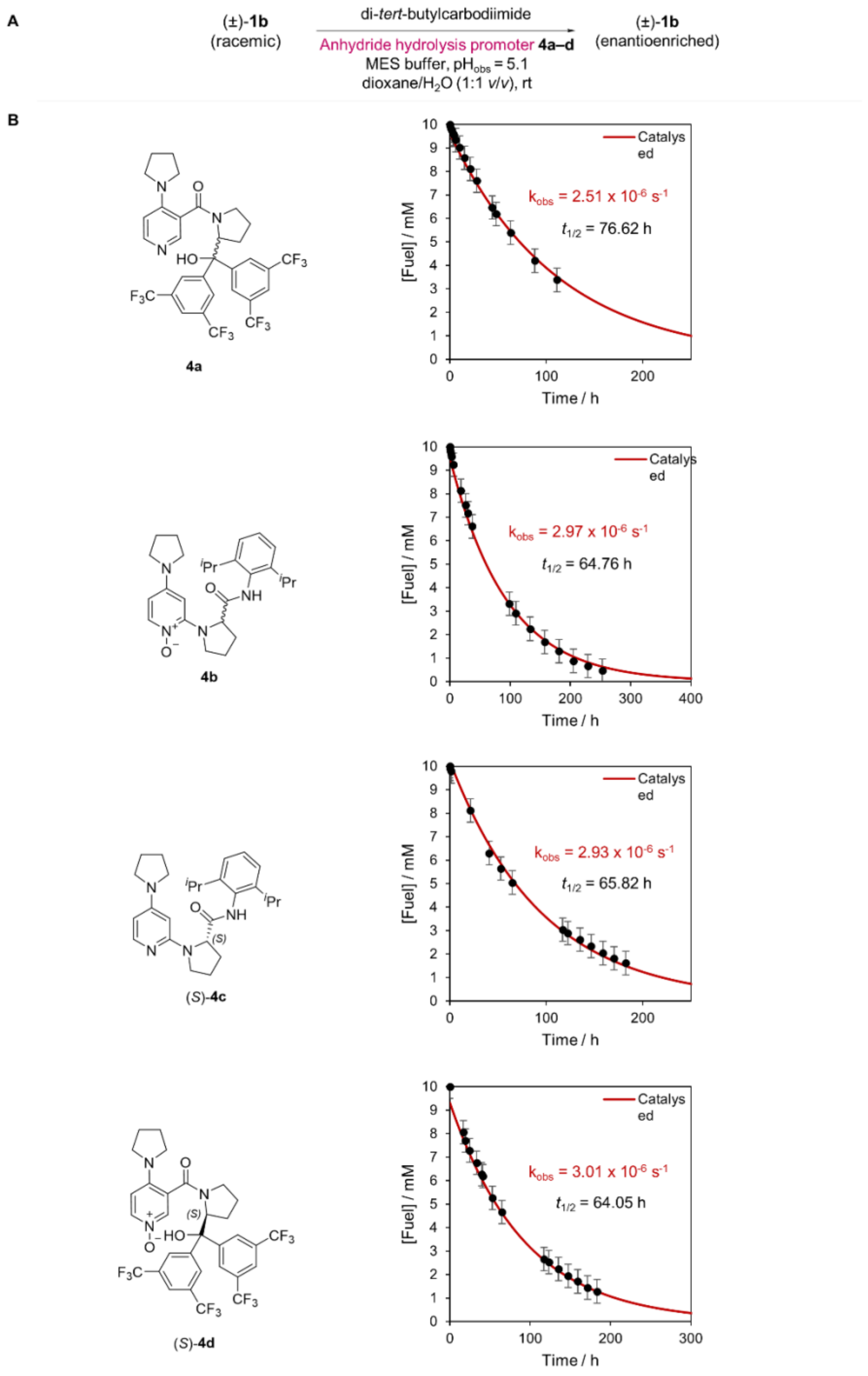

Figure S21. Kinetics of fuel consumption catalyzed by **1b** in the presence of hydrolysis promoters **4a–d** under the re-optimized conditions. (**A**) [Racemic (±)-**1b**] = 1.0 mM, [**4a–d**] = 2.0 mM, [di-*tert*-butylcarbodiimide] = 10.0 mM, [MES buffer] = 100 mM (pH_{obs} 5.10 in D_2O) dioxane-d_8:D_2O (1:1 *v/v*) at r.t. (**B**) Plots showing the observed rate constant k_{obs} of the consumption of fuel by the catalyst **1b** in the presence of the respective hydrolysis promoters **4a–d**, and the half-life of the fuel pertaining to the fueling reaction shown in panel **A**. The kinetics of fuel consumption were determined by ^{1}H NMR spectroscopy. Solid lines represent the fit to *pseudo*-first order kinetics (k_{obs}).

S6. Double kinetic gating analysis

S6.1 Kinetic gating of 2c under the re-optimized conditions of 4b

Combining hydrolysis promoter **4b** with fuel **2c** under the re-optimized conditions would introduce double kinetic gating to the fueling cycle of motor **1** and hence improve the directionality. We first fueled motor **1b** with carbodiimide **2c** and achiral DMAP under the re-optimized conditions of hydrolysis promoter **4b** (see Section S5.1, Table S2 for details) to get the individual gating of fuel **2c** under those conditions. The directionality was unimproved. Using (*S,S*)-**2c**, the directionality obtained was 1.25:1, 10% *e.e.* of (−)-**1b** (Figure S22A), while using the opposite handedness of fuel, (*R,R*)-**2c**, generated an equal and opposite *e.e.* in **1b** (Figure S22B).

Preparation of samples

[Racemic (±)-**1b**] = 1.0 mM, [DMAP] = 2.0 mM, [**2c**] = 10.0 mM, [MES buffer] = 100 mM (pH_{obs} 5.10 in D_2O), dioxane-d_8:D_2O (1:1 *v/v*) at r.t.

Racemic (±)-**1b** (5.0 μL of a 0.1 M stock solution in DMF) was diluted into 250 μL of dioxane-d_8. To the solution was added 180 μL of D_2O, 100 mM of MES buffer (50 μL of a 1.0 M stock solution in D_2O, pH_{obs} 5.10), and DMAP (5.0 μL of a 0.2 M stock solution in CD_3CN:D_2O, 1:1 *v/v*). Fuel **2c** (25.0 μL of a 0.2 M stock solution in dioxane, 10.0 mM) was added and the reaction was left to run at r.t. for 2 days.

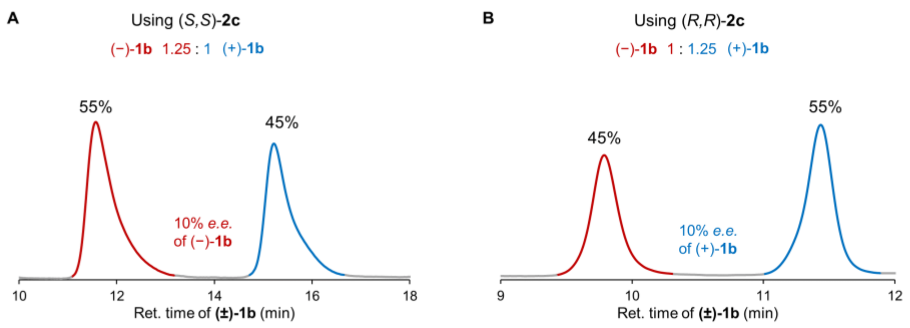

Figure S22. Chiral HPLC traces analysis (ChiralPak IF column, 25 °C, *i*-PrOH:CF_3CO_2H:*n*-hexane, 1.98:0.02:98 (*v/v/v*), 2 mL min^{-1}) of **(±)-1b** subsequent to fueling with (**A**) (*S,S*)-**2c** and (**B**) (*R,R*)-**2c** in the presence of achiral DMAP under re-optimized conditions. [**Racemic (±)-1b**] = 1.0 mM, [DMAP] = 2.0 mM, [**2c**] = 10.0 mM, [MES buffer] = 100 mM (pH_{obs} 5.10 in D_2O) dioxane-d_8:D_2O (1:1 *v/v*) at r.t.

S6.2 Batch-fueling experiment

After having established the individual kinetic gating of chiral carbodiimide **2c** under the re-optimized conditions, we proceeded by fueling motor **1b** with (*S,S*)-**2c** and hydrolysis promoter (*S*)-**4b** under those same conditions, with **2c** added portion-wise to avoid side reactions. After 8.0 mM of **2c** were consumed (8 equivalents), directionality reached 21.7:1 (91% *e.e.* of (−)-**1b**) (Figure S23). After a third portion of 4 equivalents of fuel were added, the recovery of motor **1b** was too low to calculate the directionality. The occurrence of side reactions (*N*-acyl urea formation, Figure S25) meant that batch-fueling in the presence of *N*-oxide **4b** was unreliable (see Section S8.1 for details).

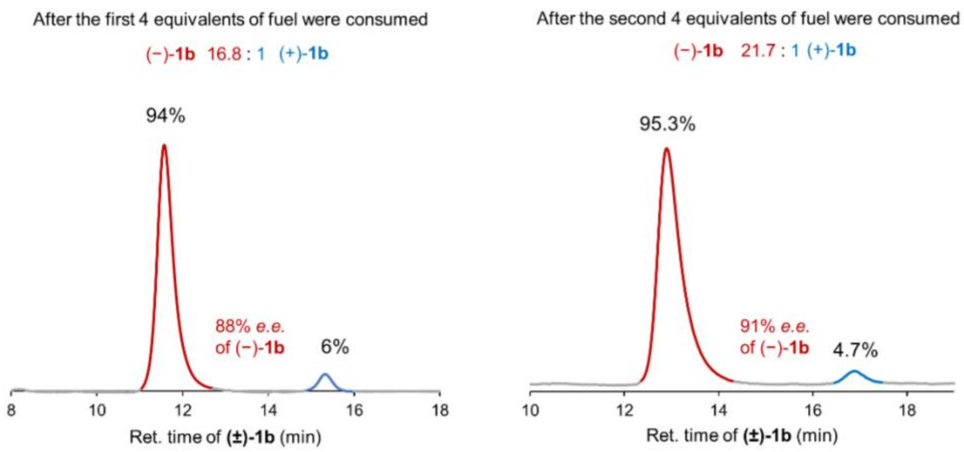

Figure S23. Chiral HPLC traces analysis (ChiralPak IF column, 25 °C, *i*-PrOH:CF_3CO_2H:*n*-hexane, 1.98:0.02:98 (*v/v/v*), 2 mL min^{-1}) of **(±)-1b** subsequent to fueling with **2c** and **4b** under re-optimized conditions. [**Racemic (±)-1b**] = 1.0 mM, [(*S*)-**4b**] = 2.0 mM, [**2c**] = 8.0 mM (added portion-wise), [MES buffer] = 100 mM (pH_{obs} 5.10 in D_2O) dioxane-d_8:D_2O (1:1 *v/v*) at r.t.

S6.3 Chemostating experiment

To avoid the formation of undesired side product (see Sections S6.2 and S8 for details), and to ensure a steady state regime for the fueling of motor **1b**, we performed a chemostating experiment. Maintaining a constant concentration of fuel by continual addition renders the system effectively chemostated, as [fuel] and [H_2O] do not change. The supply of fuel also had to be slow enough to allow for the hydrolysis intermediate to hydrolyze and avoid *N*-acyl urea formation (see Section S8 for details). Fuel **2c** was maintained at a low concentration ($\leq$ 1 mM) by slow and constant supply to the reaction mixture containing motor **1b** and chirality-matched hydrolysis promoter **4b**, and 50 μL aliquots were taken at specific time intervals to monitor the evolution of directionality with the amount of fuel added (Tables S3-4). The *e.e* plateaued at ~92%, giving a directionality of 23.5:1 after about 4.5 equivalents of fuel were supplied/consumed in both the clockwise and counterclockwise directions (Figure S24). The final ^{1}H NMR showed no formation of *N*-acyl urea (Figure S25).

Experiment:

In a round bottom flask were added 1.0 mL of dioxane-d_8, 0.8 mL D_2O, and 0.2 mL (100 mM) of MES buffer solution (1.0 M in D_2O). To the stirred mixture, 2.0 mM of **4b** were added along with 1.0 mM of motor **1b**. A syringe pump was loaded with a 10.0 mM solution of fuel **2c** in dioxane-d_8 and injected into the reaction mixture at a rate of 10.0 μL/h for ~90 hours. To measure directionality, aliquots of 50.0 μL were taken at the specified time intervals and examine via HPLC (ChiralPak IF column, 25 °C, *i*-PrOH:CF_3CO_2H:*n*-hexane, 1.98:0.02:98 (*v/v/v*), 2 mL min^{-1}).

Table S3. Table showing the data for each of the aliquots taken from the chemostating experiment using fuel (*S*,*S*)-**2c** and (*S*)-**4b** achieving counterclockwise rotation.

Aliquot	Time /h	Volume of syringe injected /μL	[2c] /mM	Directionality	% *e.e.* of (−)-1b
1	0	0	0	1 : 1	0
2	18.3	183	0.92	2.4 : 1	41.7
3	40.7	407	2.04	9.1 : 1	80.2
4	64.9	649	3.25	20.3 : 1	90.6
5	90.2	902	4.51	23.5 : 1	91.8

Table S4. Table showing the data for each of the aliquots taken from the chemostating experiment using fuel (*R*,*R*)-**2c** and (*R*)-**4b** achieving clockwise rotation.

Aliquot	Time /h	Volume of syringe injected /μL	[2c] /mM	Directionality	% *e.e.* of (−)-1b
1	0	0	0	1 : 1	0
2	16.8	168	0.84	1 : 2.7	45.3
3	45.5	455	2.28	1 : 12.6	85.3
4	69.2	692	3.46	1 : 20.9	90.9
5	73.2	732	3.66	1 : 21.7	91.2
6	89.9	899	4.49	1 : 23.5	91.8

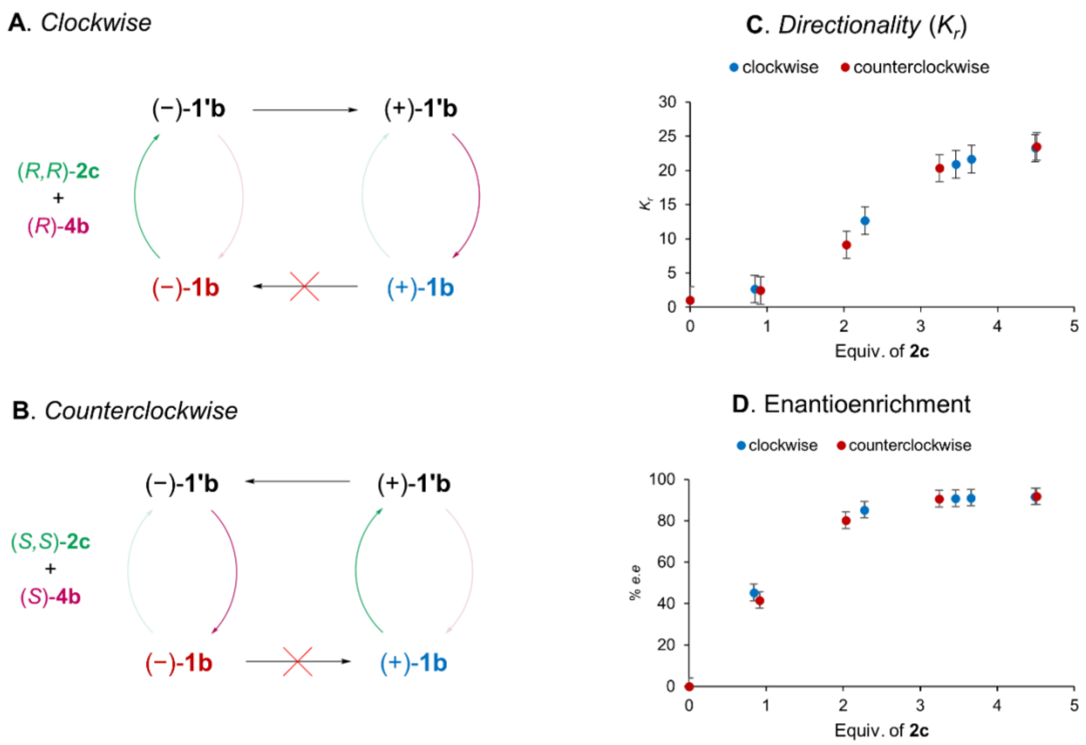

Figure S24. (**A**) Clockwise rotation of motor **1b** using (*R*,*R*)-**2c** and (*R*)-**4b**. (**B**) Counterclockwise rotation of motor **1b** using (*S*,*S*)-**2c** and (*S*)-**4b**. (**C**). Plotting the directionality obtained from the chemostating experiments demonstrates a directional rotation of ~24:1 in both the clockwise (using (*R*,*R*)-**2c** and (*R*)-**4b**) and counterclockwise (using (*S*,*S*)-**2c** and (*S*)-**4b**) directions. (**D**) Plotting the % *e.e.* of (±)-**1b** resulting from the chemostating experiments shows increase and pleau-ing of the % *e.e.*.

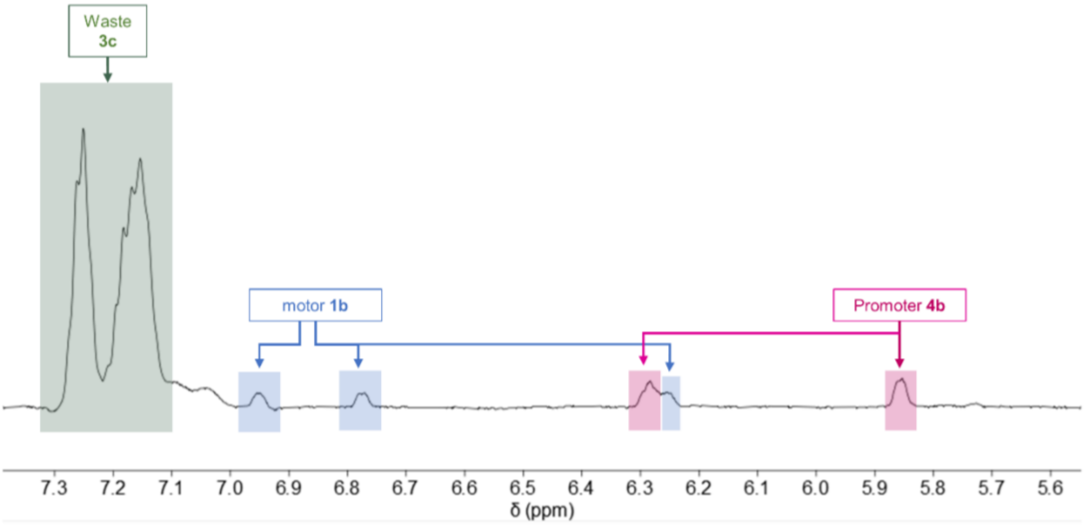

Figure S25. Partial ^{1}H NMR spectra subsequent to the chemostated fueling experiment of motor **1b**. Using **2c** as fuel at constant low constant concentration, the spectrum shows no formation of *N*-acyl urea and good motor recovery.

S7. Completing 360° rotation using motor–molecule 1c

In order to achieve the full 360° rotation of the motor, motor-molecule **1c** which has a relatively slow rotation past its Cl-substituent was used. Firstly, to demonstrate the kinetic gating of the fueling step, fuel **2c** was added to a solution of (±)-**1c** in CD_3CN (Figure S26A). After about 35% conversion of **1c** to **1′c** (5 mins), the reaction was quenched with basic buffer solution (MOPS buffer, pH_{obs} = 7.9) and achiral DMAP to hydrolyse **1′c** unselectively over 180 min (Figure S26D). Chiral HPLC showed 10% e.e. of (+)-**1′c**, representing the contribution of fuel **2c** in the stepwise operation (Figure S29B). Analogously, the same experiment was performed using (*R*)-**4b** instead of DMAP to selectively hydrolyse anhydride **1′c** (Figure S27). To make sure all of the formed anhydride **1′c** was selectively ring-opened, hydrolysis promoter (*R*)-**4b** was added prior to the addition of the aqueous buffer solution. Chiral HPLC showed 54% e.e. of (+)-**1′c**, representing the contributions of both fuel **2c** and **4b** in the stepwise operation (Figure S29C). Heating of the doubly kinetically gated experiment allowed motor **1c** to racemize past the Cl-substituent, demonstrating the full 360° rotation (Figure 29E). Heating under basic conditions resulted in a motor–promoter biproduct forming (Figure S27E). To avoid this issue, the experiment was repeated and motor **1c** was isolated prior to heating (Figure S28).

[Racemic (±)-**1c**] = 1.0 mM, [DMAP or (*R*)-**4b**] = 2.0 mM, [(*R,R*)-**2c**] = 2.0 mM, [MOPS buffer] = 100 mM (pH_{obs} = 7.9 in D_2O) in CD_3CN:D_2O (1:1 *v/v*).

Experiment:

In an NMR tube, racemic (±)-**1c** (5.0 μL of a 0.1 M stock solution in CD_3CN) was diluted into 500 μL of CD_3CN and ^{1}H NMR and chiral HPLC traces were recorded. To the solution was added 2.0 mM of (*R,R*)-**2c** (5.0 μL of a 0.2 M stock solution in CD_3CN) and the reaction was monitored for the first 5 minutes by ^{1}H NMR spectroscopy until about 35% of motor **1c** was converted to anhydride **1′c**. Then, DMAP (10.0 μL of a 0.1 M stock solution in CD_3CN:D_2O, 1:1 *v/v*) or (*R*)-**4b** (4.0 μL of a 0.25 M stock solution

in DMF) was added followed by D_2O (400 μL) and 100 mM of MOPS buffer (100 μL of a 1.0 M stock solution in D_2O, pH_{obs} 7.9). The reaction was monitored by ^1H NMR spectroscopy until complete hydrolysis of **1′c** back into **1c**. Chiral HPLC was used to determine the ratio of enantiomers of **1c**. The sample was subsequently heated at 85°C for 19 hours and ^1H NMR showed the formation of a motor–promoter byproduct, likely due to heating under basic conditions.

A similar experiment was subjected to work up in order to isolate motor **1c** prior to heating to avoid side reactions. After complete hydrolysis of anhydride **1′c** by D_2O and **4b**, the solution was diluted with H_2O and washed with ethyl acetate. The aqueous layer (containing motor **1c**) was then acidified with 1M HCl ethyl acetate solution and extracted with ethyl acetate. The organic layer was collected, the solvent was evaporated under reduced pressure (the *% e.e.* was unaffected, see Figures S28A and S30D). The obtained solid was dissolved in a mixture of CD_3CN:D_2O (1:1, *v/v*, 500 μL) in an NMR tube (~ 1.0 mM) and $NaHCO_3$ (2.1 mg, 25 equiv.) was added (Figure S28B). The sample was then heated at 90°C for 22 hours and motor **1c** racemized (0*% e.e.*, Figure S30E) with no changes observed in the ^1H NMR (Figure S28C). To adjust the pH, the above solution was acidified using 1M HCl ethyl acetate solution and all the solvent evaporated. The obtained solid was dissolved in CD_3CN and the formed NaCl salt filtered out, affording a clean ^1H NMR spectrum of racemic motor **1c** (Figure S28D).

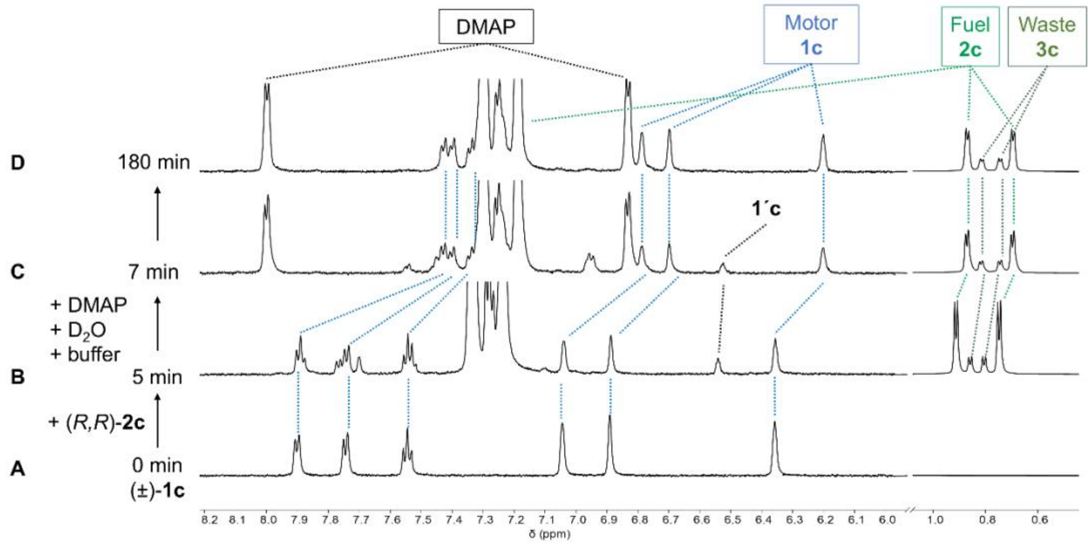

Figure S26. Singly kinetically gated stepwise anhydride formation and hydrolysis of **1c**. Partial ^{1}H NMR spectra (CD_3CN, 600 MHz, 298 K) of the stepwise operation. The region 6.1–8.2 ppm is scaled vertically 15× compared to region 0.6–1.0 ppm. (**A**) Racemic **1c** in CD_3CN ([**1c**] = 1.0 mM) (see Figure S29A). (**B**) 5 min after addition of fuel (*R,R*)-**2c** (2 equiv., [**2c**] = 2.0 mM), which reacts with **1c** to form anhydride **1′c** and urea waste. (**C**) 2 min after addition of DMAP as an achiral anhydride hydrolysis promoter (2 equiv., [DMAP] = 2.0 mM), D_2O (50% *v/v*), basic buffer ([MOPS buffer] = 100.0 mM, pH_{obs} = 7.9) to unselectively hydrolyse anhydride **1′c** back to **1c**. The basic buffer is to quench the reaction of the fuel-to-waste reaction to be able to compute the kinetic gating of the fueling step. (**D**) 180 min after the addition of DMAP, D_2O , and buffer solution showing the complete hydrolysis of anhydride **1′c**, reforming (+)-**1c** in 10% *e.e.* (see Figure S29B).

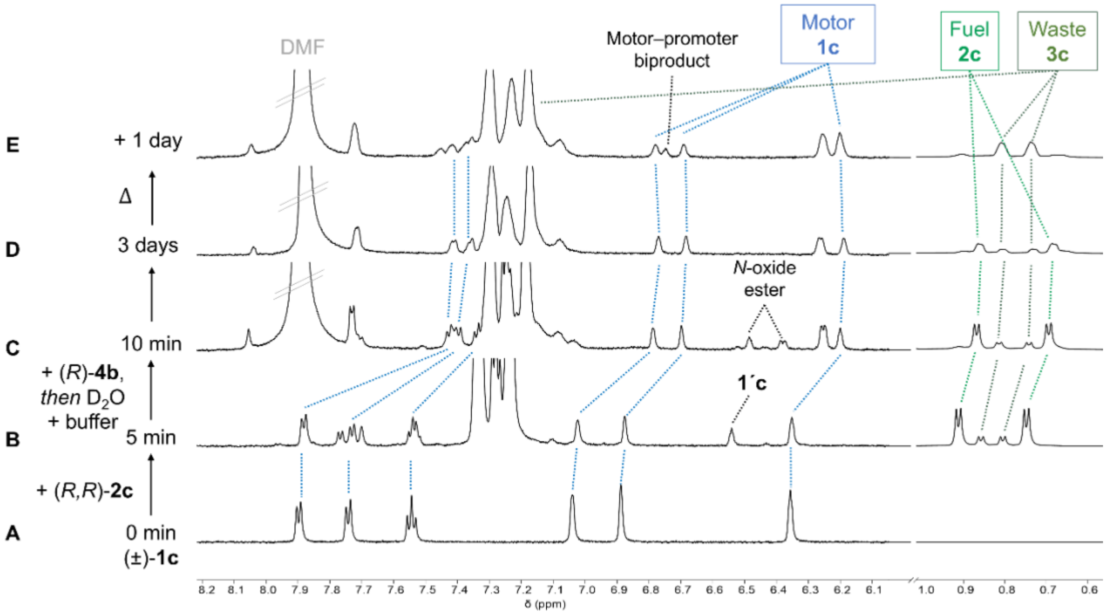

Figure S27. Doubly kinetically gated stepwise anhydride formation and hydrolysis of **1c**. Partial ^{1}H NMR spectra (CD_3CN, 600 MHz, 298 K) of the stepwise operation. The region 6.1–8.2 ppm is scaled vertically 15× compared to region 0.6–1.0 ppm. (**A**) Racemic **1c** in CD_3CN ([**1c**] = 1.0 mM) (see Figure S29A). (**B**) 5 min after addition of fuel (*R*,*R*)-**2c** (2 equiv., [**2c**] = 2.0 mM), which reacts with **1c** to form anhydride **1′c** and urea waste. (**C**) Sequential addition of chiral hydrolysis promoter (*R*)-**4b** (2 equiv., [**4b**] = 2.0 mM), D_2O (50% *v*/*v*), and basic buffer ([MOPS buffer] = 100.0 mM, $pH_{obs} = 7.9$) to selectively hydrolyse anhydride **1′c** back to **1c**. The DMF peak results from the promoter stock solution. The basic buffer is to quench the reaction of the fuel-to-waste reaction to be able to compute the kinetic gating of the fueling step. (**D**) 3 days after the addition of (*R*)-**4b**, D_2O , and buffer solution showing the complete hydrolysis of anhydride **1′c**, reforming (+)-**1c** in 54% *e.e.* (see Figure S29C). (**E**) Heating of the sample for 19 hours at 85°C to allow racemization of **1c** resulted in a motor–promoter adduct due to heating under basic conditions.

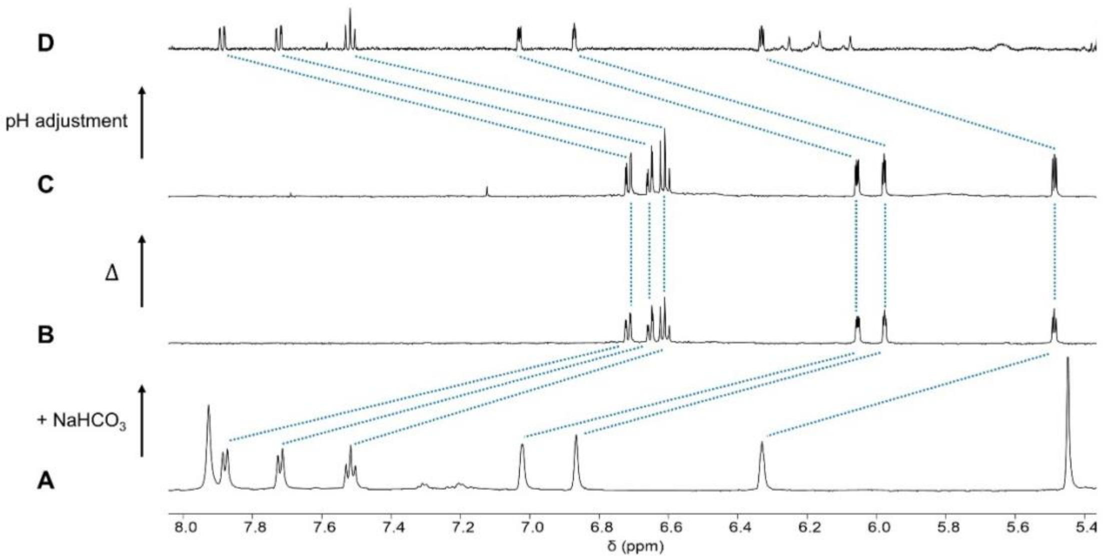

Figure S28. Partial ^{1}H NMR spectra (CD_3CN or dioxane-*d*$_8$:D_2O (1:1 *v/v*), 600 MHz, 298 K) of motor **1c** after fueling with (*R,R*)-**2c** and (*R*)-**4b**, work-up, and heating. (**A**) Acidification of the solution followed by extraction with EtOAc afforded motor **1c** without affecting the % *e.e*. (see Figure S29D). (**B**) Treatment of sample under the basic condition ([**1c**] = 1.0 mM, $NaHCO_3$ (2.1 mg), dioxane-*d*$_8$:D_2O (1:1 *v/v*), r.t. (**C**) Heating the isolated non-racemic sample of **1c** at 90°C for 22 hours allowed its racemization (see Figure S29E), demonstrating a complete 360° rotation. (**D**) Adjusting the pH afforded the same starting spectrum of **1c** in CD_3CN. The unavoidable formation of acetamide resulted from the presence of concentrated HCl in acetonitrile, however, those peaks (around 6.2 ppm) were removed from the figure to better guide the viewer.

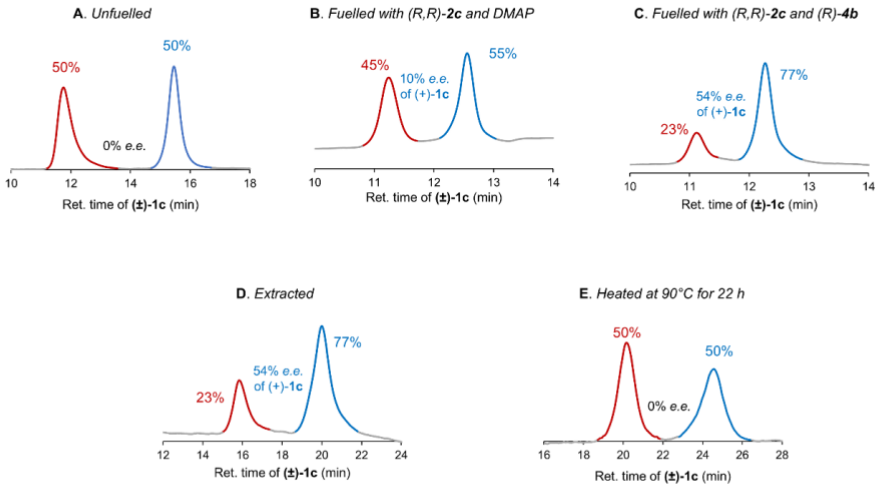

Figure S29. Chiral HPLC analysis (ChiralPak IF column, 25 °C, ^{i}PrOH:CF_3CO_2H:*n*-hexane, 1.98:0.02:98 (*v/v/v*), 2 mL min^{-1}). (**A**) Before the fueling of motor **1c**. (**B**) After the singly kinetically gated stepwise anhydride formation and hydrolysis of motor **1c** using (*R,R*)-**2c** and achiral DMAP (as shown in Figure S26D). (**C**) After the doubly kinetically gated stepwise anhydride formation and hydrolysis of motor **1c** using (*R,R*)-**2c** and (*R*)-**4b** (as shown in Figure S27D). (**D**) After work-up and extraction of motor **1c** showing the unaffected ratio of atropisomers (as shown in Figure S28A). (**E**) After heating the isolated non-racemic sample of **1c** at 90°C for 22 hours (as shown in Figure S28C) to allow its racemization, demonstrating a complete 360° rotation.

S8. Mitigating *N*-acyl urea formation

S8.1 Mechanism of degradation of motor 1

The degradation of motor **1** in the presence of carbodiimide **2c** under the re-optimized conditions with hydrolysis promoter **4b** (see Table S2, Section S5) affected the assessment of directionality. Mass spectrometry analysis showed the formation of complex *N*-acyl urea 1 and *N*-acyl urea 2 (Figure S30). Mechanistic analysis of the possible chemical transitions showed that due to the relatively long lifetime of the *N*-oxide ester that forms subsequent to the nucleophilic attack of hydrolysis promoter **4b** on cyclic anhydride **1'** (compared to the short-lived *N*-acyl DMAP-pyridinium that forms with classically used DMAP derivatives as hydrolysis promoters), the other 'free' carboxylate of the rotor has enough time to react with a molecule of fuel to form an *O*-acyl urea that, due to the unavailability of the stator carboxylate for anhydride formation, rearranges to form *N*-acyl urea 1 (Figure S31B). Subsequent hydrolysis of the *N*-oxide ester affords the stator carboxylate that undergoes the same mechanism of *N*-acyl urea rearrangement due to the unavailability of the rotor carboxylate. This mechanism also explains why the less bulky DIC had a similar effect with *N*-oxides **4b** and **4d** while the bulkier di-*tert*-butylcarbodiimide did not have that effect. For instance, the bulkier the R_1-group of the carbodiimide, the slower the reaction of the rotor carboxylate of the *N*-oxide ester adduct with a carbodiimide molecule as a result of steric clash (transition (vi), Figure S31B), leading to an even slower *N*-acyl urea rearrangement compared to the hydrolysis of the *N*-oxide ester. For carbodiimide **2c** (see Main text, Figure 3 for R_1) and DIC, the R_1 group is not bulky enough (the carbon atom adjacent to nitrogen atom of the carbodiimide is tertiary as opposed to it being quaternary in the case of di-*tert*-butylcarbodiimide), resulting in a relatively fast nucleophilic attack of the stator carboxylate on a molecule of fuel, a *N*-acyl urea rearrangement faster than the hydrolysis of the *N*-oxide ester, and a consequent degradation motor **1**.

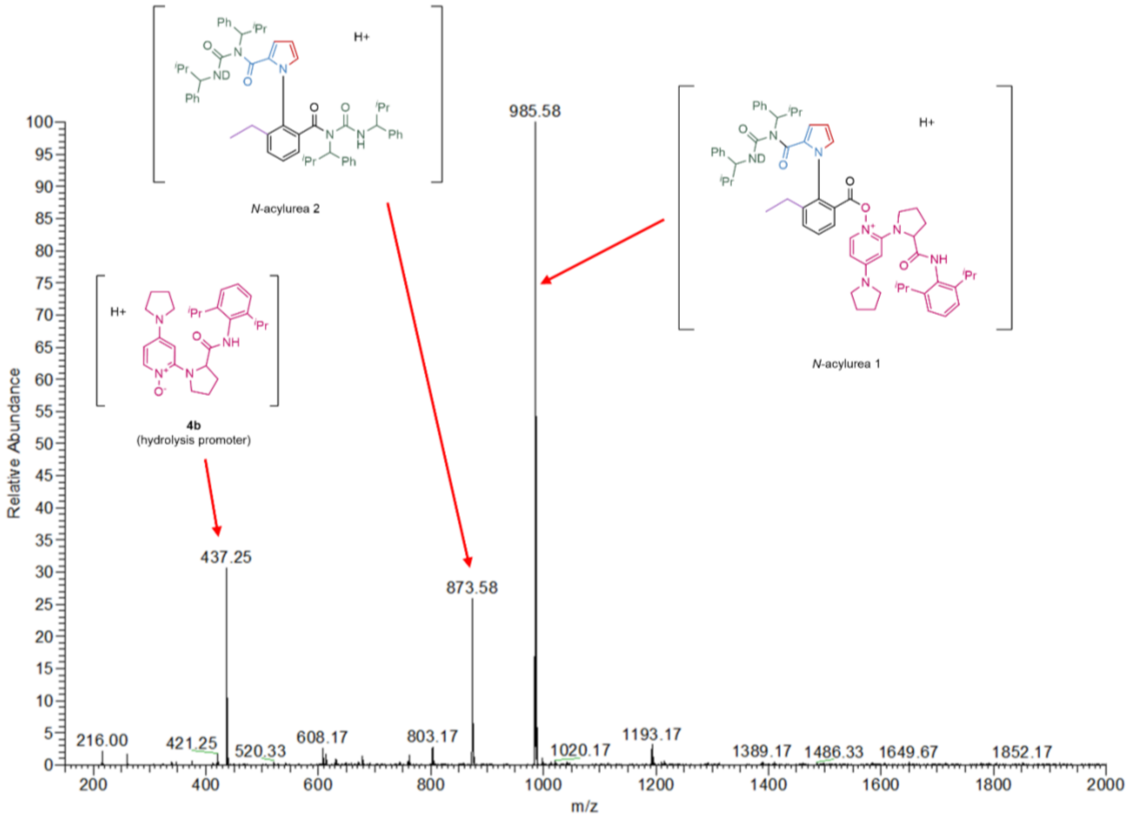

Figure S30. Low resolution ESI$^+$ mass spectrometry of a solution of [motor **1b**] = 1.0 mM, [**4b**] = 2.0 mM, [**2c**] = 8.0 mM, [MES buffer] = 100.0 mM (pH_{obs} 5.1 in D_2O), dioxane-d_8:D_2O (1:1 *v/v*) at r.t. showing the *N*-acyl urea biproducts of **1b**.

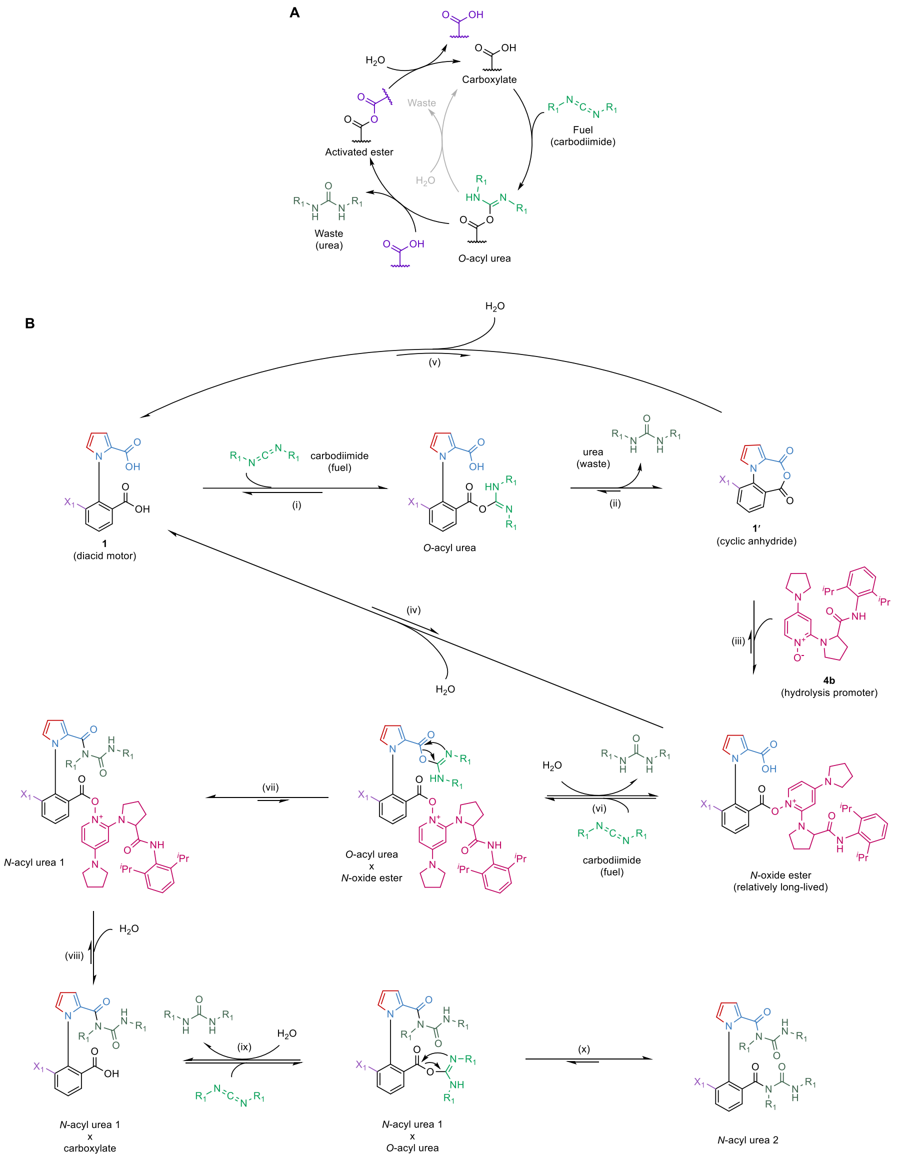

Figure S31. Scheme of chemical transitions for the fueling of motor **1**. (**A**) Catalytic cycle for the hydration of a carbodiimide using a carboxylate catalyst. (**B**) Expanded chemical scheme showing the notable chemical transformations leading (or not) to the degradation of motor **1**. All chirality is omitted for clarity. The formation of mixed anhydride oligomers and their hydrolysis back to diacid **1** may occur but is not shown for clarity. All arrows shown are equilibria for thermodynamic consistency, though equilibria shown as non-equivalent are very rare in the system under the operation conditions. For R_1, see Main text, Figure 3, entry 3, **2c**. (i) Reaction of a carbodiimide molecule on di-acid motor **1** generates an *O*-acyl urea. (ii) Nucleophilic attack on the *O*-acyl urea to form cyclic anhydride **1'**. (iii) Ring opening of **1'** with hydrolysis promoter **4b** to form a relatively long-lived *N*-oxide ester. (iv) Hydrolysis of the *N*-oxide ester affords di-acid motor **1**. (v) Unpromoted hydrolysis of cyclic anhydride **1'** back to di-acid motor **1**. (vi) Reaction of the rotor carboxylate with a carbodiimide molecule yields another *O*-acyl urea species. (vii) Rearrangement of the *O*-acyl urea to the unreactive *N*-acyl urea 1 due to the relatively slow hydrolysis of the *N*-oxide ester of the stator. (viii) Eventual hydrolysis of the *N*-oxide ester affords the stator carboxylate. (ix) Reaction of the stator carboxylate with a carbodiimide molecule yields another *O*-acyl urea species. (x) Rearrangement of the *O*-acyl urea to another unreactive *N*-acyl urea 2 due to the unavailability of the other carboxylate for nucleophilic attack.

S8.2 Using HOBt to minimize *N*-acyl urea formation

There are a number of effective ways to minimize the issue of *N*-acyl urea formation:

a) Increase the steric bulk of the fuel

b) Maintain the fuel concentration at ≤ 1 equiv. at all times (see Section S6.3 for details)

c) Add HOBt to the fueling mixture

Having a much bulkier chiral carbodiimide (like di-*tert*-butylcarbodiimide) did not seem ideal as it would drastically slow down the fueling reaction, and consequently slow down the speed of rotation of the motor. On the other hand, adding HOBt to the reaction mixture would protect the rotor carboxylate for long enough to allow the *N*-oxide ester to hydrolyze seemed to be a viable solution (Figure S32). The addition of HOBt would further prove the mechanism of *N*-acyl urea formation shown in Figures S30 and S31.

Preparation of sample

[Racemic (±)-**1b**] = 1.0 mM, [(*S*)-**4b**] = 2.0 mM, [(*S,S*)-**2c**] = 10.0 mM, [HOBt] = 10.0 mM, [MES buffer] = 100 mM (pH_{obs} 5.10 in D_2O) dioxane-d_8:D_2O (1:1 *v/v*) at r.t.

In an NMR tube, racemic (±)-**1b** (5.0 μL of a 0.1 M stock solution in DMF) was diluted into 250 μL of dioxane-d_8. To the solution was added 180 μL of D_2O, 100 mM of MES buffer (50 μL of a 1.0 M stock solution in D_2O, pH_{obs} 5.10), hydrolysis promoter (*S*)-**4b** (4.0 μL of a 0.25 M stock solution in DMF), and HOBt (25 μL of a 0.2 M stock solution in dioxane-d_8). Fuel (*S,S*)-**2c** (25 μL of a 0.2 M stock solution in dioxane-d_8, 10.0 mM) was added and the reaction was monitored over time by ^{1}H NMR spectroscopy (Figure S33). At the end of the experiment, a 20 μL aliquot was taken and examined by chiral HPLC (Figure S34).

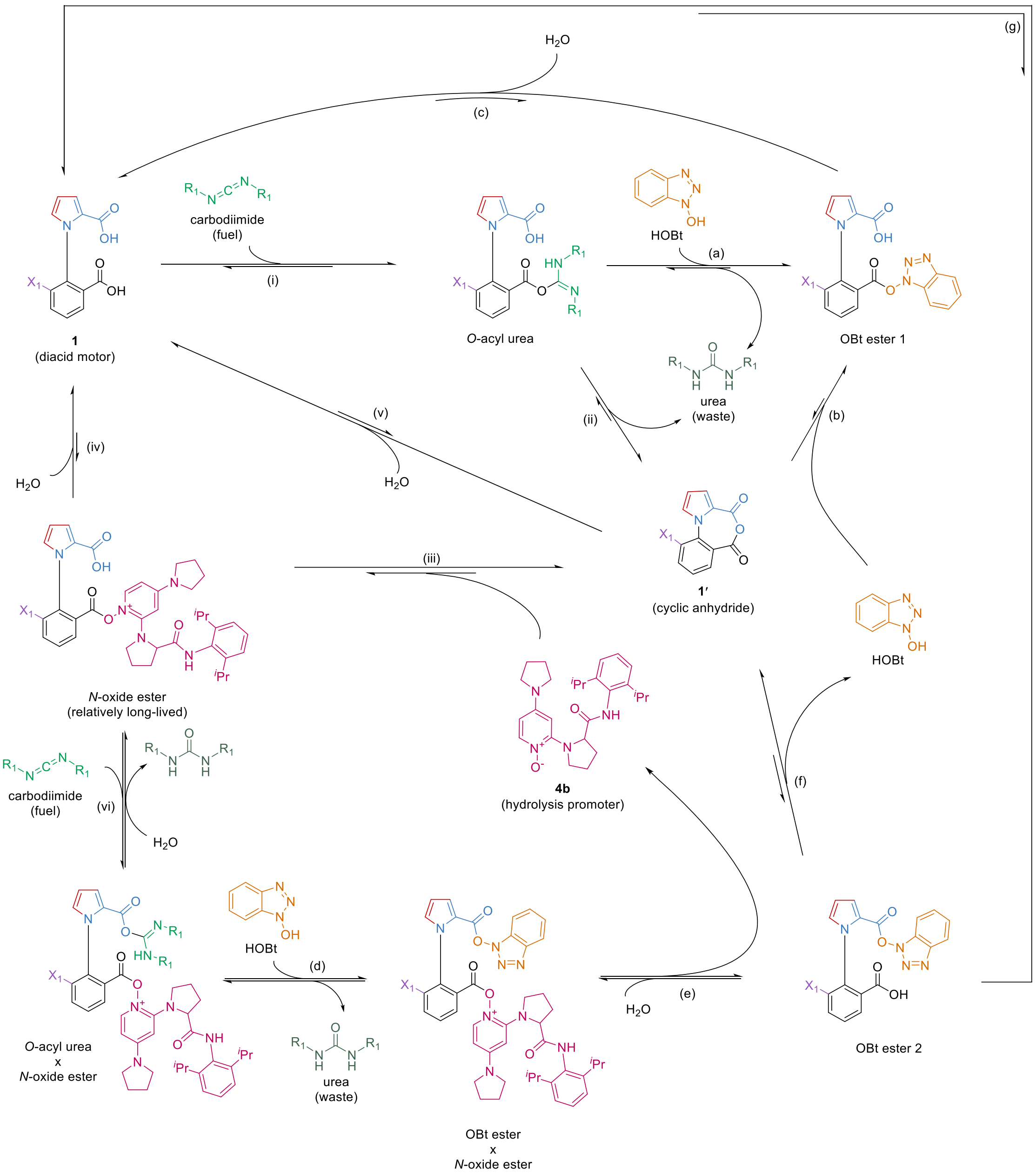

Figure S32. Scheme of chemical transitions for the fueling of motor **1** in the presence of HOBt. All chirality is omitted for clarity. The formation of mixed anhydride oligomers and their hydrolysis back to di-acid **1** may occur but is not shown for clarity. All arrows shown are equilibria for thermodynamic consistency, though equilibria shown as non-equivalent are very rare in the system under the operation conditions. For R_1, see Main text, Figure 3, entry 3, **2c**. Chemical transitions (i)-(vi) are identical to those in Figure S31. (a) Reaction of *O*-acyl urea with HOBt to form activated OBt ester 1. (b) Nucleophilic attack of HOBt on cyclic anhydride **1'** to form activated OBt ester 1. (c) Hydrolysis of OBt ester 1 into di-acid motor **1**. OBt ester 1 can also undergo promoter-mediated hydrolysis (analogous to transition (iii)) and yield the relatively long-lived *N*-oxide ester (this transition is not shown for clarity). (d) Reaction of HOBt with the rotor *O*-acyl urea, previously prone to *N*-acyl urea rearrangement, to afford a now protected carboxylate in the form of an activated OBt ester. (e) Hydrolysis of the *N*-oxide ester affords OBt ester 2. (f) Nucleophilic attack of the carboxylate on activated OBt ester 2 to form cyclic anhydride **1'**. (g) Hydrolysis of OBt ester 2 to afford di-acid motor **1**.

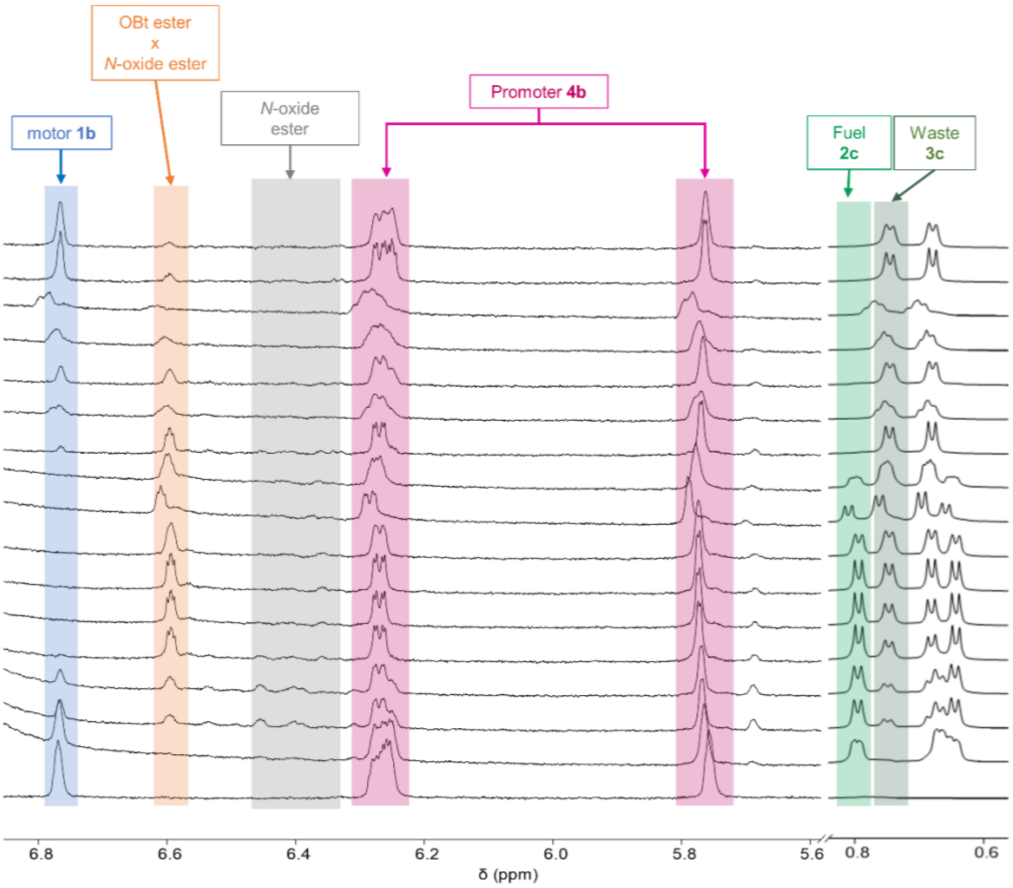

Figure S33. Partial ^{1}H NMR spectra of the fueling reaction of motor **1b** using HOBt and fuel **2c**. The spectra show the formation of *N*-oxide ester (grey) that quickly reacts with HOBt giving the protected species (orange). After complete fuel consumption, motor **1b** is recovered entirely with no *N*-acyl urea formation. ([Racemic (±)-**1b**] = 1.0 mM, [(*S*)-**4b**] = 2.0 mM, [(*S,S*)-**2c**] = 10.0 mM, [HOBt] = 10.0 mM, [MES buffer] = 100 mM (pH_{obs} 5.10 in D_2O) dioxane-d_8/D_2O (1:1 *v/v*) at r.t.).

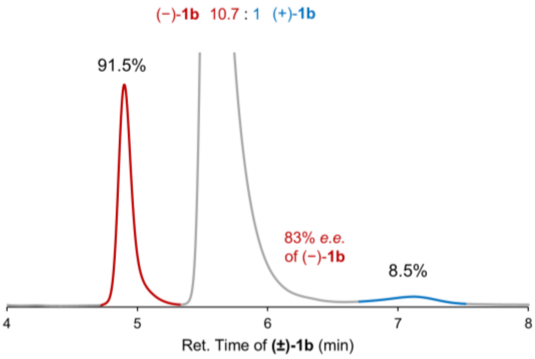

Figure S34. Chiral HPLC trace analysis (ChiralPak IF column, 25 °C, CH_2Cl_2:*i*-PrOH:CF_3CO_2H:*n*-hexane, 66.5:3.4:0.1:30 (*v/v/v/v*), 2 mL min^{-1}) of **(±)-1b** subsequent to fueling with **2c** in the presence of (*S*)-**4b** and HOBt under re-optimized conditions. ([Racemic **(±)-1b**] = 1.0 mM, [(*S*)-**4b**] = 2.0 mM, [(*S,S*)-**2c**] = 10.0 mM, [HOBt] = 10.0 mM, [MES buffer] = 100 mM (pH_{obs} 5.10 in D_2O) dioxane-d_8/D_2O (1:1 *v/v*) at r.t.)

S9. Probabilities of the different chemomechanical cycles

The relative proportions of the different fuel consuming cycles can be estimated based on the chemical gating. Using the assumptions that:

a) mechanical exchange between anhydride atropisomers (−)-**1'** and (+)-**1'** is fast relative to the chemical steps,
b) mechanical gating is perfect,
c) there are no power strokes,

all of which are valid in this case, the cycles are given by the product of the relative probabilities of competing pathways, normalized by the sum of all equivalent pathways. The probabilities of the four fuel consuming cycles (per catalyzed fuel-to-waste transition) are therefore found to be

$$p(\boldsymbol{F}) \approx \frac{g_{\text{form}}}{g_{\text{form}}+1} \times \frac{g_{\text{hyd}}}{g_{\text{hyd}}+1} = \frac{1.25}{1.25+1} \times \frac{18.7}{18.7+1} = 0.53 \qquad \textit{(Eq. 5a)}$$

$$p(\boldsymbol{B}) \approx \frac{1}{g_{\text{form}}+1} \times \frac{1}{g_{\text{hyd}}+1} = \frac{1}{1.25+1} \times \frac{1}{18.7+1} = 0.02 \qquad \textit{(Eq. 5b)}$$

$$p(\text{Futile}_1) \approx \frac{1}{g_{\text{form}}+1} \times \frac{g_{\text{hyd}}}{g_{\text{hyd}}+1} = \frac{1}{1.25+1} \times \frac{18.7}{18.7+1} = 0.42 \qquad \textit{(Eq. 5c)}$$

$$p(\text{Futile}_2) \approx \frac{g_{\text{form}}}{g_{\text{form}}+1} \times \frac{1}{g_{\text{hyd}}+1} = \frac{1.25}{1.25+1} \times \frac{1}{18.7+1} = 0.03 \qquad \textit{(Eq. 5d)}$$

Where $p(\boldsymbol{F})$ and $p(\boldsymbol{B})$ are the (unitless) probabilities of the forward and backward fuel consuming cycles, while g_{form} and g_{hyd} are the chemical gatings for the formation and hydrolysis reactions respectively. These cycles account for all fuel-consuming pathways in the reaction network so the current around each can be found by combining these probabilities with the rate of fuel use by the motor. Other cycles, that do not use fuel (slip cycles) are not accounted for by this method, which considers only the weightings of fuel-to-waste pathways.

These simple equations can be adapted to account for non-perfect mechanical gating[S8] or to include a power stroke.[S9] Non-fast mechanical exchange or more complex reaction networks often require a more complete level of theory.[S10, S11]

S10. Estimating the rotation rate of the motor at steady-state

Rotation rate is controlled by the rate of fuel use by the motor and the directionality associated with that fuel use. As a result, rotation rate can be simply estimated at steady state:

$$\text{rotation rate } (\text{s}^{-1}) = \left(p(\boldsymbol{F}) - p(\boldsymbol{B})\right) \times \frac{k_{\text{cat}}}{[\mathbf{1}]} \qquad \textit{(Eq. 6a)}$$

$$\approx \left(\left(\frac{g_{\text{form}}}{g_{\text{form}}+1} \times \frac{g_{\text{hyd}}}{g_{\text{hyd}}+1}\right) - \left(\frac{1}{g_{\text{form}}+1} \times \frac{1}{g_{\text{hyd}}+1}\right)\right) \times \frac{k_{\text{supp}}}{[\mathbf{1}]} \qquad \textit{(Eq. 6b)}$$

Where $p(\boldsymbol{F})$ and $p(\boldsymbol{B})$ are the (unitless) probabilities of the forward and backward fuel consuming cycles, while g_{form} and g_{hyd} are the chemical gatings for the formation and hydrolysis reactions respectively. The pseudo-0th order rate constant, k_{cat} (units of M s^{-1}), describes the conditions-specific machine-catalyzed fuel consumption at a given steady state. Under the experimental conditions with slow addition (and hence low concentration) of fuel, the rate of the uncatalyzed process is negligible, so $k_{cat} \approx k_{supp}$, the 0th order rate constant for fuel addition (also units of M s^{-1}). The rate constants are both normalized by the concentration of motor **1** (i.e. given as equivalents s^{-1}) to give the average number of rotations per motor. The whole equation can be rationalized as the directional bias resulting from a single fuel consumption event multiplied by the frequency of fuel consumption events.

Numerical evaluation with experimentally obtained numbers gives:

$$\approx \left(\left(\frac{1.25}{1.25+1} \times \frac{18.7}{18.7+1}\right) - \left(\frac{1}{1.25+1} \times \frac{1}{18.7+1}\right)\right) \times \frac{1.39 \times 10^{-8}\ \text{M s}^{-1}}{1 \times 10^{-3}\ \text{M}}$$

$$\approx (0.53 - 0.02) \times 1.39 \times 10^{-5}\ \text{s}^{-1} = 7.01 \times 10^{-6}\ \text{s}^{-1} \qquad \textit{(Eq. 7)}$$

resulting in a rotation taking an average of approximately 39 hours 40 minutes.

Comparatively, with the chemical gating obtained using the original reagents[S1] the evaluation of rotation rate with the same addition rate described in the main text produces a rotation rate of approximately 99 hours 20 minutes.

$$\approx \left(\left(\frac{1.1}{1.1+1} \times \frac{2.1}{2.1+1}\right) - \left(\frac{1}{1.1+1} \times \frac{1}{2.1+1}\right)\right) \times \frac{1.39 \times 10^{-8}\ \mathrm{M\ s^{-1}}}{1 \times 10^{-3}\ \mathrm{M}}$$

$$\approx (0.35 - 0.015) \times 1.39 \times 10^{-5}\ \mathrm{s^{-1}} = 2.79 \times 10^{-6}\ \mathrm{s^{-1}} \qquad \textit{(Eq. 8)}$$

S11. NMR Spectra of New Compounds

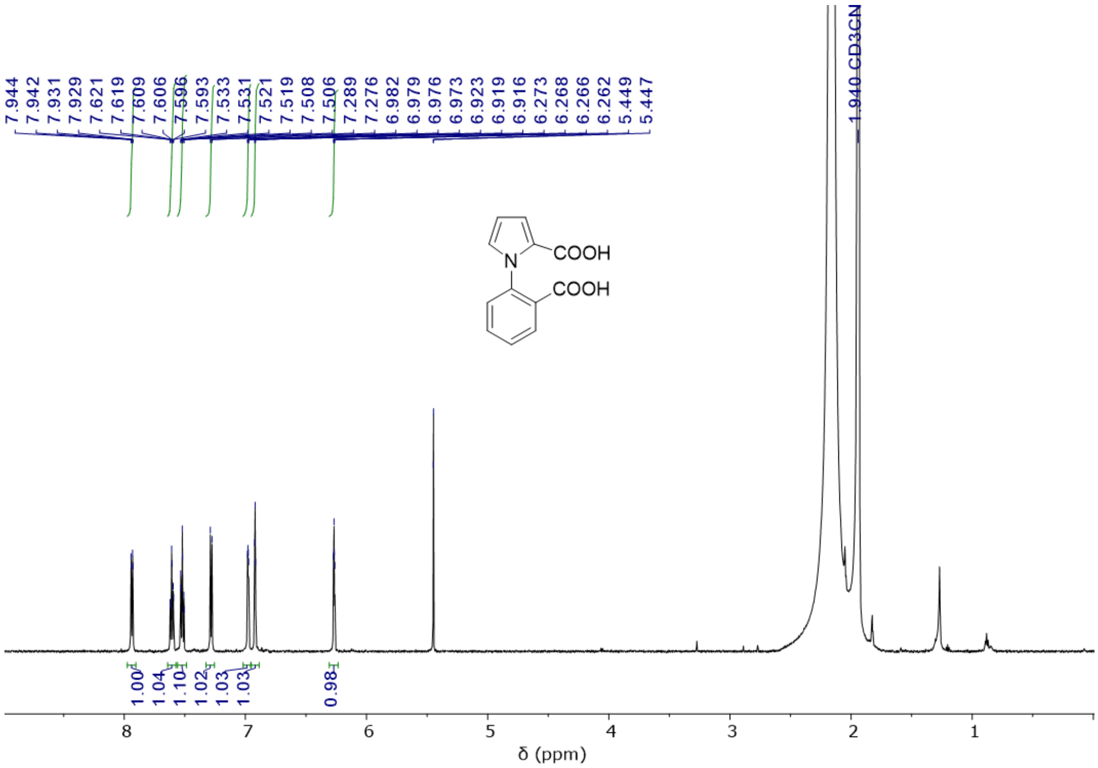

Spectrum S1. ^{1}H NMR (CD_3CN, 600 MHz, 298 K) spectrum of **motor 1a**.

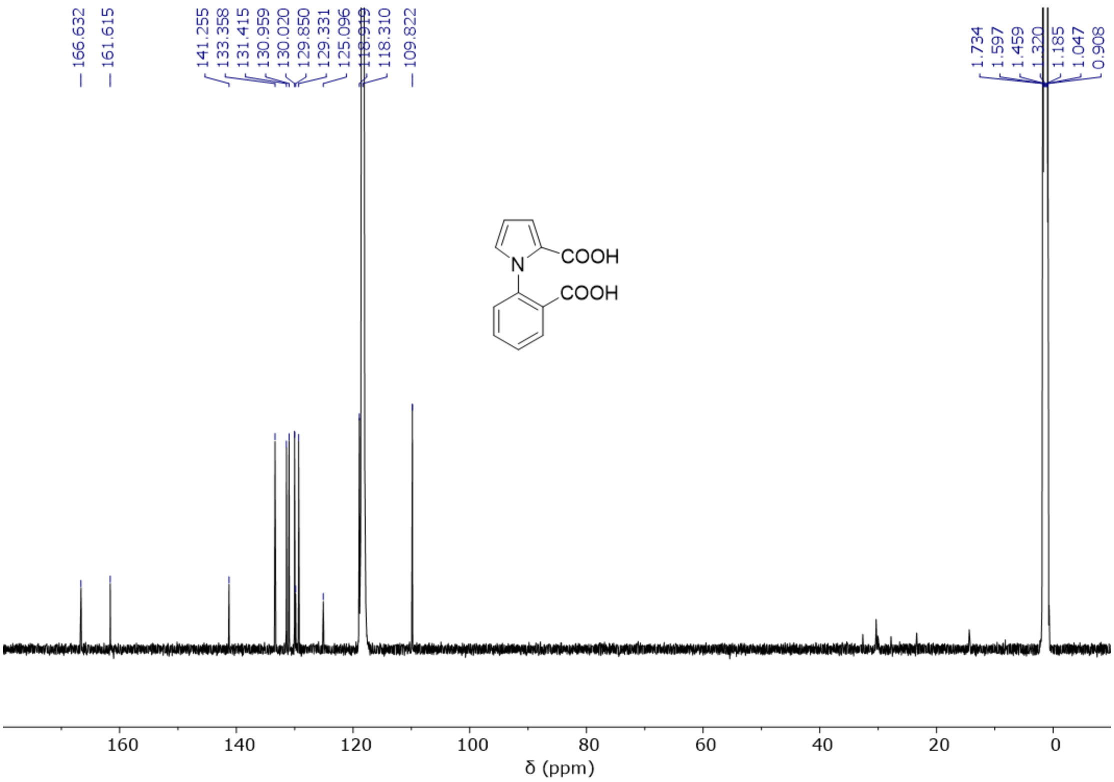

Spectrum S2. ^{13}C NMR (CD_3CN, 151 MHz, 298 K) spectrum of **motor 1a**.

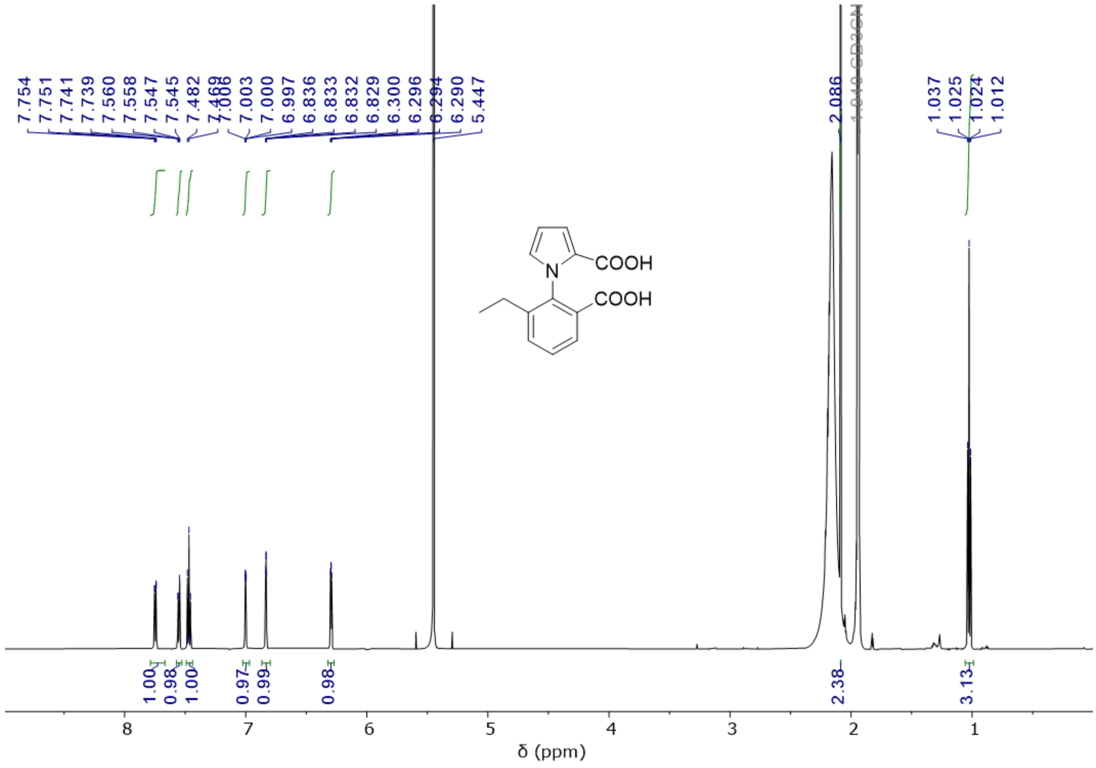

Spectrum S3. ^{1}H NMR (CD_3CN, 600 MHz, 298 K) spectrum of **motor 1b**.

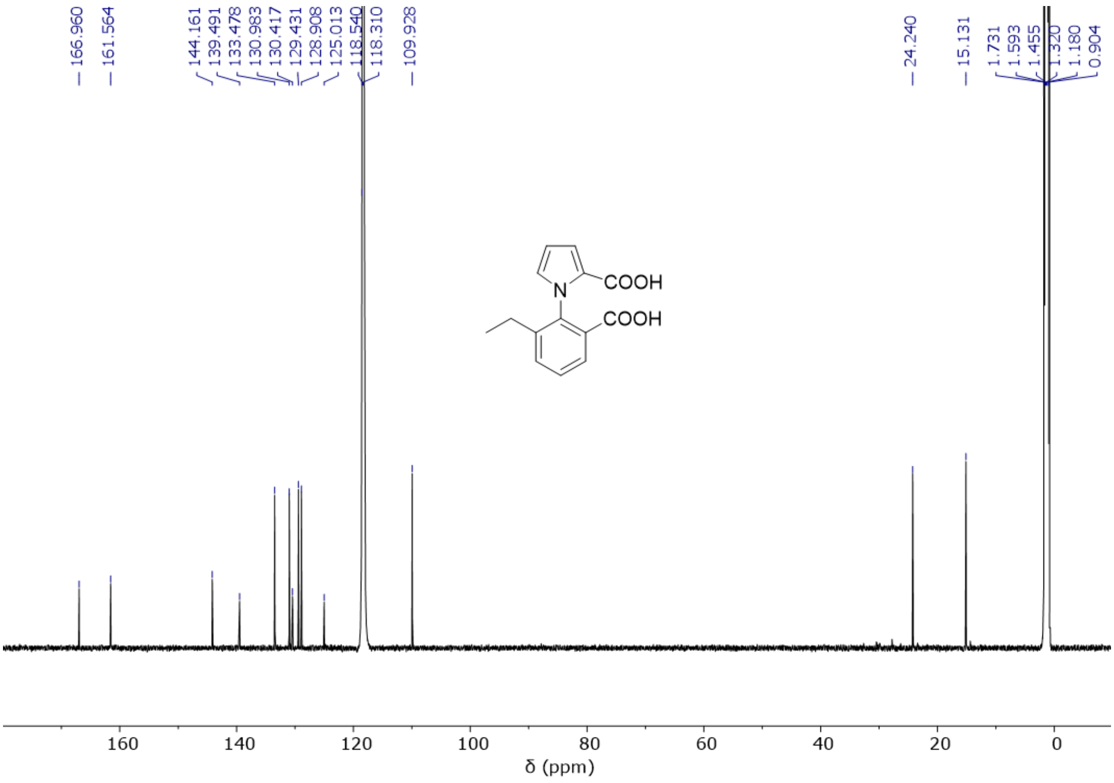

Spectrum S4. ^{13}C NMR (CD_3CN, 151 MHz, 298 K) spectrum of **motor 1b**.

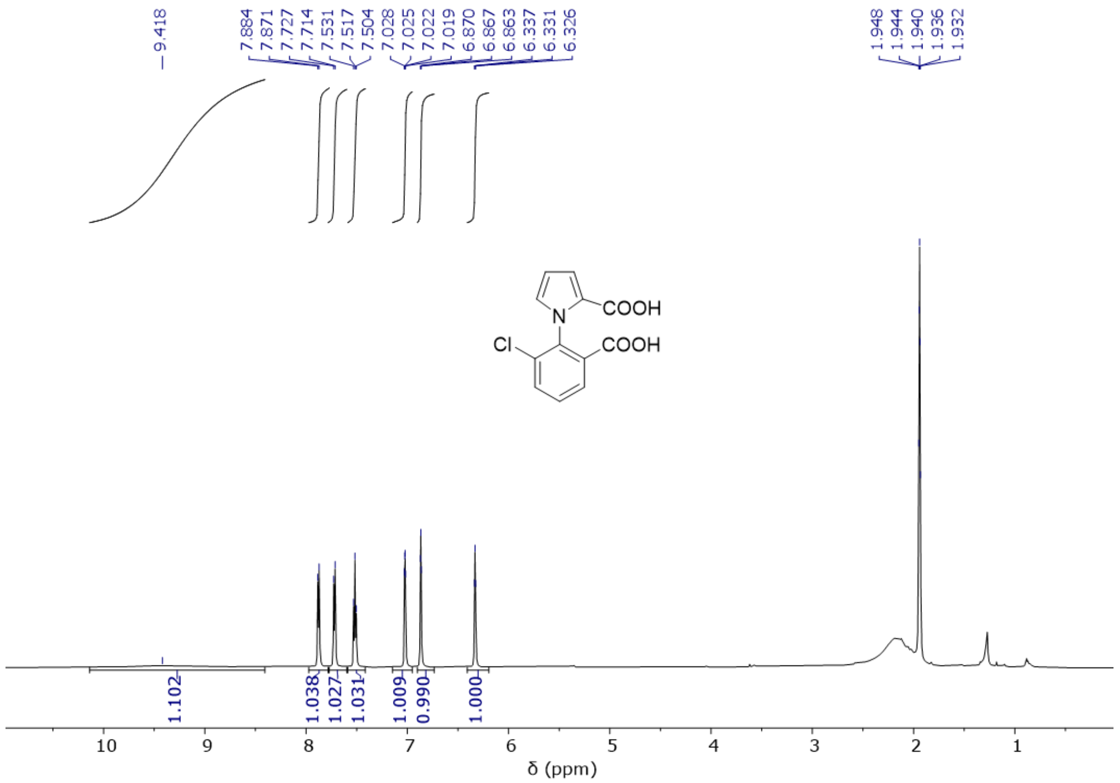

Spectrum S5. ^{1}H NMR (CD_3CN, 600 MHz, 298 K) spectrum of **motor 1b**.

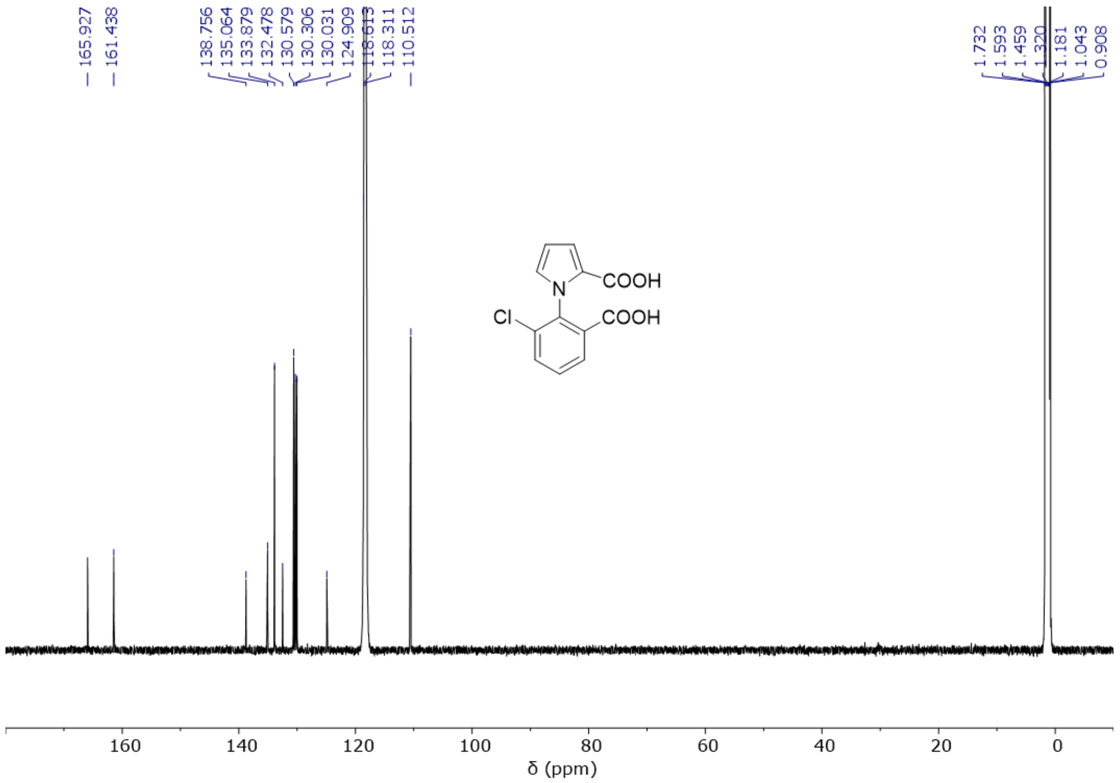

Spectrum S6. ^{13}C NMR (CD_3CN, 151 MHz, 298 K) spectrum of **motor 1c**.

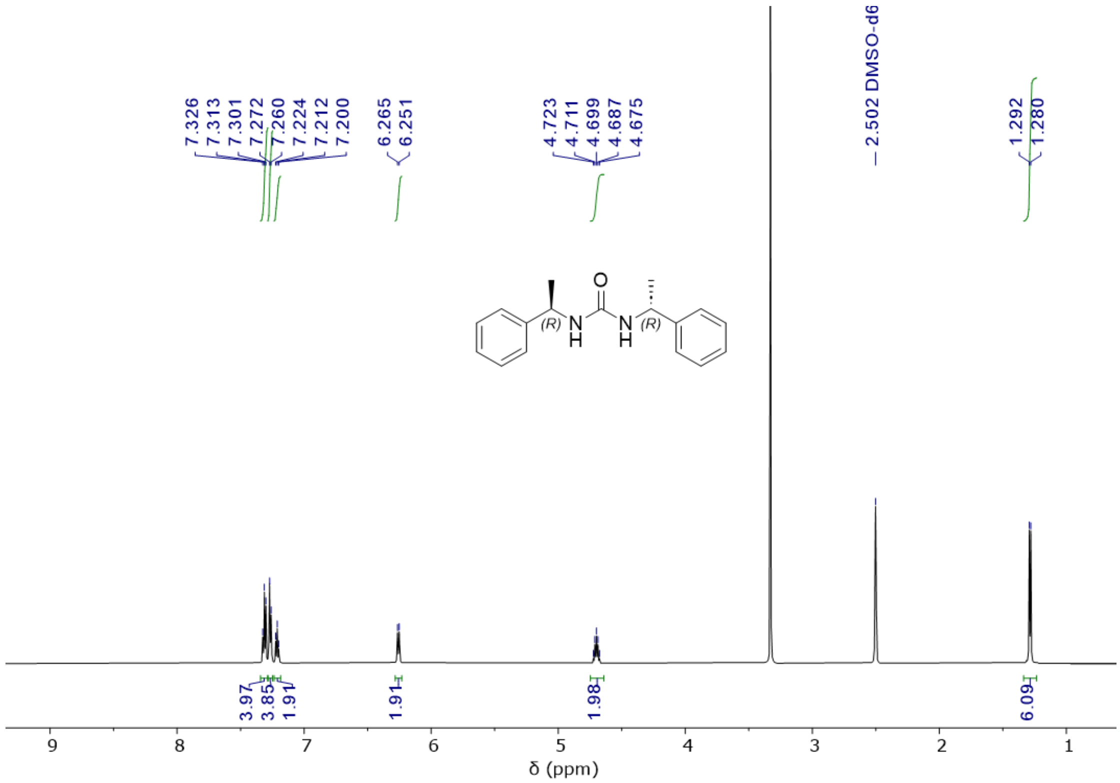

Spectrum S7. ^{1}H NMR (DMSO-d_6, 600 MHz, 298 K) spectrum of **3a**.

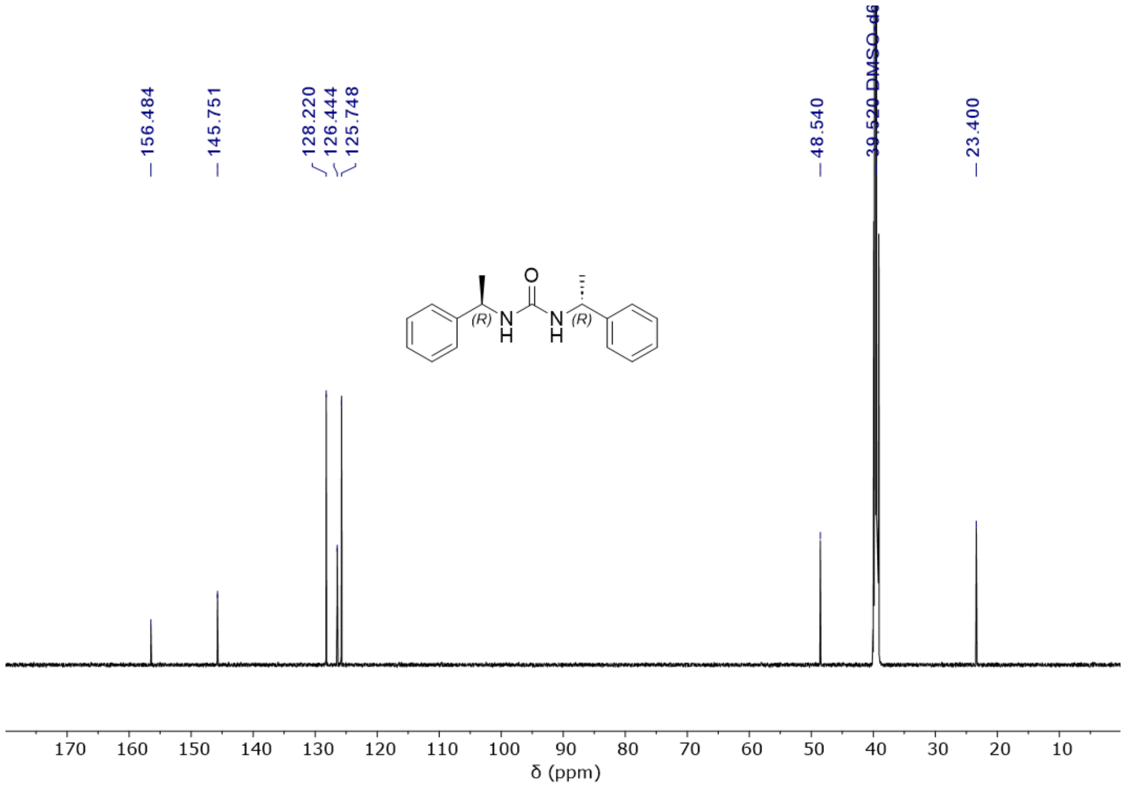

Spectrum S8. ^{13}C NMR (DMSO-d_6, 151 MHz, 298 K) spectrum of **3a**.

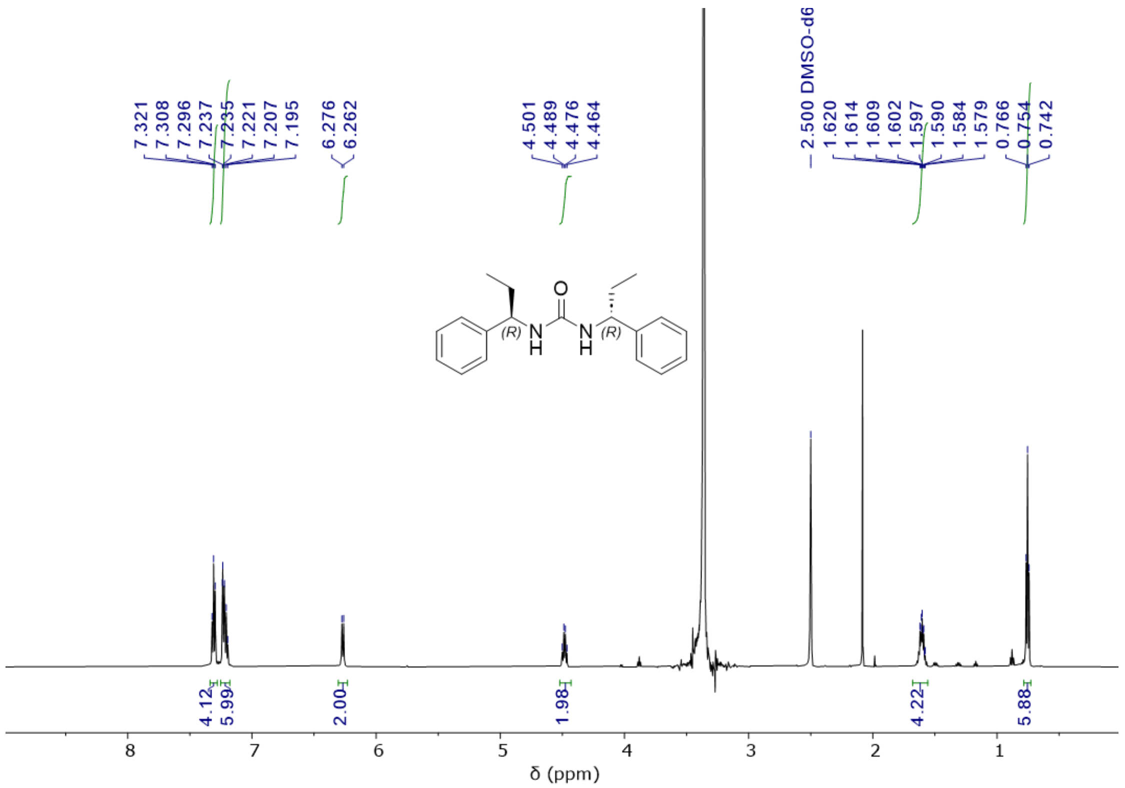

Spectrum S9. ^{1}H NMR (DMSO-d_6, 600 MHz, 298 K) spectrum of **3b**.

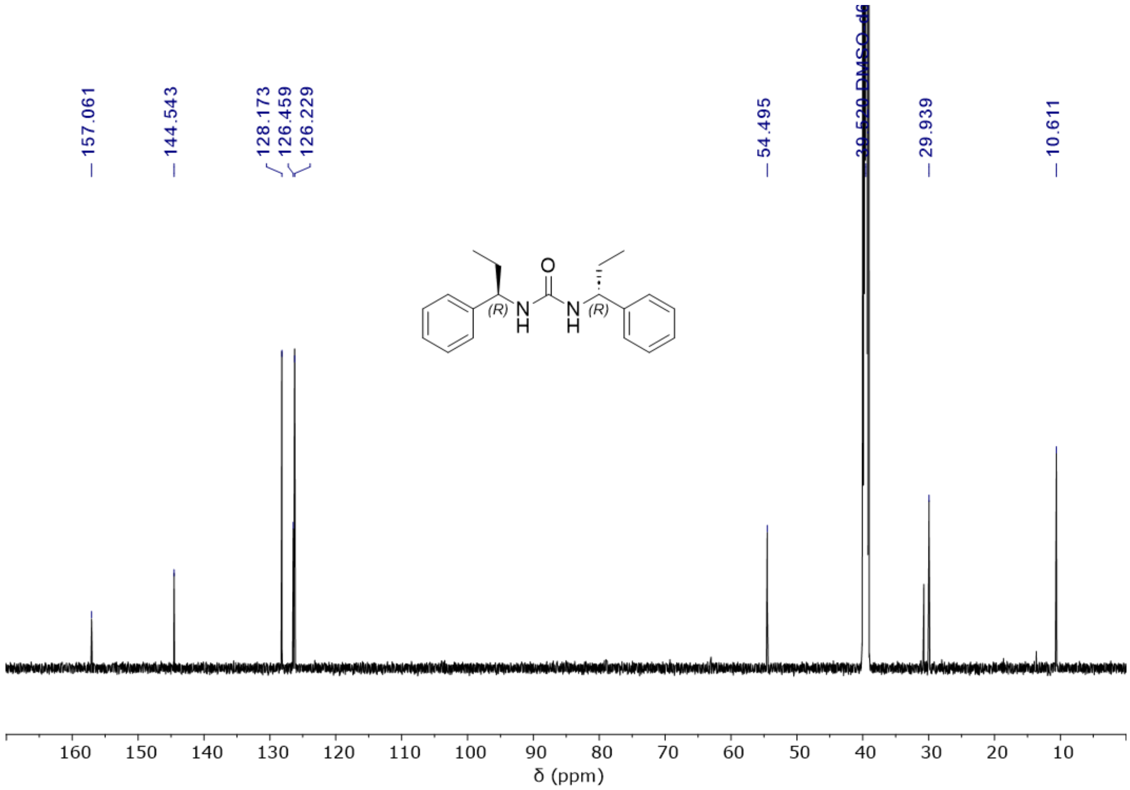

Spectrum S10. ^{13}C NMR (DMSO-d_6, 151 MHz, 298 K) spectrum of **3b**.

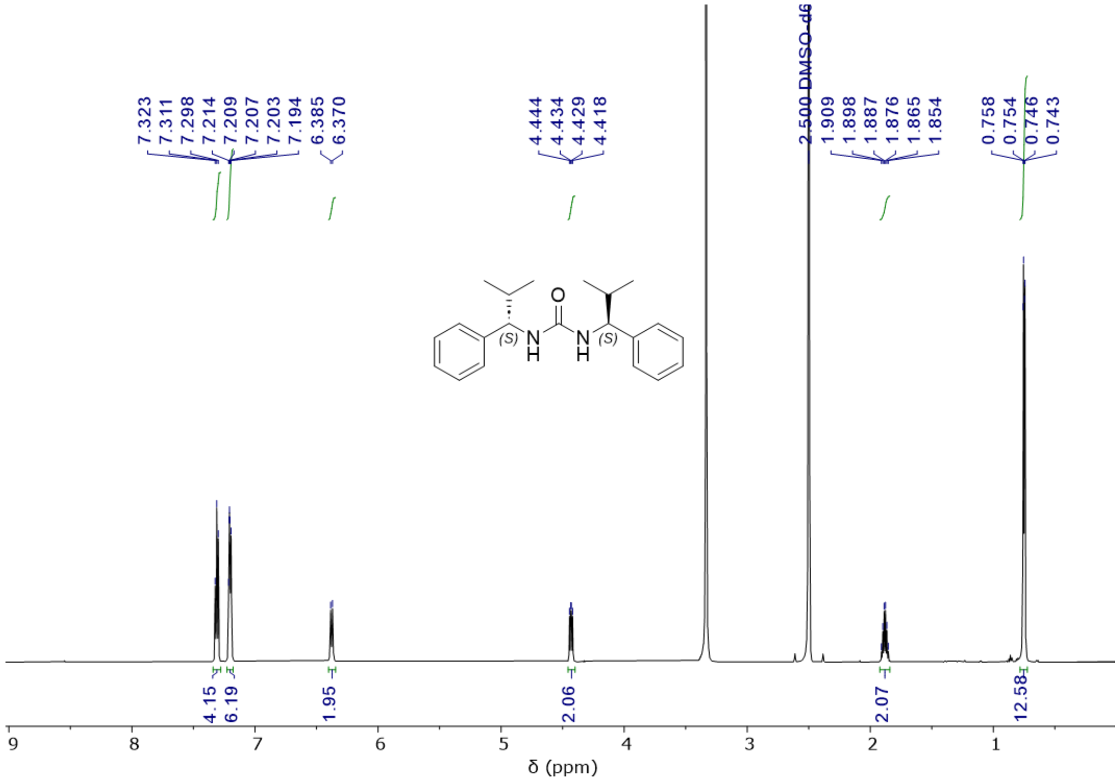

Spectrum S11. ^{1}H NMR (DMSO-d_6, 600 MHz, 298 K) spectrum of **3c**.

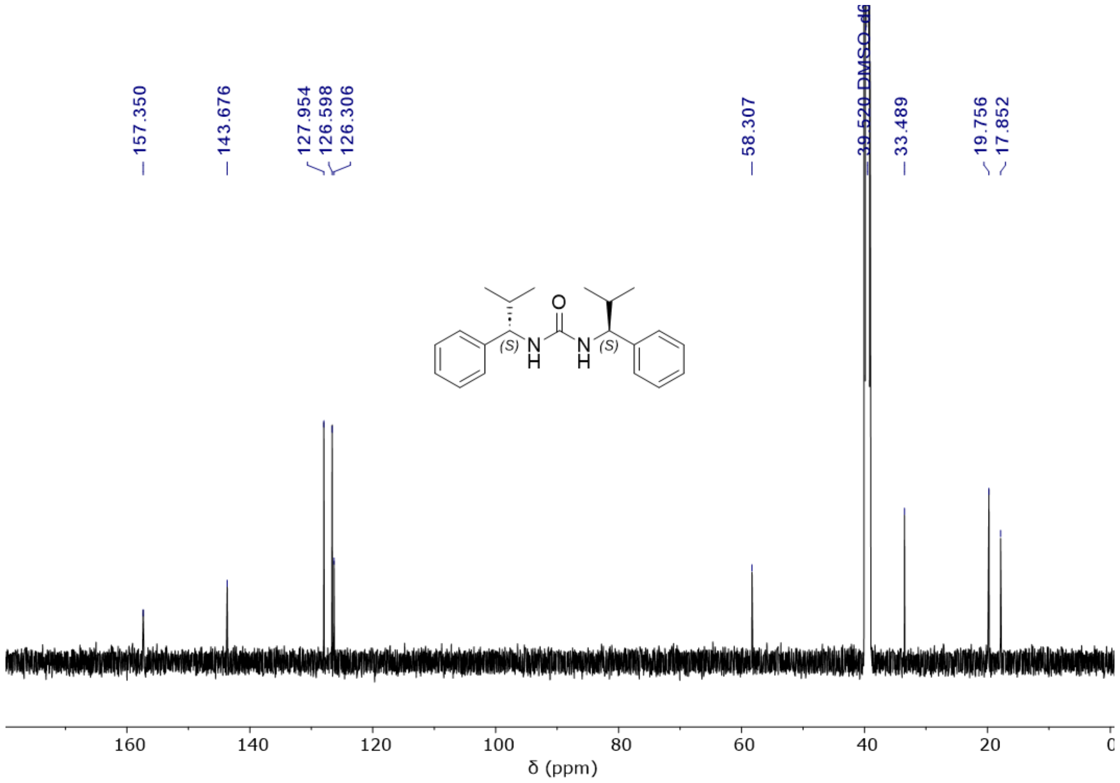

Spectrum S12. ^{13}C NMR (DMSO-d_6, 151 MHz, 298 K) spectrum of **3c**.

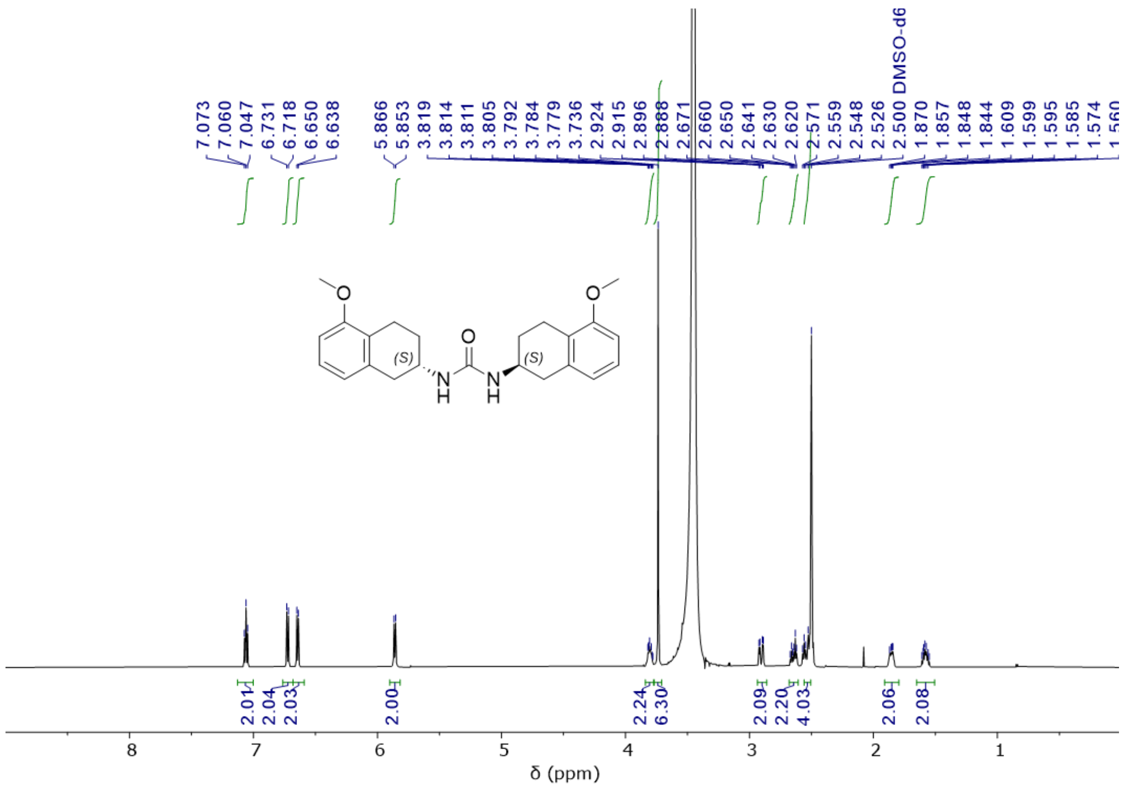

Spectrum S13. ^{1}H NMR (DMSO-d_6, 600 MHz, 298 K) spectrum of **3d**.

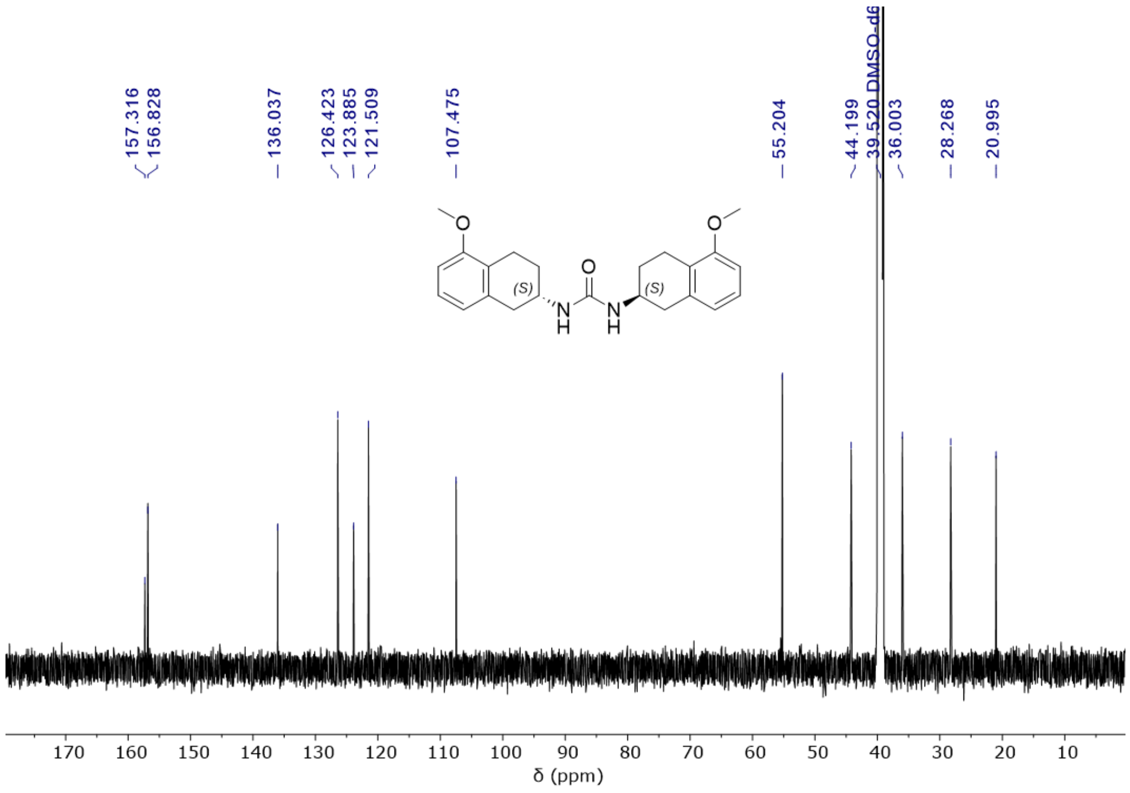

Spectrum S14. ^{13}C NMR (DMSO-d_6, 151 MHz, 298 K) spectrum of **3d**.

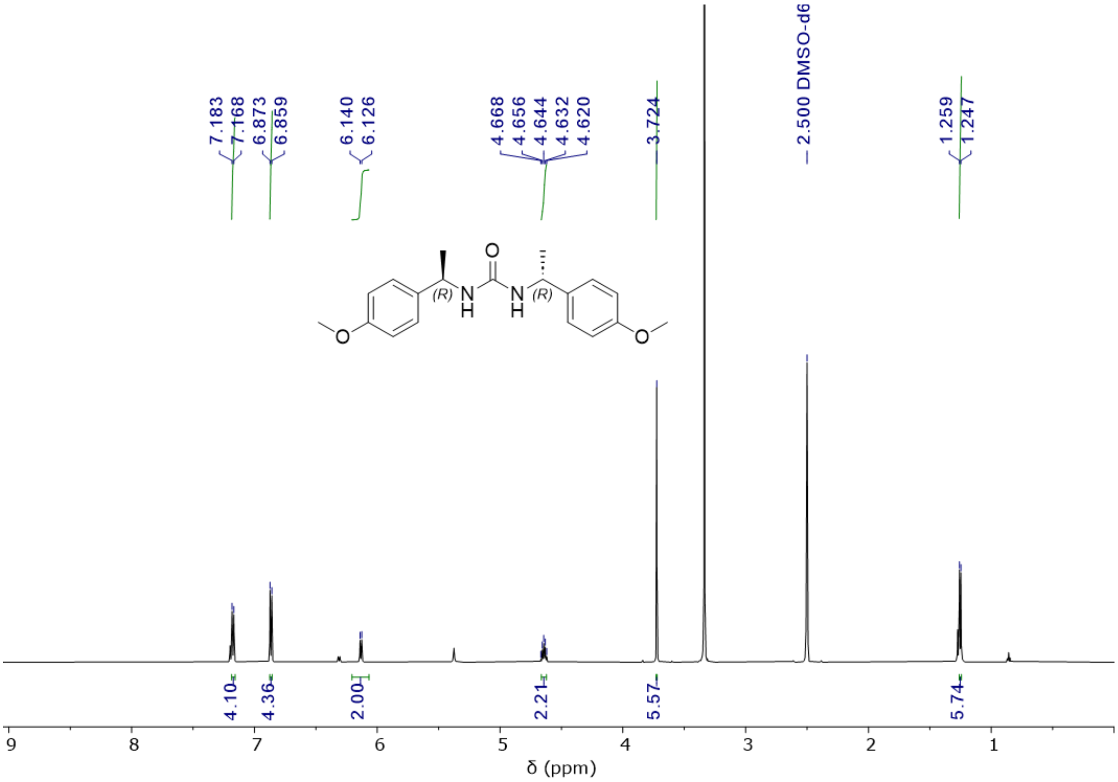

Spectrum S15. ^{1}H NMR (DMSO-d_6, 600 MHz, 298 K) spectrum of **3e**.

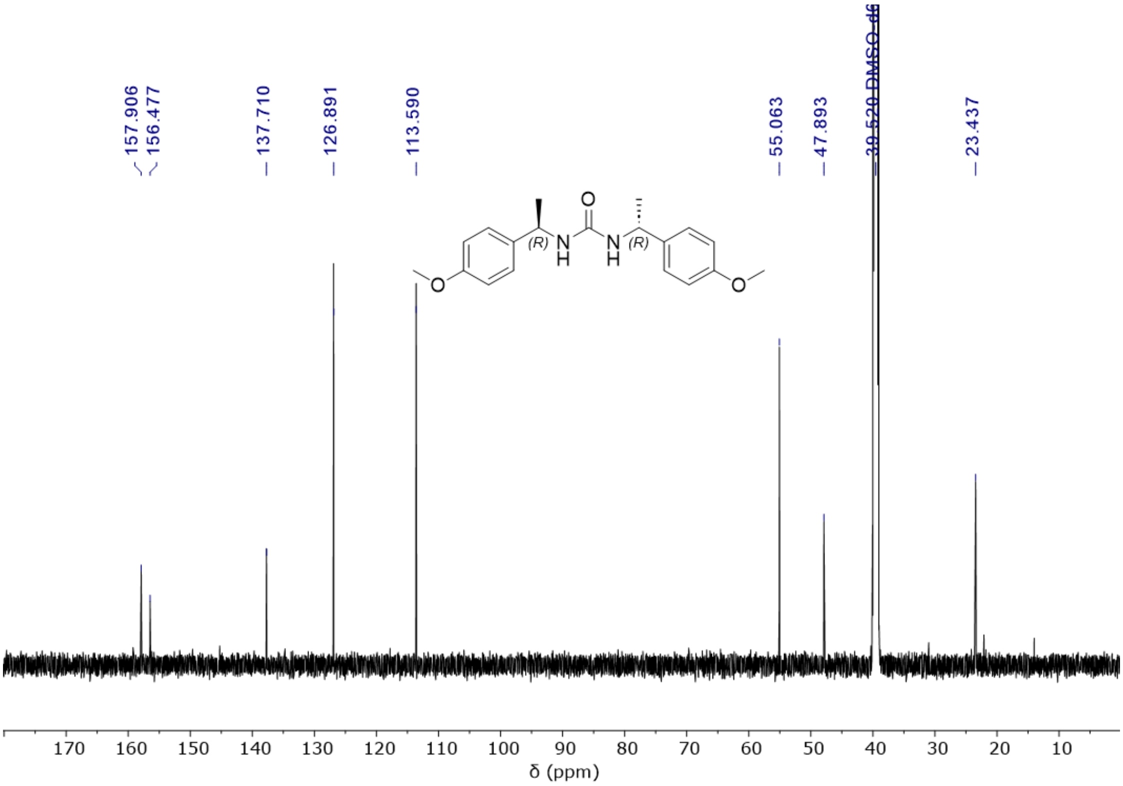

Spectrum S16. ^{13}C NMR (DMSO-d_6, 151 MHz, 298 K) spectrum of **3e**.

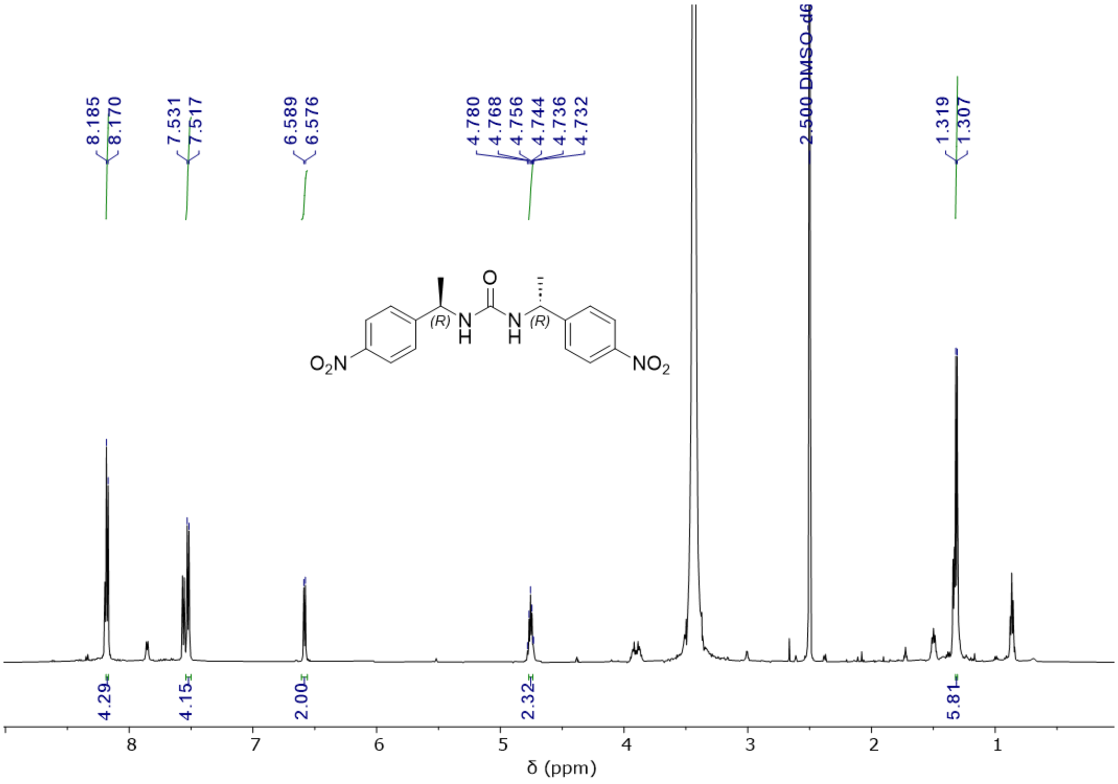

Spectrum S17. ^{1}H NMR (DMSO-d_6, 600 MHz, 298 K) spectrum of **3f**.

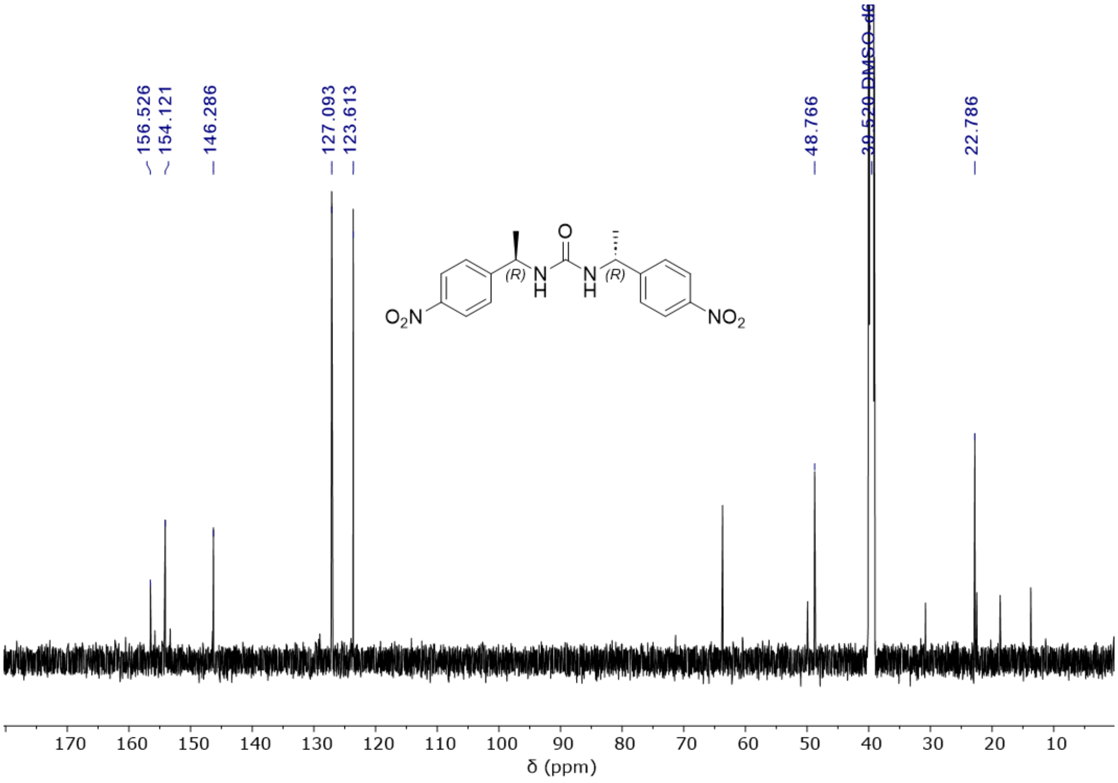

Spectrum S18. ^{13}C NMR (DMSO-d_6, 151 MHz, 298 K) spectrum of **3f**.

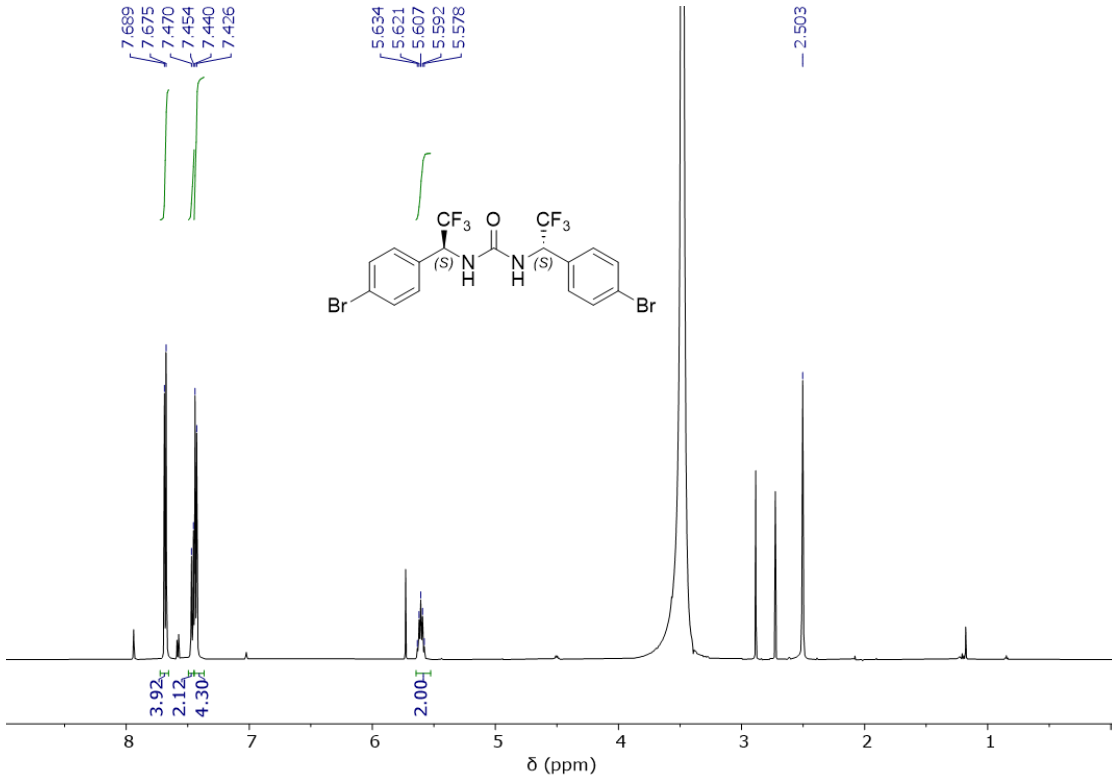

Spectrum S19. ^{1}H NMR (DMSO-d_6, 600 MHz, 298 K) spectrum of **3g**.

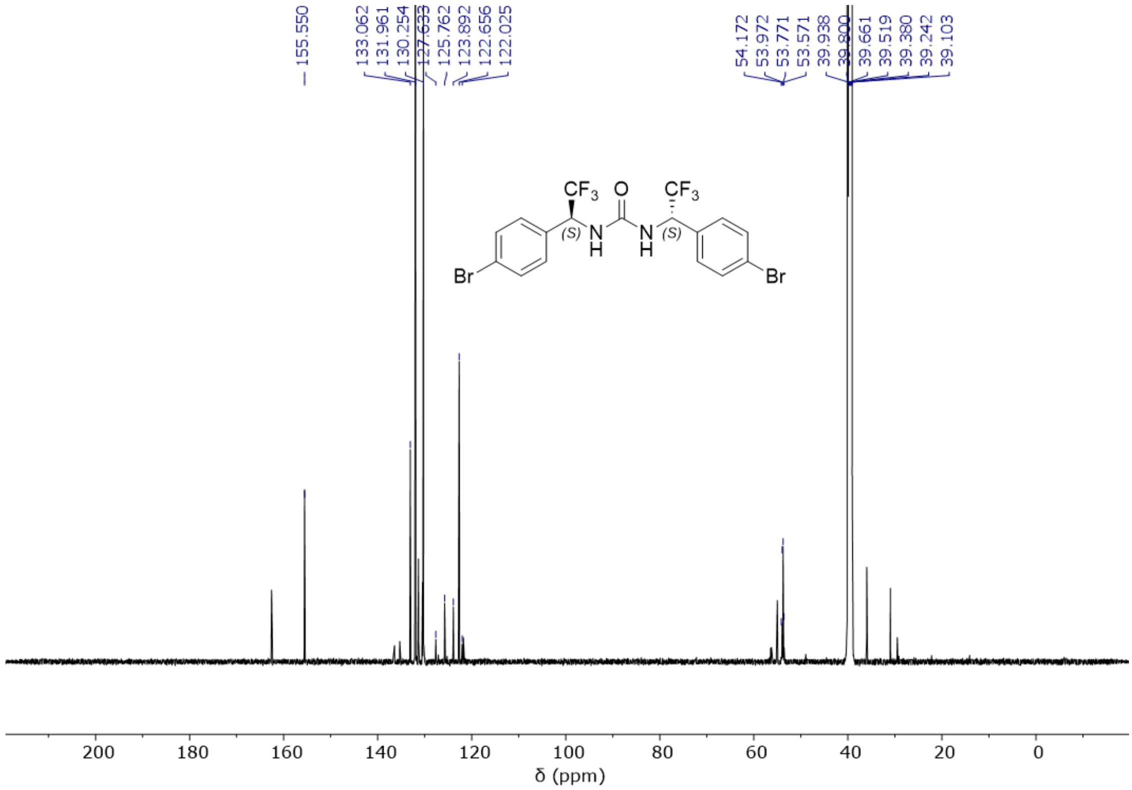

Spectrum S20. ^{13}C NMR (DMSO-d_6, 151 MHz, 298 K) spectrum of **3g**.

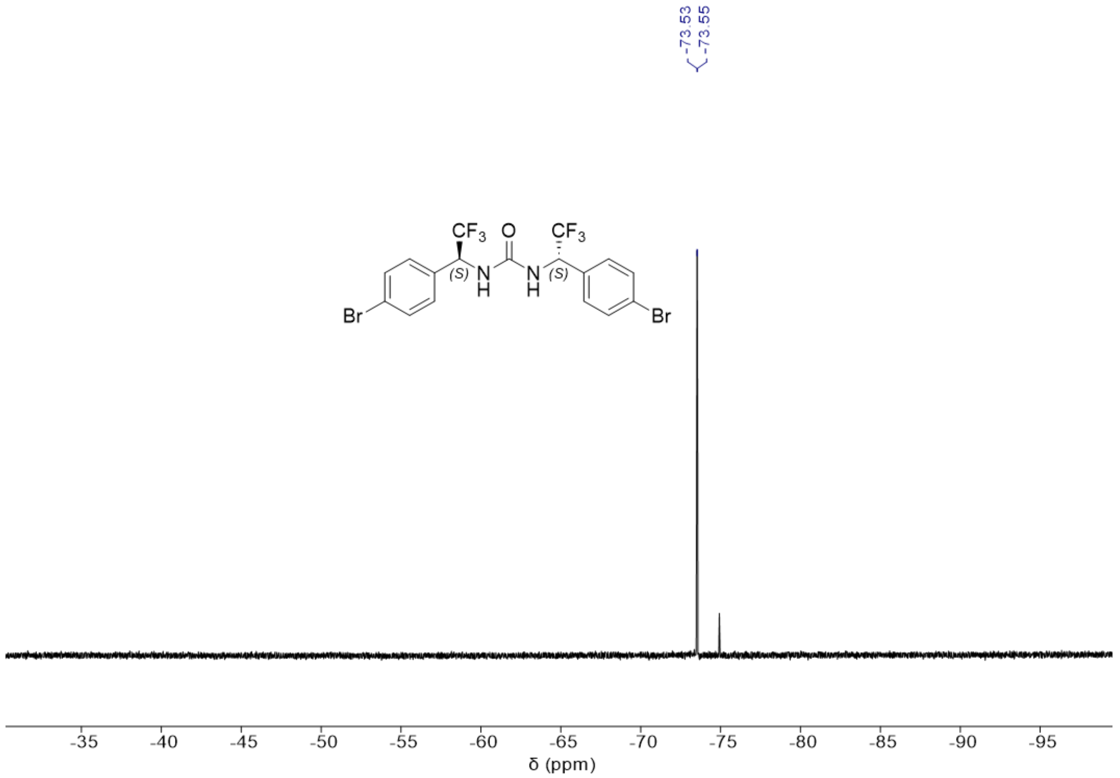

Spectrum S21. ^{19}F NMR (DMSO-d_6, 471 MHz, 298 K) spectrum of **3g**.

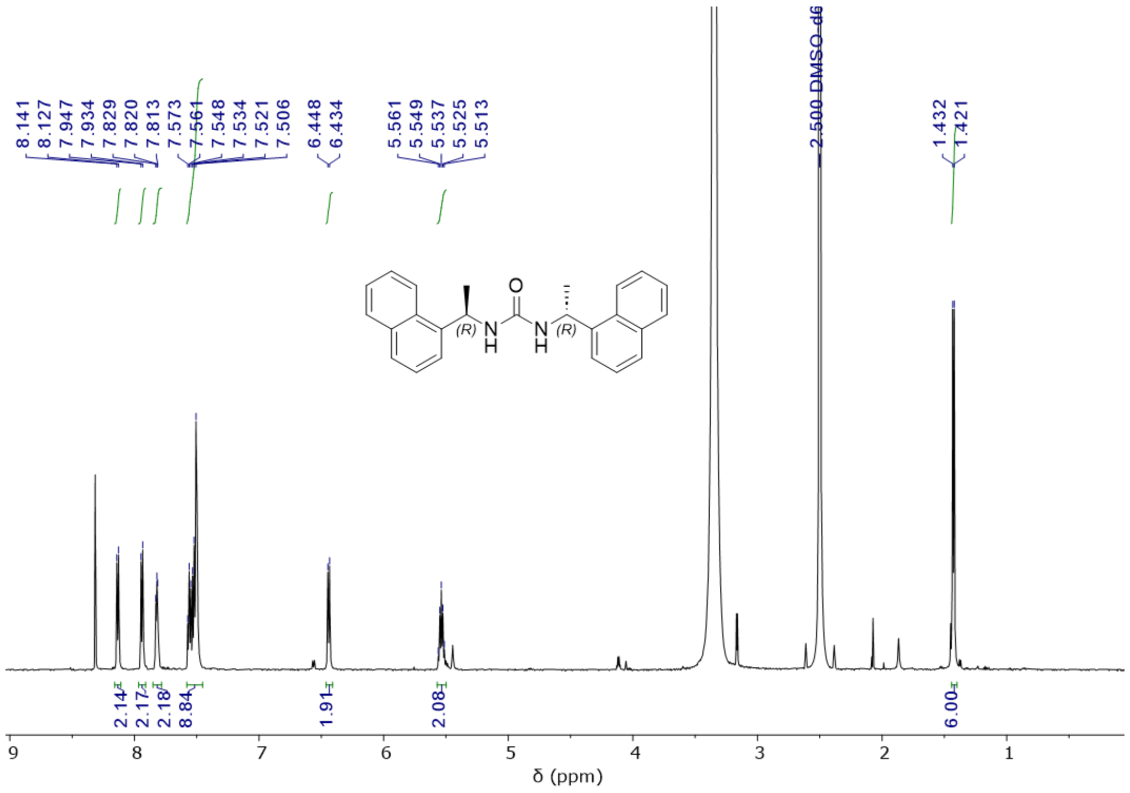

Spectrum S22. ^{1}H NMR (DMSO-d_6, 600 MHz, 298 K) spectrum of **3h**.

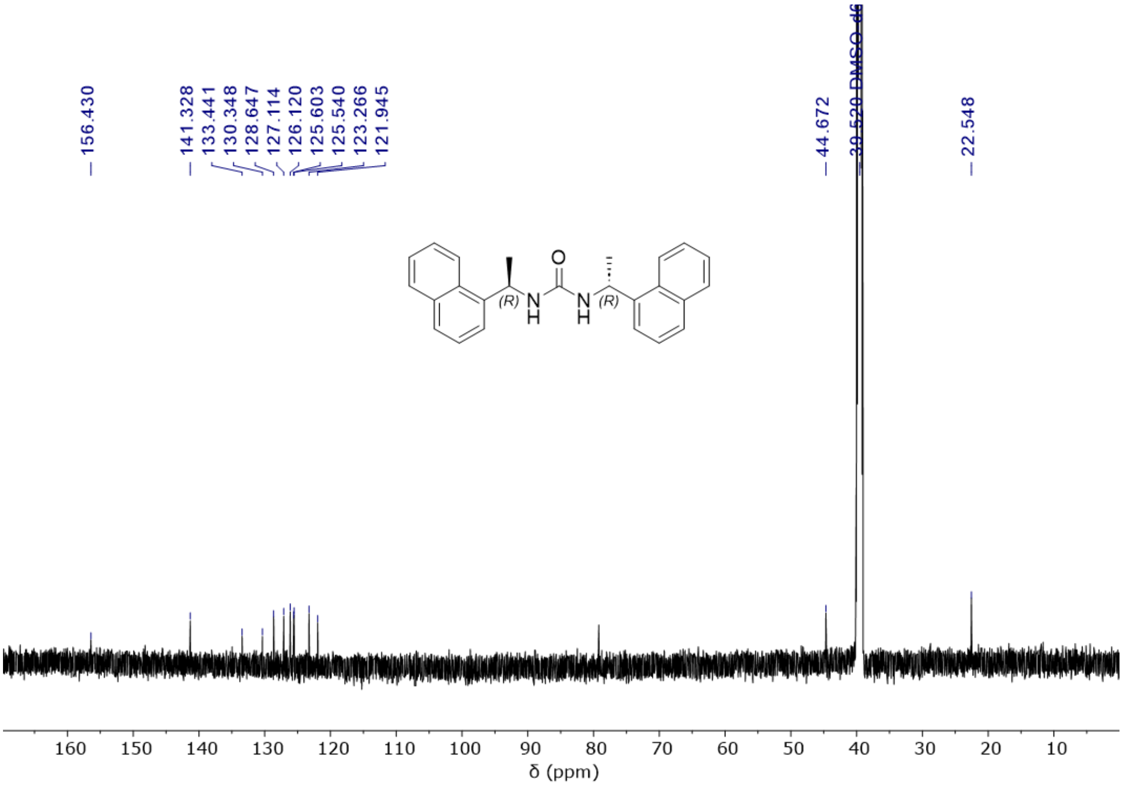

Spectrum S23. ^{13}C NMR (DMSO-*d*$_6$, 151 MHz, 298 K) spectrum of **3h**.

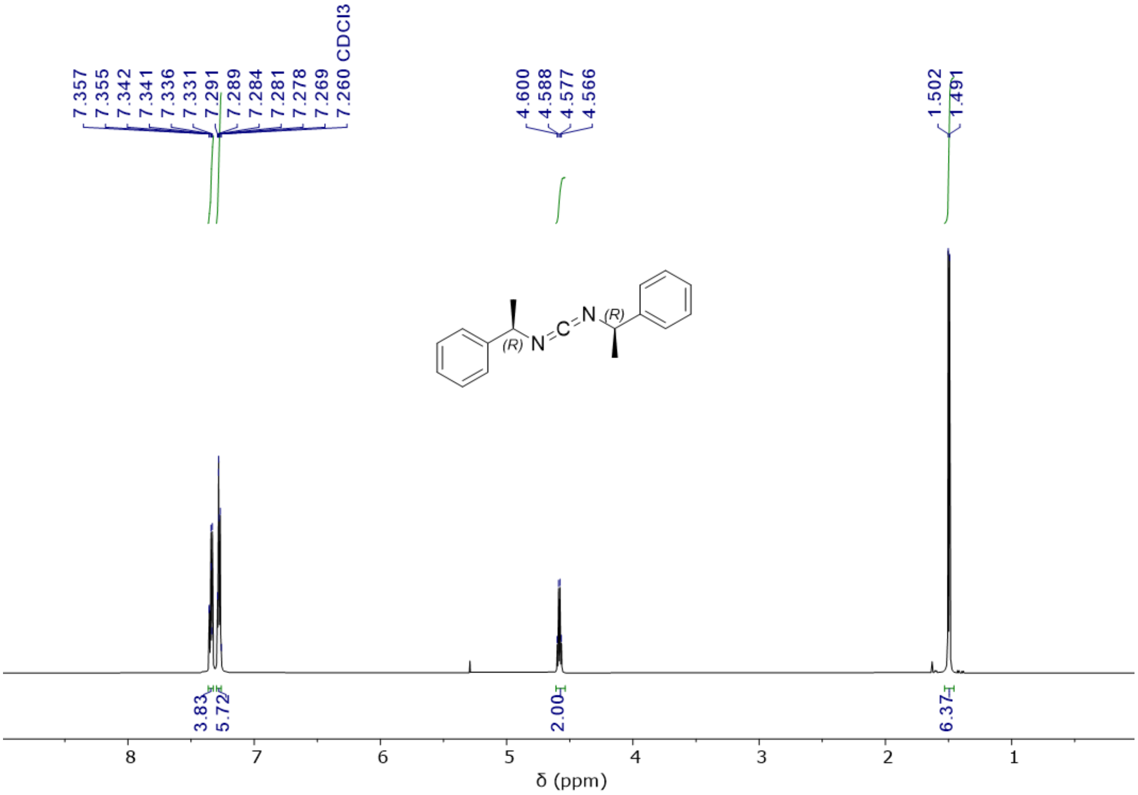

Spectrum S24. ^{1}H NMR (CDCl$_3$, 600 MHz, 298 K) spectrum of **2a**.

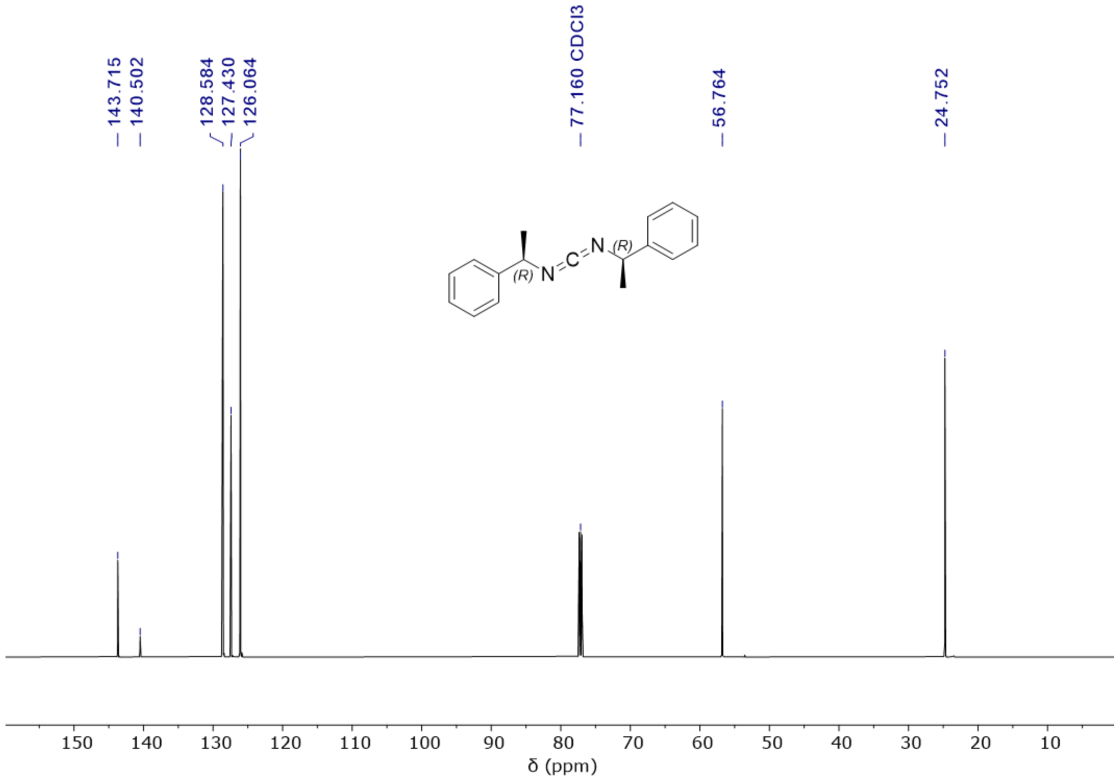

Spectrum S25. ^{13}C NMR ($CDCl_3$, 151 MHz, 298 K) spectrum of **2a**.

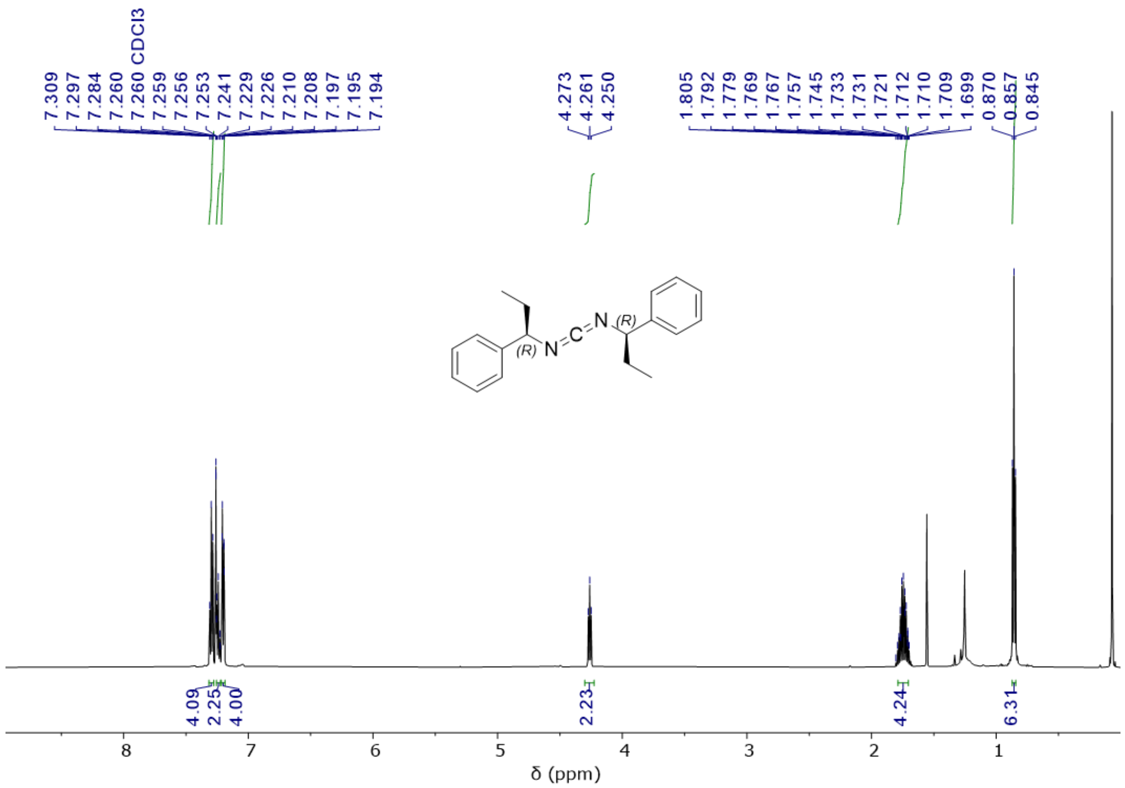

Spectrum S26. ^{1}H NMR ($CDCl_3$, 600 MHz, 298 K) spectrum of **2b**.

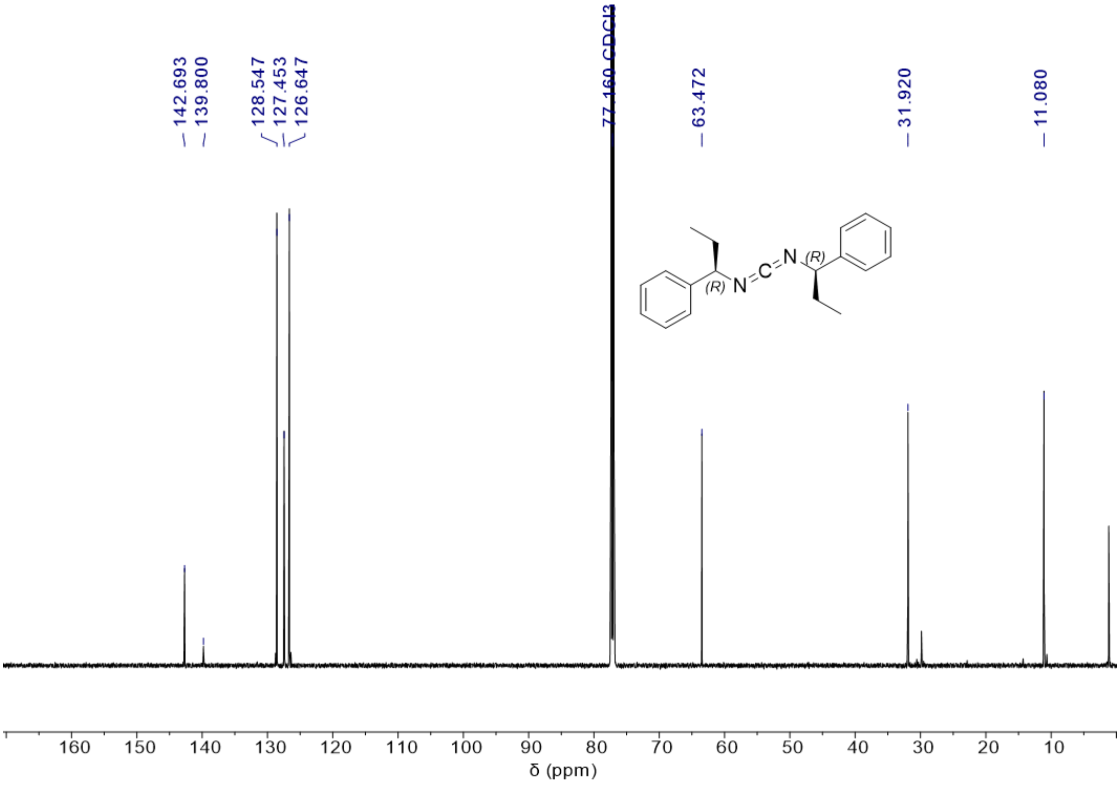

Spectrum S27. ^{13}C NMR ($CDCl_3$, 151 MHz, 298 K) spectrum of **2b**.

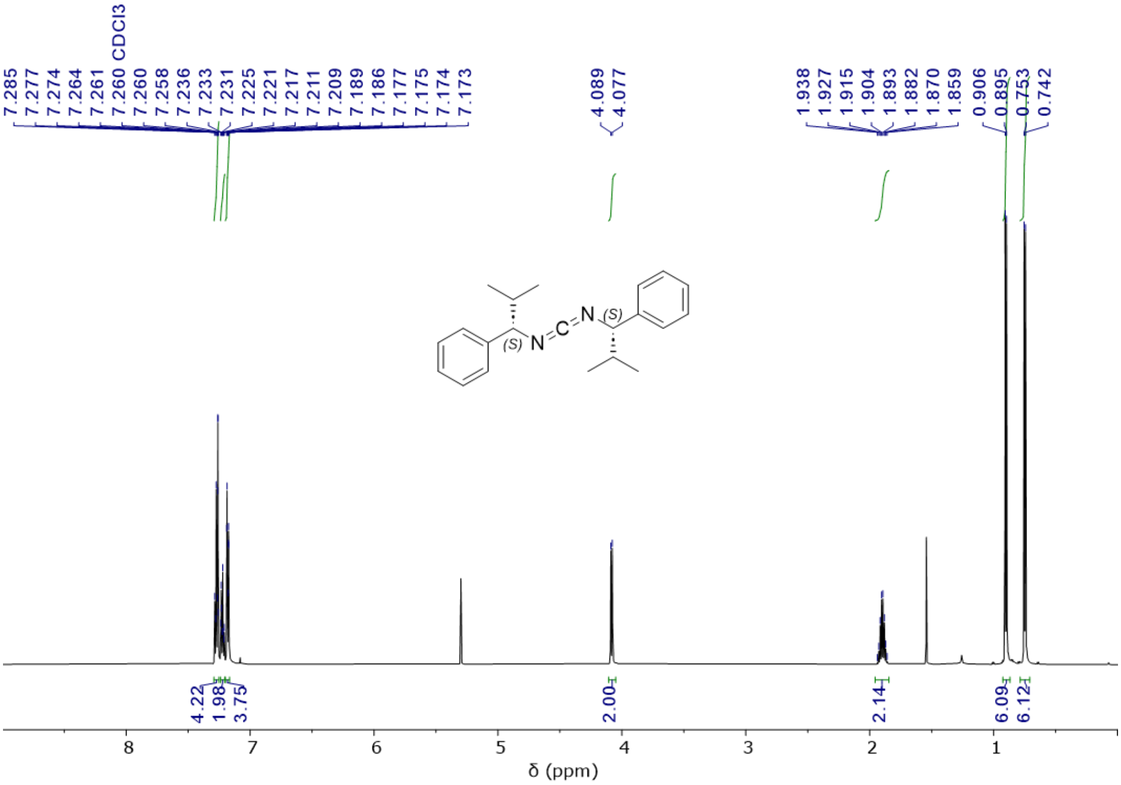

Spectrum S28. ^{1}H NMR ($CDCl_3$, 600 MHz, 298 K) spectrum of **2c**.

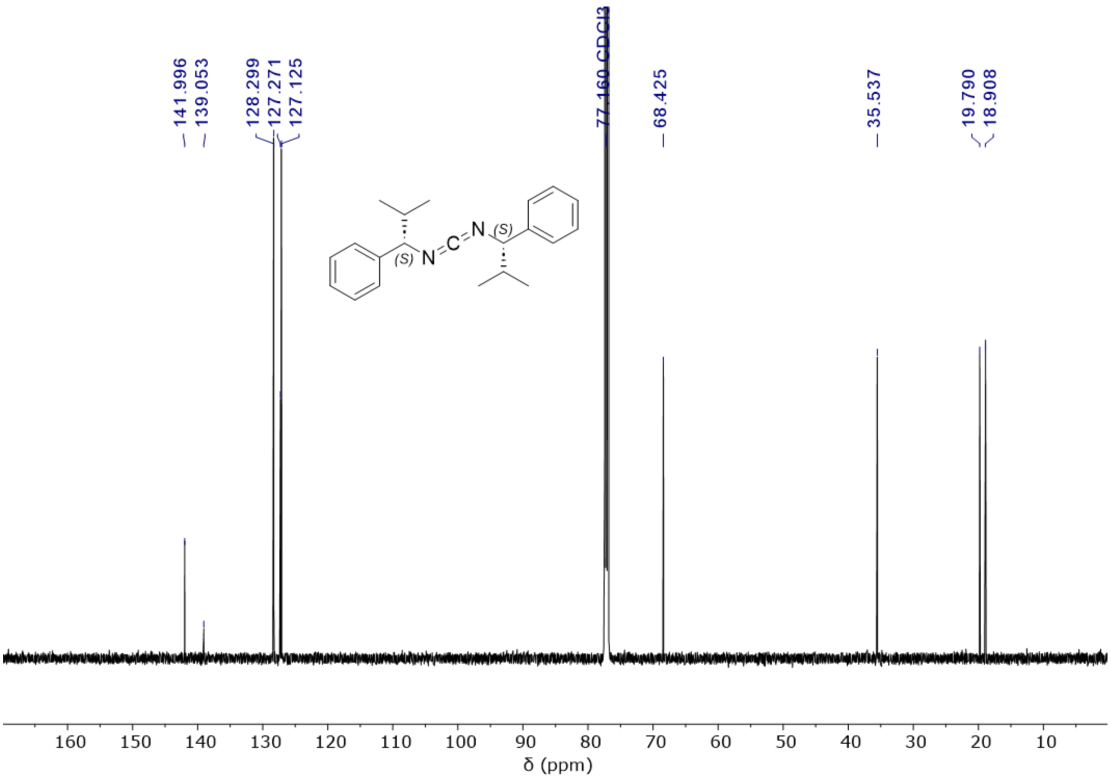

Spectrum S29. ^{13}C NMR ($CDCl_3$, 151 MHz, 298 K) spectrum of **2c**.

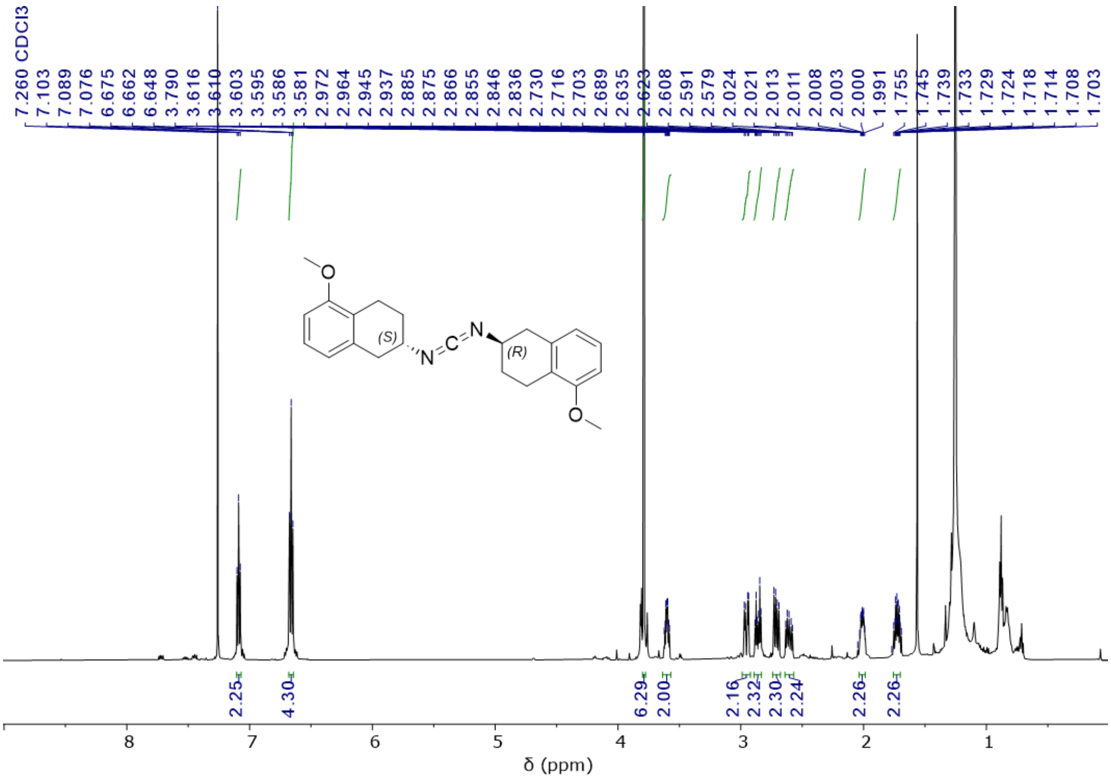

Spectrum S30. ^{1}H NMR ($CDCl_3$, 600 MHz, 298 K) spectrum of **2d**.

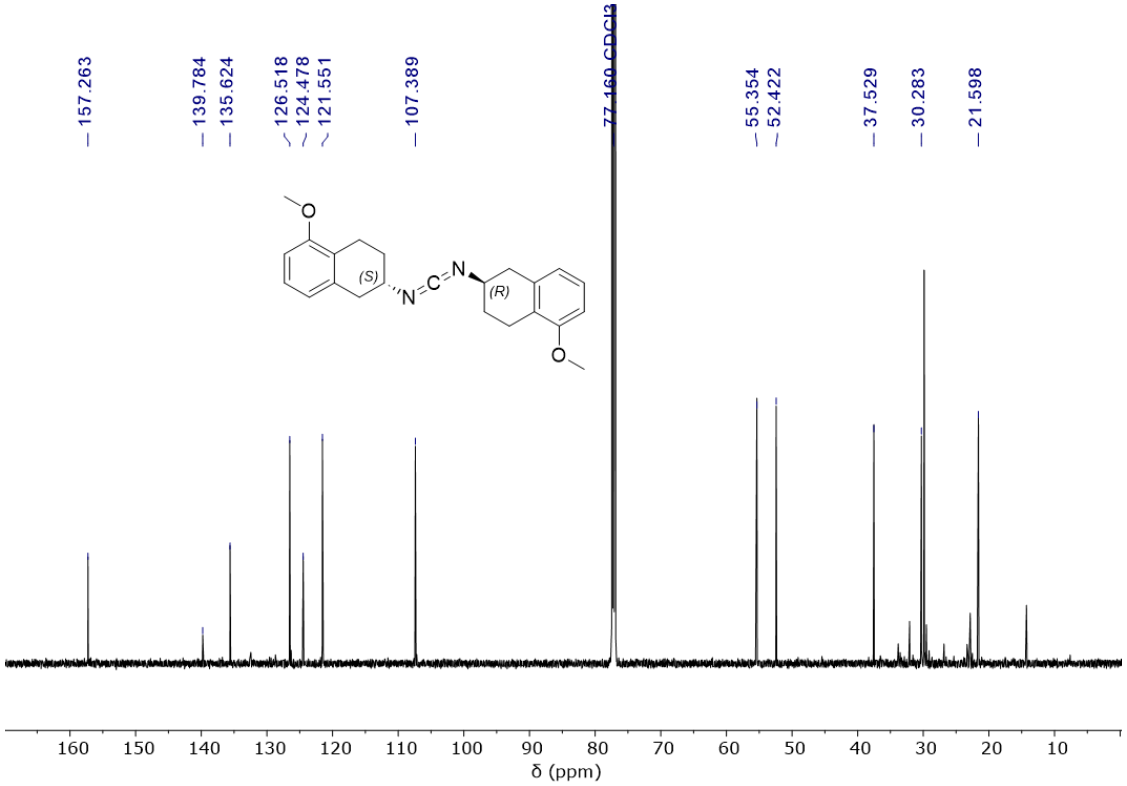

Spectrum S31. ^{13}C NMR ($CDCl_3$, 151 MHz, 298 K) spectrum of **2d**.

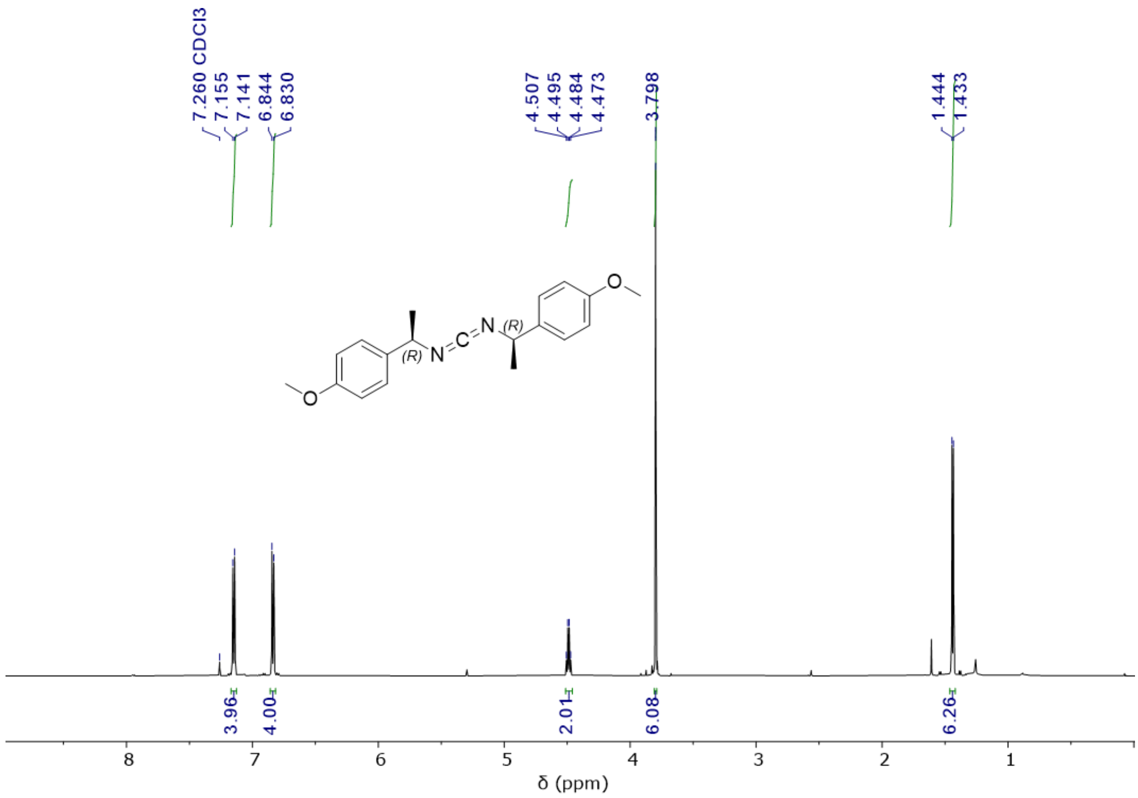

Spectrum S32. ^{1}H NMR ($CDCl_3$, 600 MHz, 298 K) spectrum of **2e**.

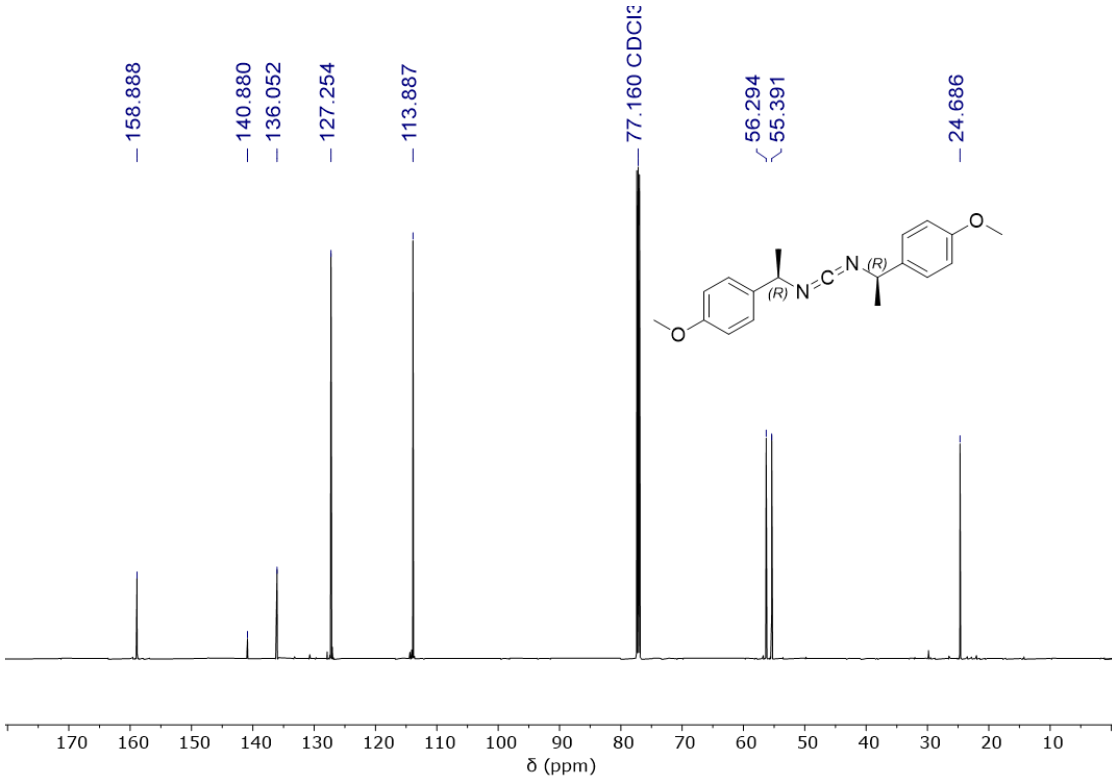

Spectrum S33. ^{13}C NMR ($CDCl_3$, 151 MHz, 298 K) spectrum of **2e**.

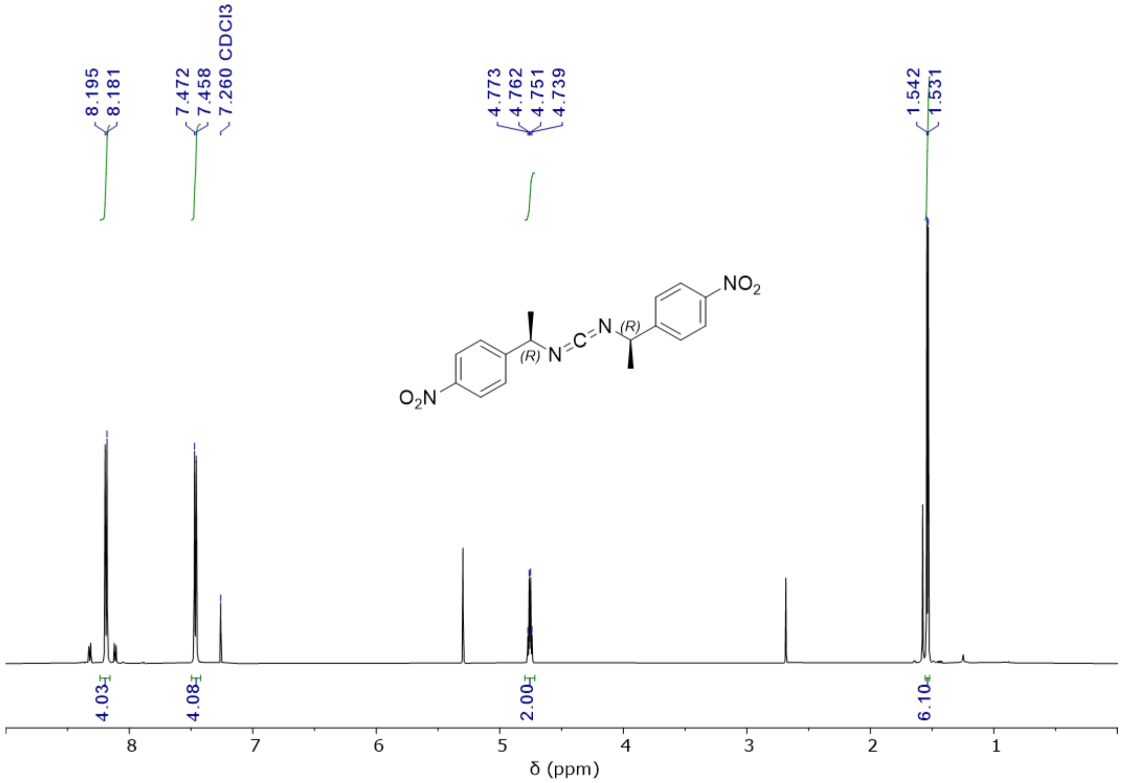

Spectrum S34. ^{1}H NMR ($CDCl_3$, 600 MHz, 298 K) spectrum of **2f**.

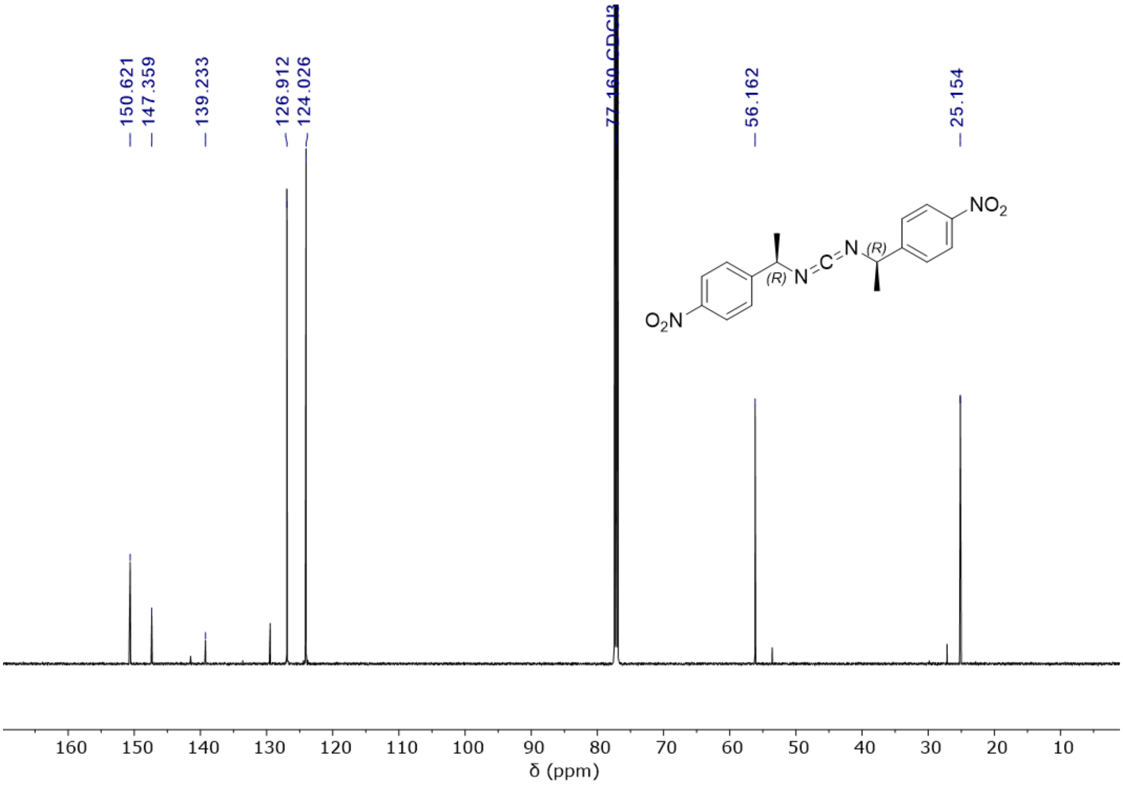

Spectrum S35. ^{13}C NMR ($CDCl_3$, 151 MHz, 298 K) spectrum of **2f**.

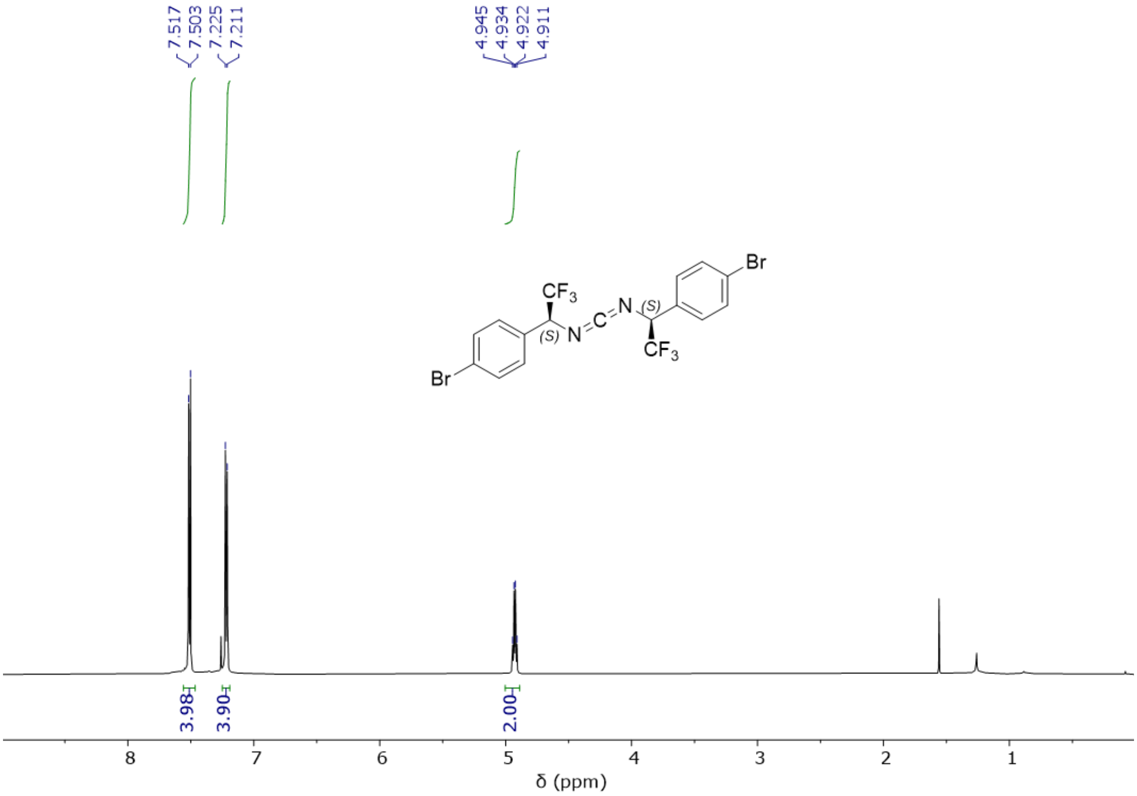

Spectrum S36. ^{1}H NMR ($CDCl_3$, 600 MHz, 298 K) spectrum of **2g**.

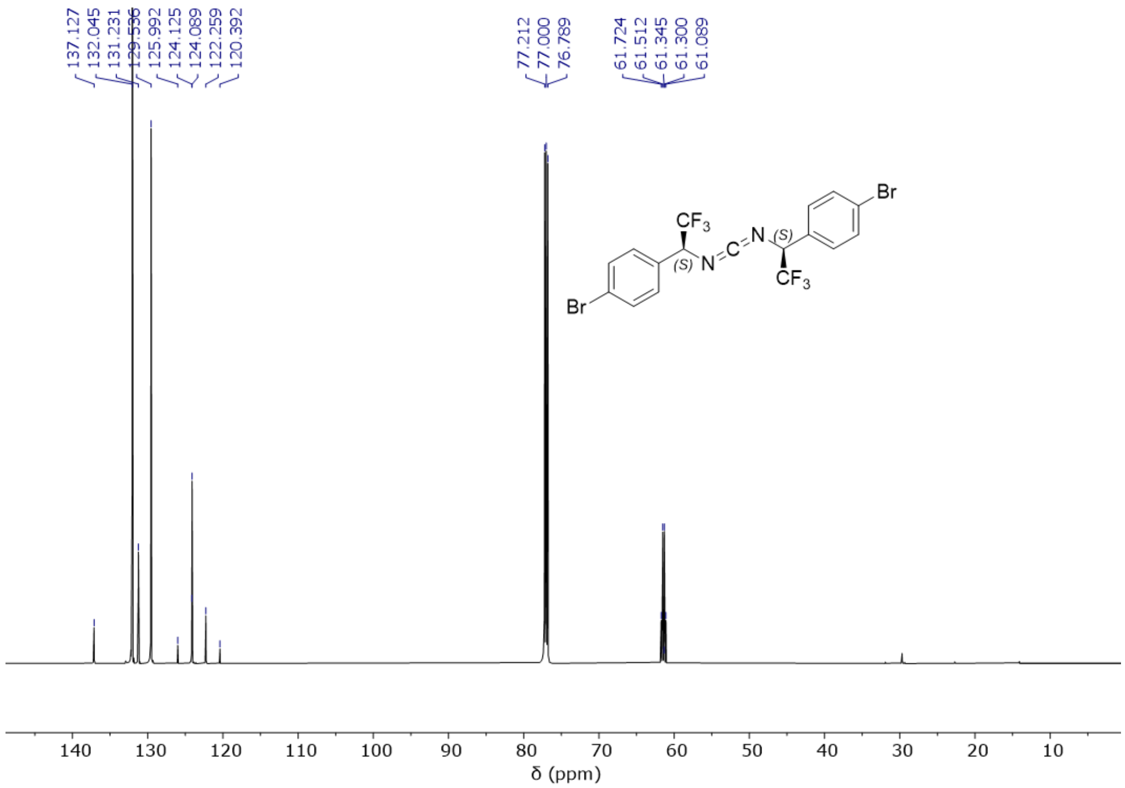

Spectrum S37. ^{13}C NMR ($CDCl_3$, 151 MHz, 298 K) spectrum of **2g**.

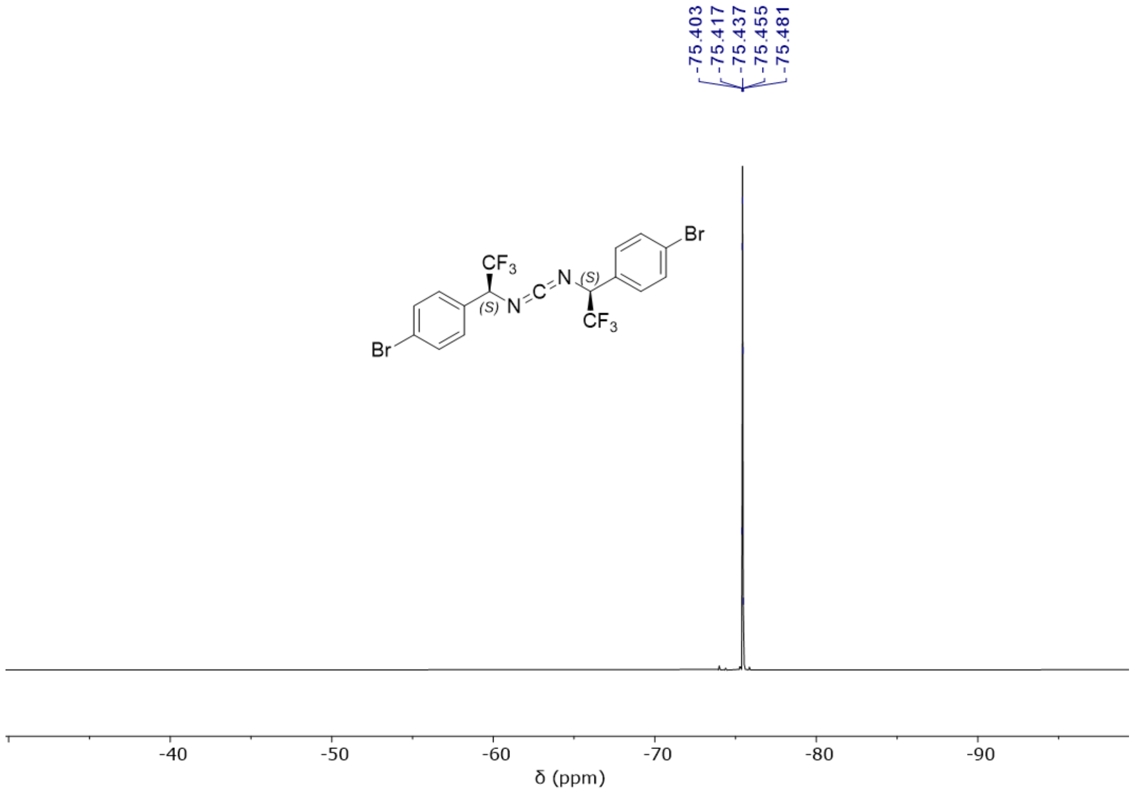

Spectrum S38. ^{19}F NMR ($CDCl_3$, 471 MHz, 298 K) spectrum of **2g**.

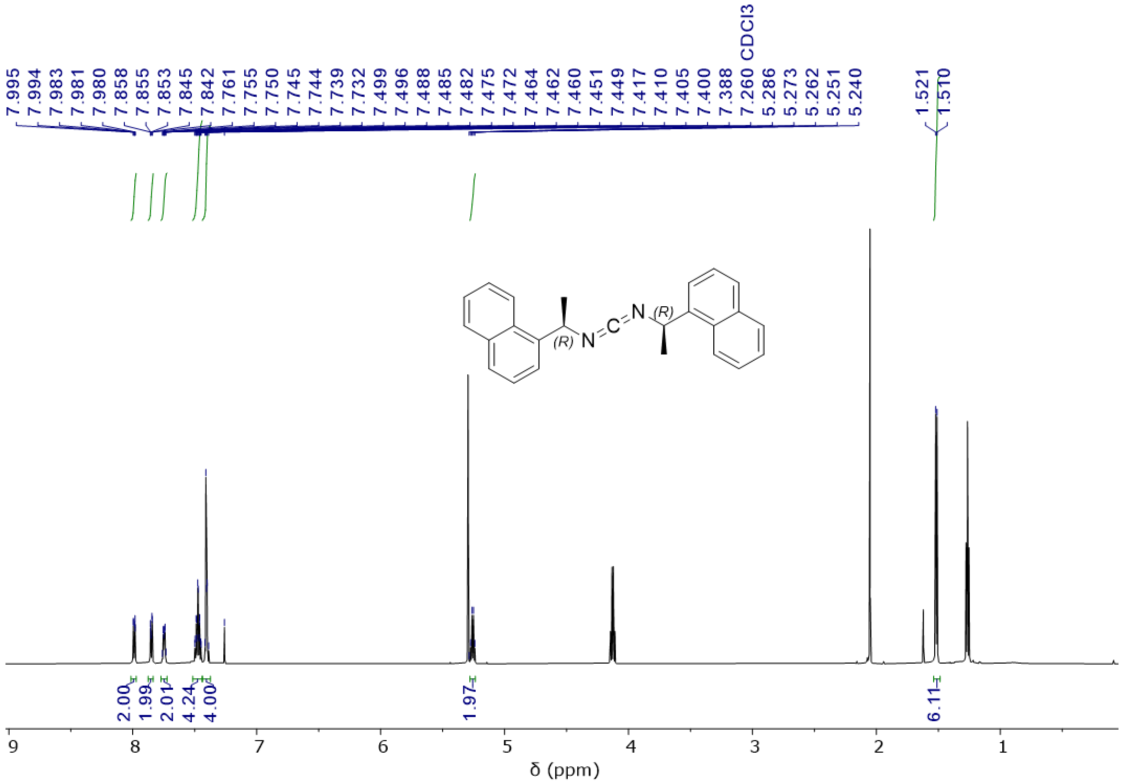

Spectrum S39. ^{1}H NMR ($CDCl_3$, 600 MHz, 298 K) spectrum of **2h**.

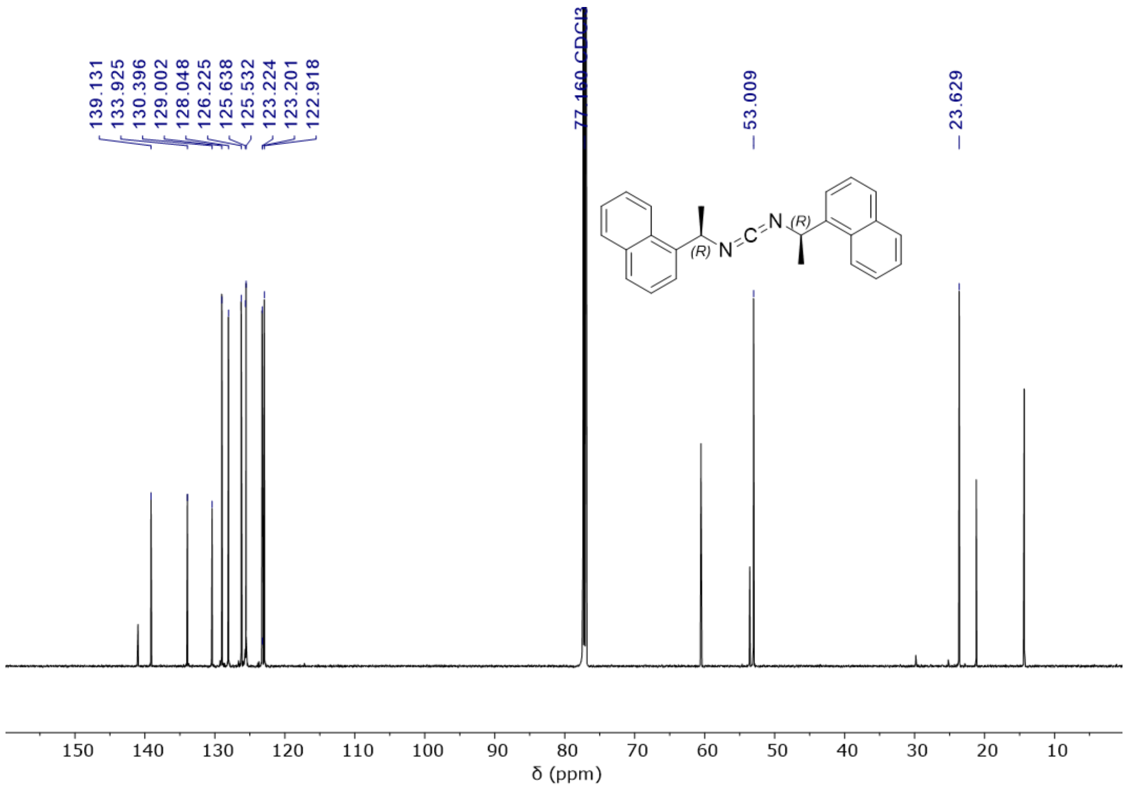

Spectrum S40. ^{13}C NMR ($CDCl_3$, 151 MHz, 298 K) spectrum of **2h**.

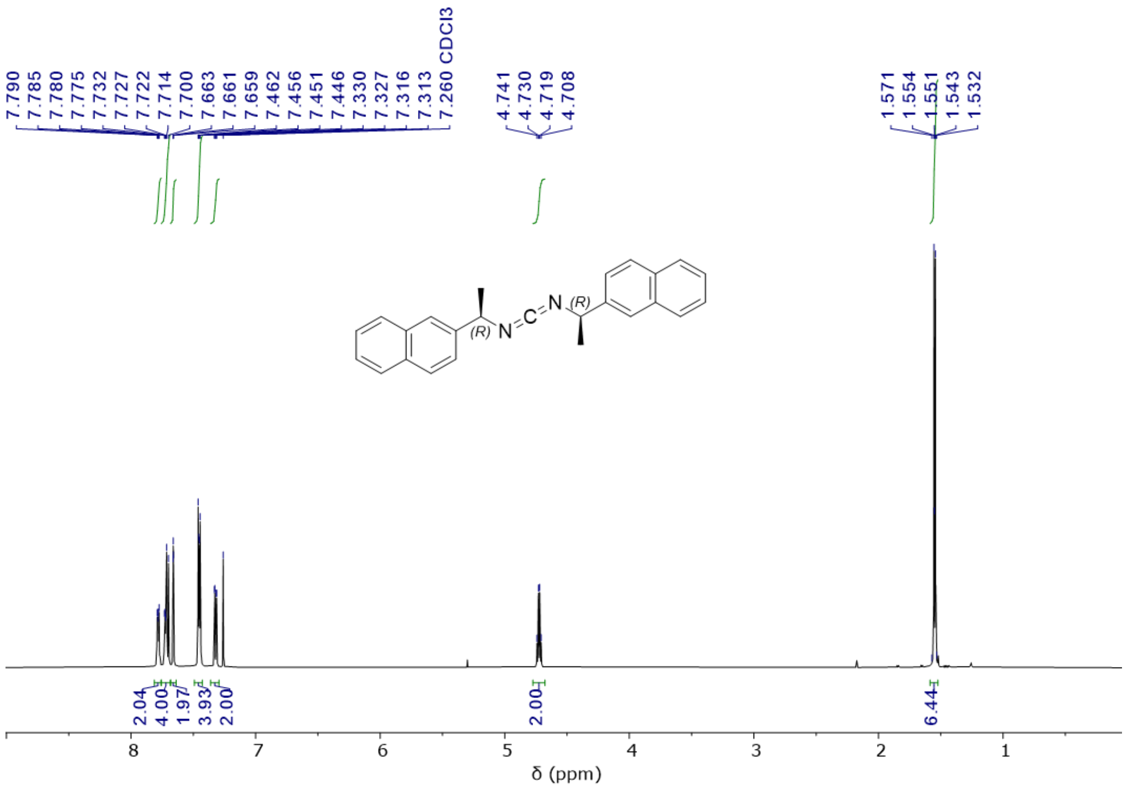

Spectrum S41. ^{1}H NMR ($CDCl_3$, 600 MHz, 298 K) spectrum of **2i**.

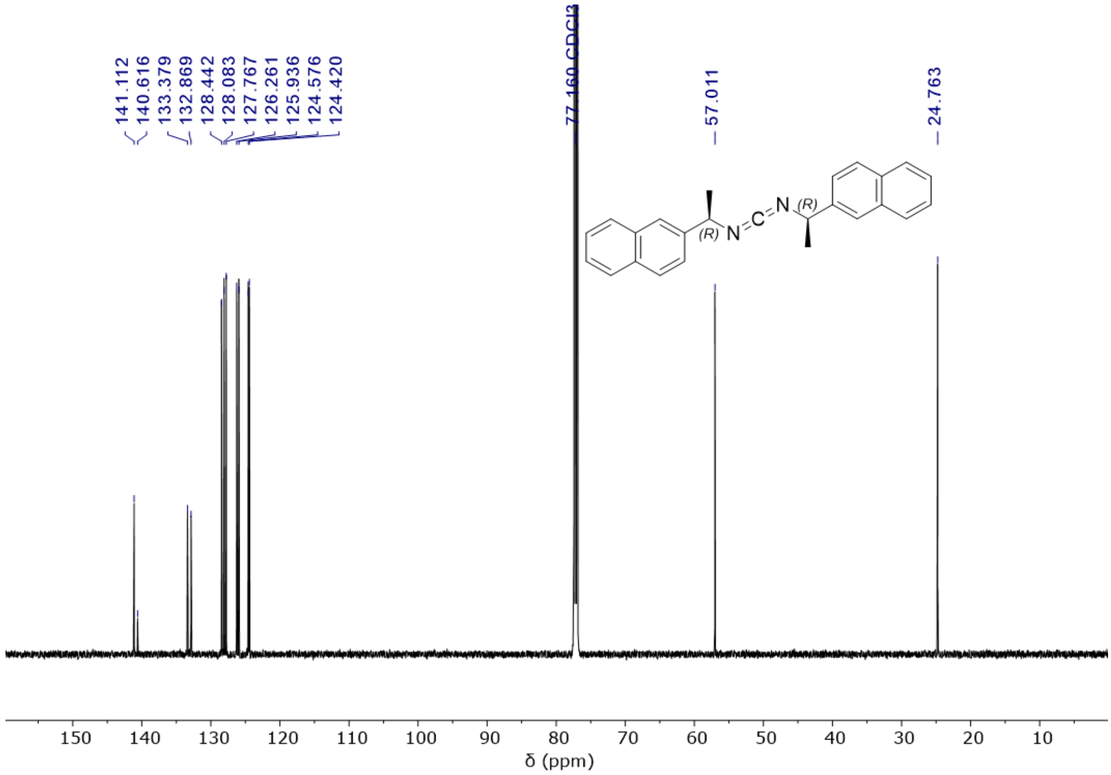

Spectrum S42. ^{13}C NMR ($CDCl_3$, 151 MHz, 298 K) spectrum of **2i**.

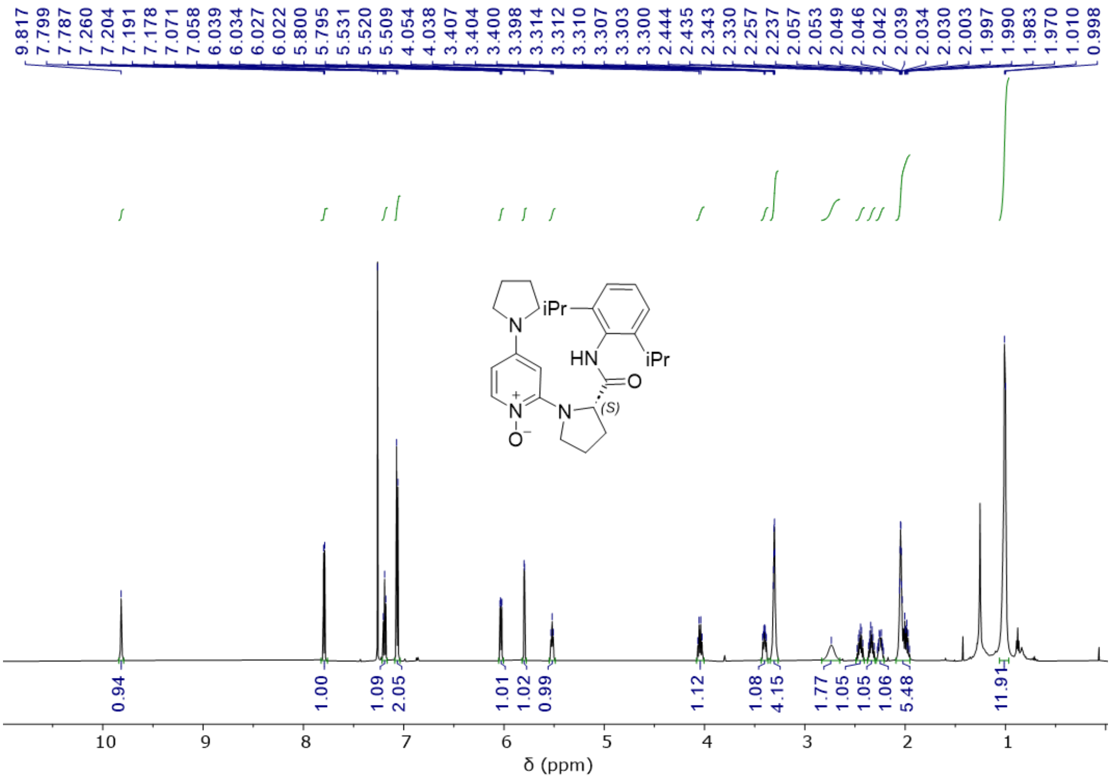

Spectrum S43. ^{1}H NMR ($CDCl_3$, 600 MHz, 298 K) spectrum of (*S*)-**4b**.

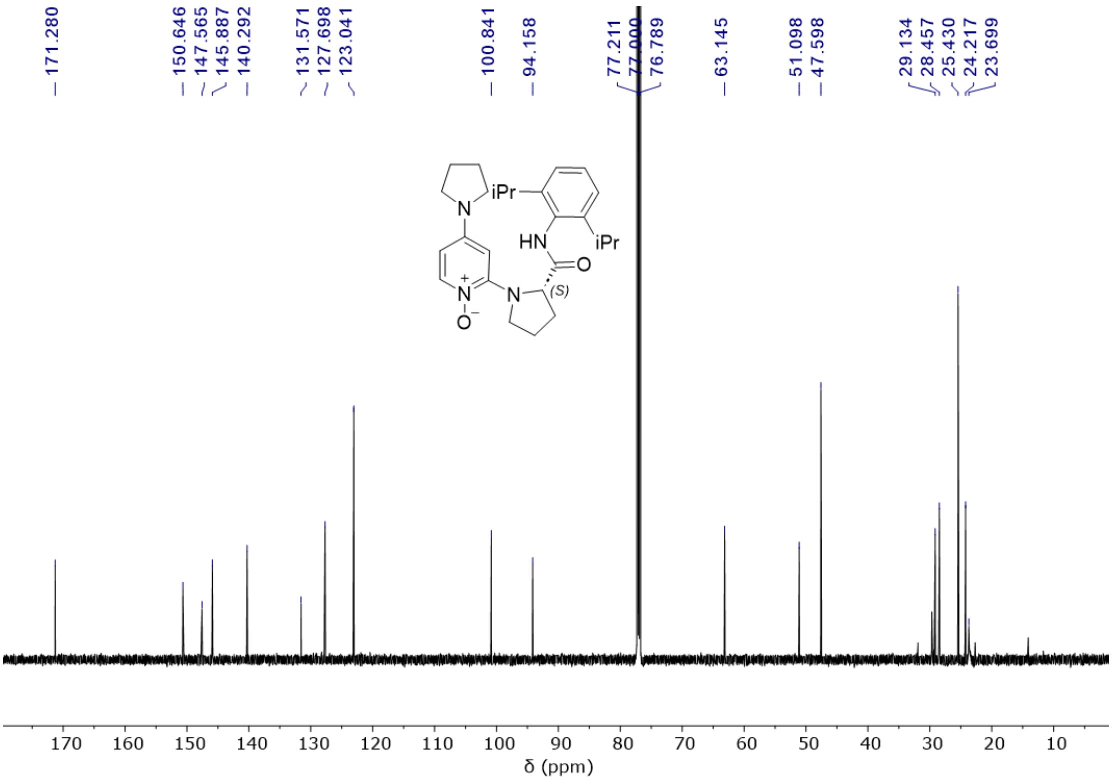

Spectrum S44. ^{13}C NMR ($CDCl_3$, 151 MHz, 298 K) spectrum of (*S*)-**4b**.

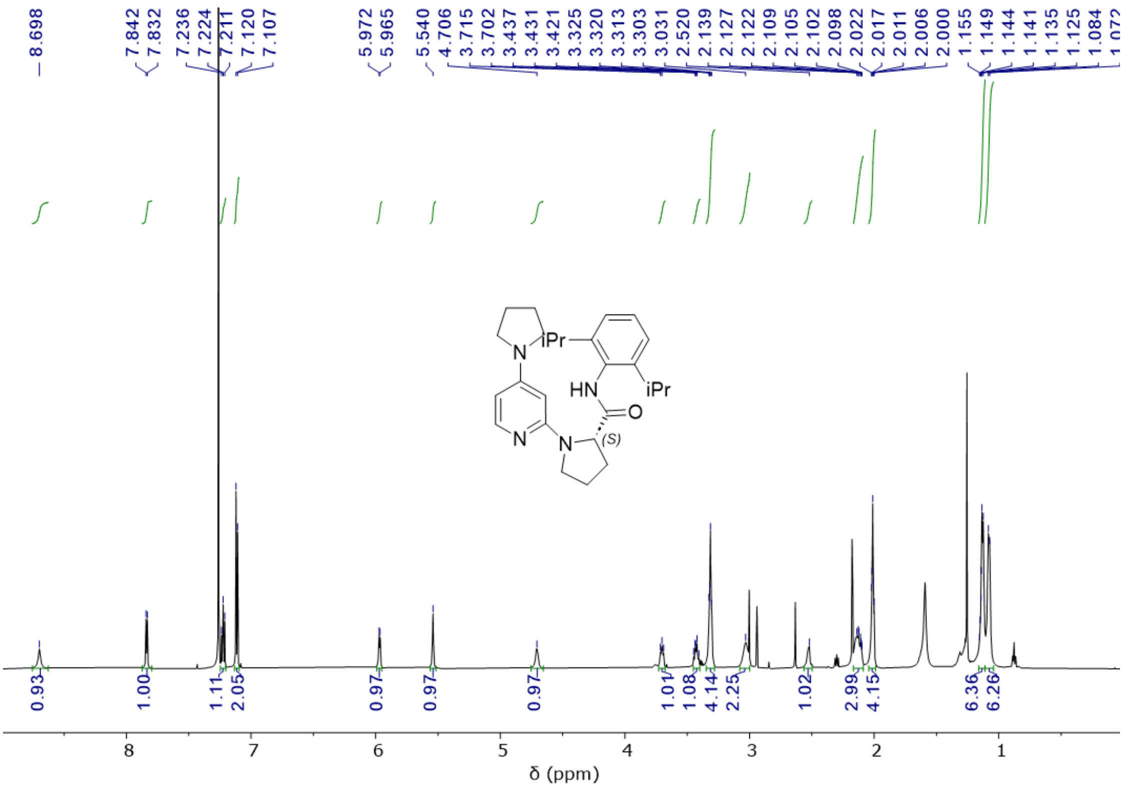

Spectrum S45. ^{1}H NMR ($CDCl_3$, 600 MHz, 298 K) spectrum of (*S*)-**4c**.

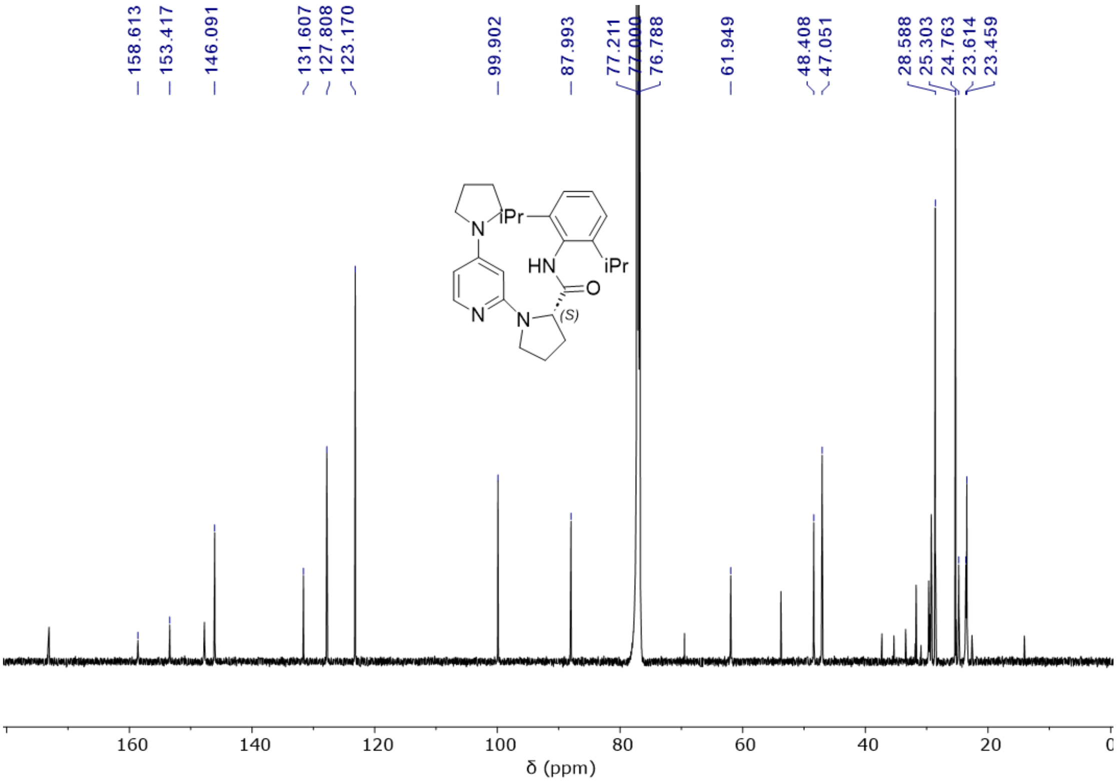

Spectrum S46. ^{13}C NMR ($CDCl_3$, 151 MHz, 298 K) spectrum of (*S*)-**4c**.

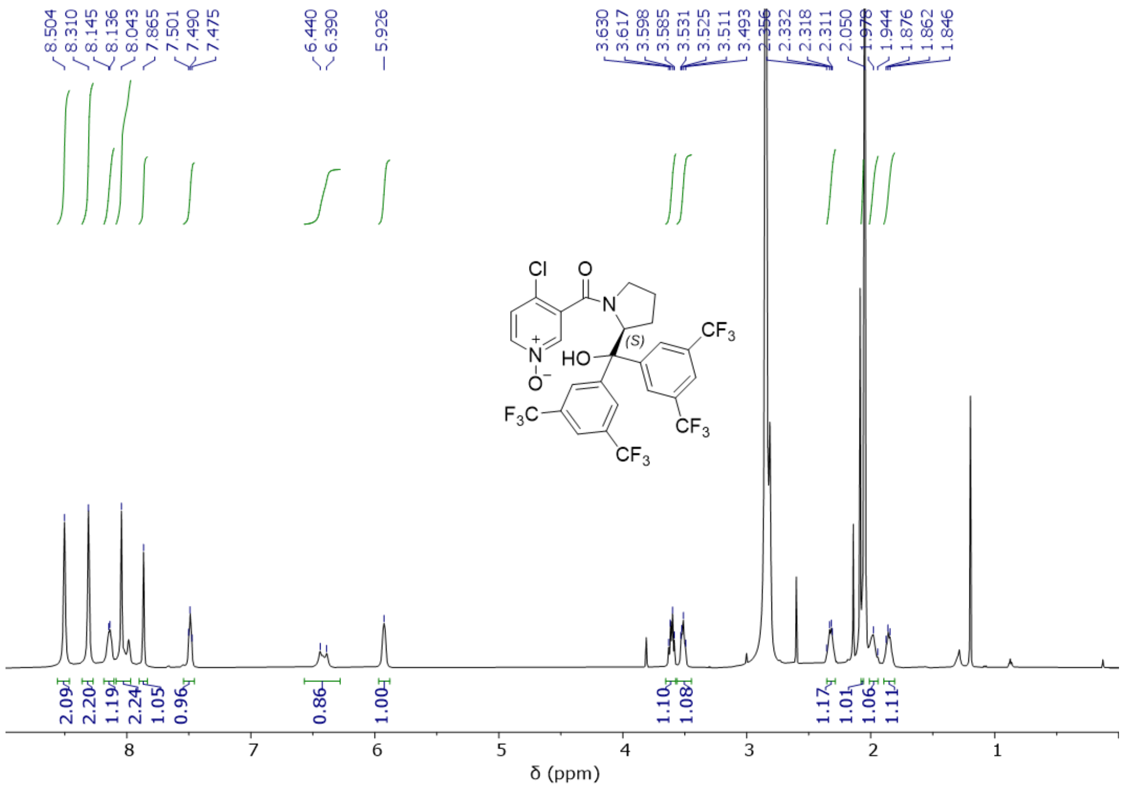

Spectrum S47. ^{1}H NMR (Acetone-d_6, 600 MHz, 298 K) spectrum of **S2**.

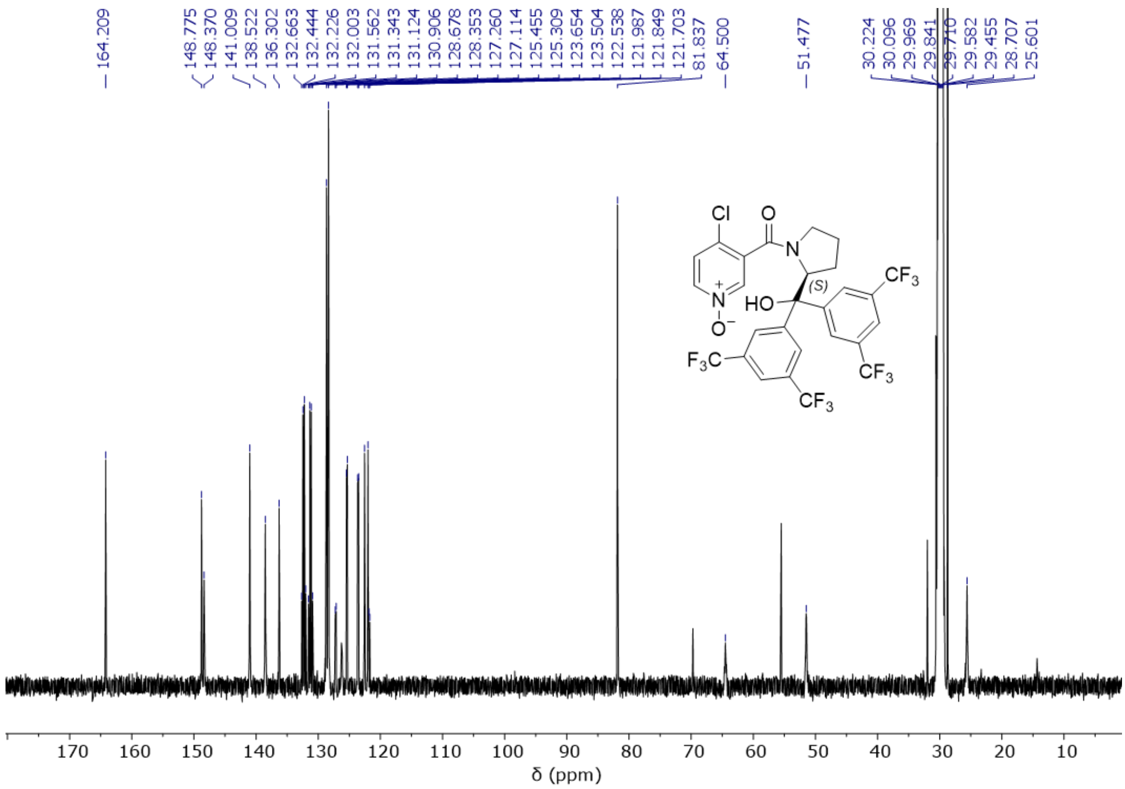

Spectrum S48. ^{13}C NMR (Acetone-d_6, 151 MHz, 298 K) spectrum of **S2**.

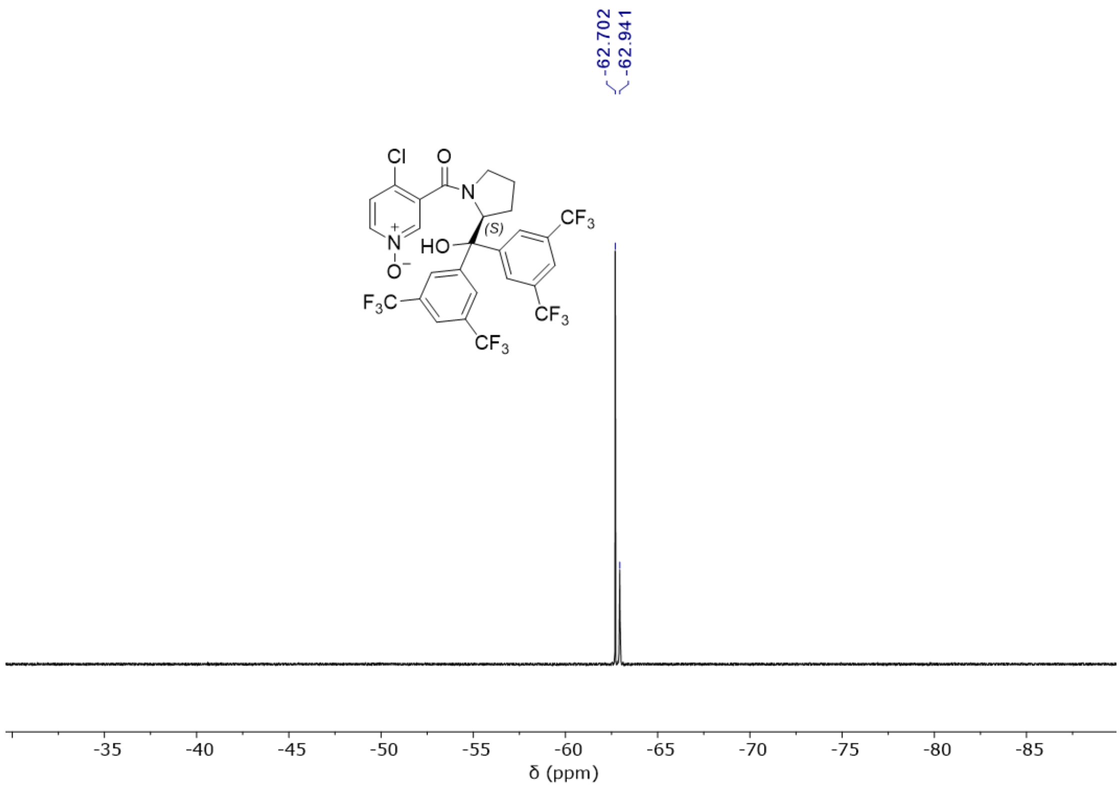

Spectrum S49. ^{19}F NMR ($CDCl_3$, 471 MHz, 298 K) spectrum of **S2**.

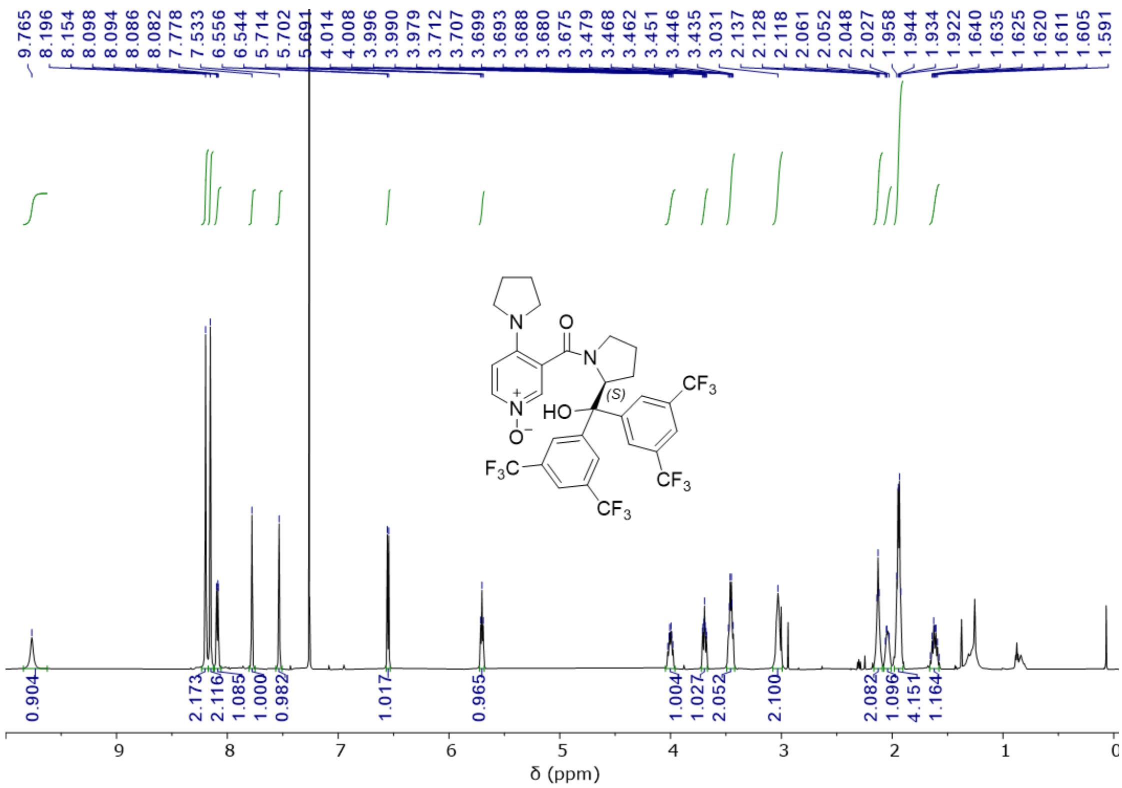

Spectrum S50. ^{1}H NMR ($CDCl_3$, 600 MHz, 298 K) spectrum of (*S*)-**4d**.

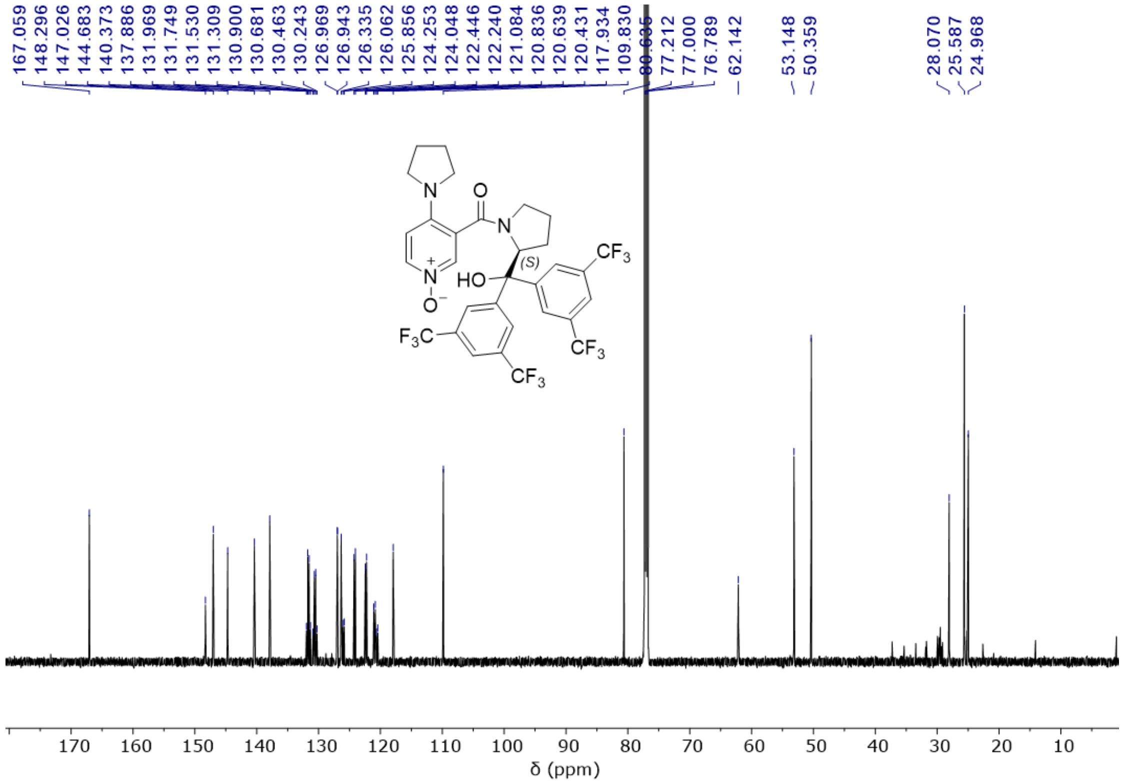

Spectrum S51. ^{13}C NMR ($CDCl_3$, 151 MHz, 298 K) spectrum of (*S*)-**4d**.

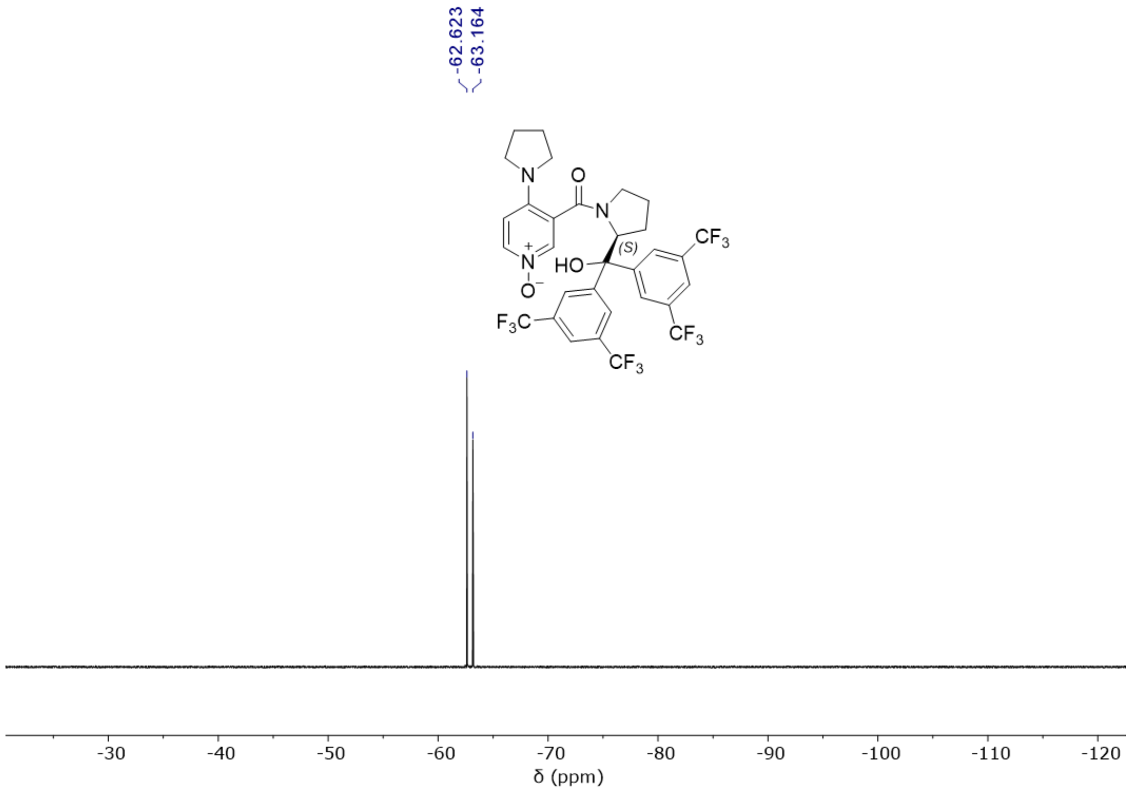

Spectrum S52. ^{19}F NMR ($CDCl_3$, 471 MHz, 298 K) spectrum of (*S*)-**4d**.

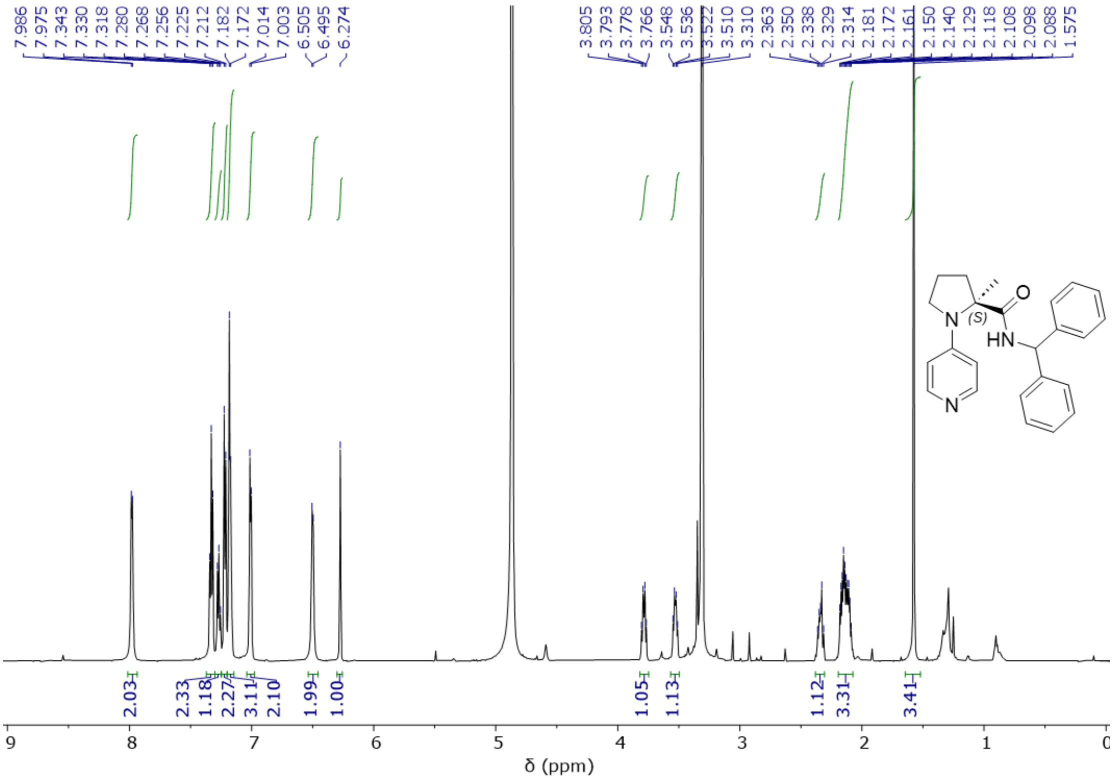

Spectrum S53. ^{1}H NMR (CD_3OD, 600 MHz, 298 K) spectrum of (*S*)-**4h**.

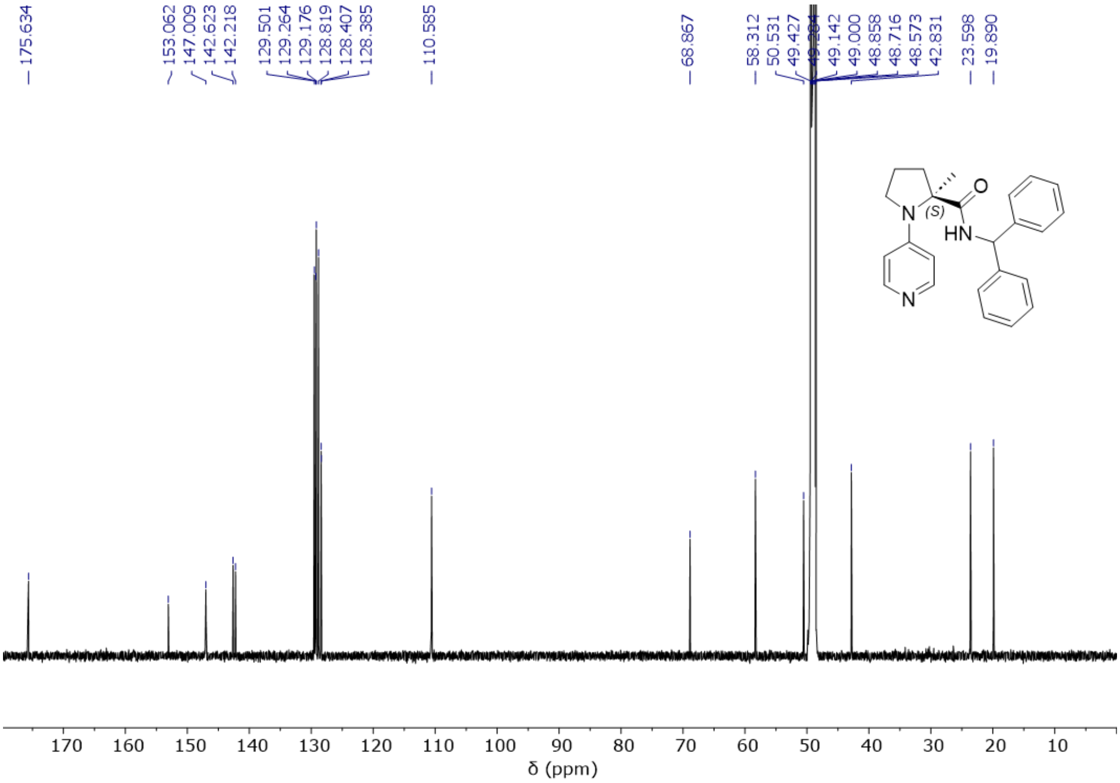

Spectrum S54. ^{13}C NMR (CD_3OD, 151 MHz, 298 K) spectrum of (*S*)-**4h**.

S12. References

S1 Borsley, S.; Kreidt, E.; Leigh, D. A.; Roberts, B. M. W. Autonomous fuelled directional rotation about a covalent single bond. *Nature* **2022**, *604*, 80-85. DOI: 10.1038/s41586-022-04450-5

S2 Brunner, T. S.; Benndorf, P.; Gamer, M. T.; Knöfel, N.; Gugau, K.; Roesky, P. W. Enantiopure amidinate complexes of the rare-earth elements. *Organometallics* **2016**, *35*, 3474–3487. DOI: 10.1021/acs.organomet.6b00523

S3 Sheehan, J. C.; Hlavka, J. J. The use of water-soluble and basic carbodiimides in peptide synthesis. *J. Org. Chem*. **1956**, *21*, 439-441. DOI: 10.1021/jo01110a017

S4 Ó Dálaigh, C.; Connon, S. J. Nonenzymatic acylative kinetic resolution of Baylis−Hillman adducts. *J. Org. Chem*. **2007**, *72*, 7066-7069. DOI: 10.1021/jo071223b

S5 Xie, M.-S.; Huang, B.; Li, N.; Tian, Y.; Wu, X.-X.; Deng, Y.; Qu, G.-R.; Guo, H.-M. Rational design of 2-substituted DMAP-*N*-oxides as acyl transfer catalysts: dynamic kinetic resolution of azlactones**.** *J. Am. Chem. Soc.* **2020**, *142*, 19226–19238. DOI: 10.1021/jacs.0c09075

S6 Yu, Z.; Liu, X.; Dong, Z.; Xie, M.; Feng, X. An *N*,*N′*-Dioxide/In(OTf)$_3$ catalyst for the asymmetric hetero-Diels–Alder reaction between Danishefsky's dienes and aldehydes: application in the total synthesis of Triketide. *Angew. Chem. Int. Ed.* **2008**, *47*, 1308–1311. DOI: 10.1002/anie.200704759

S7 Priem, G.; Anson, M. S.; Macdonald, S. J. F.; Pelotier. B.; Campbella, I. B. A simple and effective synthetic approach to chiral 4-pyridinyl proline derivatives. *Tetrahedron* **2002**, *43*, 6001-6003. DOI: 10.1016/S0040-4039(02)01255-8

S8 Gallagher, J. M.; Roberts, B. M. W.; Borsley, S.; Leigh, D. A. Conformational selection accelerates catalysis by an organocatalytic molecular motor. *Chem* **2024**, *10*, 855–866. DOI: 10.1016/j.chempr.2023.10.019

S9 Binks, L.; Borsley, S.; Gingrich, T. R.; Leigh, D. A.; Penocchio, E.; Roberts, B. M. W. The role of kinetic asymmetry and power strokes in an information ratchet. *Chem* **2023**, *9*, 2902–2917. DOI: 10.1016/j.chempr.2023.05.035

S10 Penocchio, E.; Bachir, A.; Credi, A.; Astumian, R. D.; Ragazzon, G. Analysis of kinetic asymmetry in a multi-cycle chemical reaction network establishes the principles for autonomous compartmentalized molecular ratchets. *Chem* **2024***, 10,* 3644–3655*.* DOI: 10.1016/j.chempr.2024.07.038

S11 Marchetti, T.; Roberts, B. M. W.; Frezzato, D.; Prins, L. J. A minimalistic covalent bond-forming chemical reaction cycle that consumes adenosine diphosphate. *Angew. Chem. Int. Ed*. **2024**, *63*, e202402965. DOI: 10.1002/anie.202402965
